# PRISMA 2020 explanation and elaboration: updated guidance and exemplars for reporting systematic reviews

**DOI:** 10.1136/bmj.n160

**Published:** 2021-03-29

**Authors:** Matthew J Page, David Moher, Patrick M Bossuyt, Isabelle Boutron, Tammy C Hoffmann, Cynthia D Mulrow, Larissa Shamseer, Jennifer M Tetzlaff, Elie A Akl, Sue E Brennan, Roger Chou, Julie Glanville, Jeremy M Grimshaw, Asbjørn Hróbjartsson, Manoj M Lalu, Tianjing Li, Elizabeth W Loder, Evan Mayo-Wilson, Steve McDonald, Luke A McGuinness, Lesley A Stewart, James Thomas, Andrea C Tricco, Vivian A Welch, Penny Whiting, Joanne E McKenzie

**Affiliations:** 1 School of Public Health and Preventive Medicine, Monash University, Melbourne, Australia; 2 Centre for Journalology, Clinical Epidemiology Program, Ottawa Hospital Research Institute, Ottawa, Canada; School of Epidemiology and Public Health, Faculty of Medicine, University of Ottawa, Ottawa, Canada; 3 Department of Clinical Epidemiology, Biostatistics and Bioinformatics, Amsterdam University Medical Centres, University of Amsterdam, Amsterdam, Netherlands; 4 Université de Paris, Centre of Epidemiology and Statistics (CRESS), Inserm, F 75004 Paris, France; 5 Institute for Evidence-Based Healthcare, Faculty of Health Sciences and Medicine, Bond University, Gold Coast, Australia; 6 University of Texas Health Science Center at San Antonio, San Antonio, Texas, United States; *Annals of Internal Medicine*; 7 Knowledge Translation Program, Li Ka Shing Knowledge Institute, Toronto, Canada; School of Epidemiology and Public Health, Faculty of Medicine, University of Ottawa, Ottawa, Canada; 8 Evidence Partners, Ottawa, Canada; 9 Clinical Research Institute, American University of Beirut, Beirut, Lebanon; Department of Health Research Methods, Evidence, and Impact, McMaster University, Hamilton, Ontario, Canada; 10 Department of Medical Informatics and Clinical Epidemiology, Oregon Health & Science University, Portland, Oregon, United States; 11 York Health Economics Consortium (YHEC Ltd), University of York, York, UK; 12 Clinical Epidemiology Program, Ottawa Hospital Research Institute, Ottawa, Canada; School of Epidemiology and Public Health, University of Ottawa, Ottawa, Canada; Department of Medicine, University of Ottawa, Ottawa, Canada; 13 Centre for Evidence-Based Medicine Odense, Odense University Hospital, Odense, Denmark; Department of Clinical Research, University of Southern Denmark, Odense, Denmark; Open Patient data Explorative Network, Odense University Hospital, Odense, Denmark; 14 Department of Anesthesiology and Pain Medicine, The Ottawa Hospital, Ottawa, Canada; Clinical Epidemiology Program, Blueprint Translational Research Group, Ottawa Hospital Research Institute, Ottawa, Canada; Regenerative Medicine Program, Ottawa Hospital Research Institute, Ottawa, Canada; 15 Department of Ophthalmology, School of Medicine, University of Colorado Denver, Denver, Colorado, United States; Department of Epidemiology, Johns Hopkins Bloomberg School of Public Health, Baltimore, Maryland, United States; 16 Division of Headache, Department of Neurology, Brigham and Women's Hospital, Harvard Medical School, Boston, Massachusetts, United States; Head of Research, *The BMJ*, London, UK; 17 Department of Epidemiology and Biostatistics, Indiana University School of Public Health-Bloomington, Bloomington, Indiana, United States; 18 Population Health Sciences, Bristol Medical School, University of Bristol, Bristol, UK; 19 Centre for Reviews and Dissemination, University of York, York, UK; 20 EPPI-Centre, UCL Social Research Institute, University College London, London, UK; 21 Li Ka Shing Knowledge Institute of St. Michael's Hospital, Unity Health Toronto, Toronto, Canada; Epidemiology Division of the Dalla Lana School of Public Health and the Institute of Health Management, Policy, and Evaluation, University of Toronto, Toronto, Canada; Queen's Collaboration for Health Care Quality Joanna Briggs Institute Centre of Excellence, Queen's University, Kingston, Canada; 22 Methods Centre, Bruyère Research Institute, Ottawa, Ontario, Canada; School of Epidemiology and Public Health, Faculty of Medicine, University of Ottawa, Ottawa, Canada

## Abstract

The methods and results of systematic reviews should be reported in sufficient detail to allow users to assess the trustworthiness and applicability of the review findings. The Preferred Reporting Items for Systematic reviews and Meta-Analyses (PRISMA) statement was developed to facilitate transparent and complete reporting of systematic reviews and has been updated (to PRISMA 2020) to reflect recent advances in systematic review methodology and terminology. Here, we present the explanation and elaboration paper for PRISMA 2020, where we explain why reporting of each item is recommended, present bullet points that detail the reporting recommendations, and present examples from published reviews. We hope that changes to the content and structure of PRISMA 2020 will facilitate uptake of the guideline and lead to more transparent, complete, and accurate reporting of systematic reviews.

Systematic reviews are essential for healthcare providers, policy makers, and other decision makers, who would otherwise be confronted by an overwhelming volume of research on which to base their decisions. To allow decision makers to assess the trustworthiness and applicability of review findings, reports of systematic reviews should be transparent and complete. Furthermore, such reporting should allow others to replicate or update reviews. The Preferred Reporting Items for Systematic reviews and Meta-Analyses (PRISMA) statement published in 2009 (hereafter referred to as PRISMA 2009)^1^ ^2^ ^3^ ^4^ ^5^ ^6^ ^7^ ^8^ ^9^ ^10^ ^11^ ^12^ was designed to help authors prepare transparent accounts of their reviews, and its recommendations have been widely endorsed and adopted.^13^ We have updated the PRISMA 2009 statement (to PRISMA 2020) to ensure currency and relevance and to reflect advances in systematic review methodology and terminology.

**Summary points**

- The PRISMA 2020 statement includes a checklist of 27 items to guide reporting of systematic reviews
- In this article we explain why reporting of each item is recommended, present bullet points that detail the reporting recommendations, and present examples from published reviews
- We hope that uptake of the PRISMA 2020 statement will lead to more transparent, complete, and accurate reporting of systematic reviews, thus facilitating evidence based decision making

## Scope of this guideline

The PRISMA 2020 statement has been designed primarily for systematic reviews of studies that evaluate the effects of health interventions, irrespective of the design of the included studies. However, the checklist items are applicable to reports of systematic reviews evaluating other non-health-related interventions (for example, social or educational interventions), and many items are applicable to systematic reviews with objectives other than evaluating interventions (such as evaluating aetiology, prevalence, or prognosis). PRISMA 2020 is intended for use in systematic reviews that include synthesis (such as pairwise meta-analysis or other statistical synthesis methods) or do not include synthesis (for example, because only one eligible study is identified). The PRISMA 2020 items are relevant for mixed-methods systematic reviews (which include quantitative and qualitative studies), but reporting guidelines addressing the presentation and synthesis of qualitative data should also be consulted.^14^ ^15^ PRISMA 2020 can be used for original systematic reviews, updated systematic reviews, or continually updated (“living”) systematic reviews. However, for updated and living systematic reviews, there may be some additional considerations that need to be addressed. Extensions to the PRISMA 2009 statement have been developed to guide reporting of network meta-analyses,^16^ meta-analyses of individual participant data,^17^ systematic reviews of harms,^18^ systematic reviews of diagnostic test accuracy studies,^19^ and scoping reviews^20^; for these types of reviews we recommend authors report their review in accordance with the recommendations in PRISMA 2020 along with the guidance specific to the extension. Separate guidance for items that should be described in protocols of systematic reviews is available (PRISMA-P 2015 statement).^21^ ^22^

## PRISMA 2020 explanation and elaboration

PRISMA 2020 is published as a suite of three papers: a statement paper (consisting of the 27-item checklist, an expanded checklist that details reporting recommendations for each item, the PRISMA 2020 abstract checklist, and the revised flow diagram^23^); a development paper (which outlines the steps taken to update the PRISMA 2009 statement and provides rationale for modifications to the original items^24^); and this paper, the updated explanation and elaboration for PRISMA 2020. In this paper, for each item, we explain why reporting of the item is recommended and present bullet points that detail the reporting recommendations. This structure is new to PRISMA 2020 and has been adopted to facilitate implementation of the guidance.^25^ ^26^ Authors familiar with PRISMA 2020 may opt to use the standalone statement paper^23^; however, for those who are new to or unfamiliar with PRISMA 2020, we encourage use of this explanation and elaboration document. Box 1 includes a glossary of terms used throughout the PRISMA 2020 explanation and elaboration paper.

Box 1 **Glossary of terms**

*Systematic review*—A review that uses explicit, systematic methods to collate and synthesize findings of studies that address a clearly formulated question^27^
*Statistical synthesis*—The combination of quantitative results of two or more studies. This encompasses meta-analysis of effect estimates (described below) and other methods, such as combining P values, calculating the range and distribution of observed effects, and vote counting based on the direction of effect (see McKenzie and Brennan^28^ for a description of each method)
*Meta-analysis of effect estimates*—A statistical technique used to synthesize results when study effect estimates and their variances are available, yielding a quantitative summary of results^28^
*Outcome*—An event or measurement collected for participants in a study (such as quality of life, mortality)
*Result*—The combination of a point estimate (such as a mean difference, risk ratio or proportion) and a measure of its precision (such as a confidence/credible interval) for a particular outcome
*Report*—A document (paper or electronic) supplying information about a particular study. It could be a journal article, preprint, conference abstract, study register entry, clinical study report, dissertation, unpublished manuscript, government report, or any other document providing relevant information
*Record*—The title or abstract (or both) of a report indexed in a database or website (such as a title or abstract for an article indexed in Medline). Records that refer to the same report (such as the same journal article) are “duplicates”; however, records that refer to reports that are merely similar (such as a similar abstract submitted to two different conferences) should be considered unique.
*Study*—An investigation, such as a clinical trial, that includes a defined group of participants and one or more interventions and outcomes. A “study” might have multiple reports. For example, reports could include the protocol, statistical analysis plan, baseline characteristics, results for the primary outcome, results for harms, results for secondary outcomes, and results for additional mediator and moderator analyses

We use standardised language in the explanation and elaboration to indicate whether the reporting recommendations for each item (which we refer to as “elements” throughout) are essential or additional. Essential elements should be reported in the main report or as supplementary material for all systematic reviews (except for those preceded by “If…,” which should only be reported where applicable). These have been selected as essential because we consider their reporting important for users to assess the trustworthiness and applicability of a review’s findings, or their reporting would aid in reproducing the findings. Additional elements are those which are not essential but provide supplementary information that may enhance the completeness and usability of systematic review reports. The essential and additional elements are framed in terms of reporting the “presence” of a method or result (such as reporting if individuals were contacted to identify studies) rather than reporting on their absence. In some instances, however, reporting the absence of a method may be helpful (for example, “We did not contact individuals to identify studies”). We leave these decisions to the judgment of authors. Finally, although PRISMA 2020 provides a template for where information might be located, the suggested location should not be seen as prescriptive; the guiding principle is to ensure the information is reported.

Journals and publishers might impose word and section limits, and limits on the number of tables and figures allowed in the main report. In such cases, if the relevant information for some items already appears in a publicly accessible review protocol, referring to the protocol may suffice. Alternatively, placing detailed descriptions of the methods used or additional results (such as for less critical outcomes) in supplementary files is recommended. Ideally, supplementary files should be deposited to a general-purpose or institutional open-access repository that provides free and permanent access to the material (such as Open Science Framework, Dryad, figshare). A reference or link to the additional information should be included in the main report.

We sought examples of good reporting for each checklist item from published systematic reviews and present one for each item below; more examples are available in table S1 in the data supplement on bmj.com. We have edited the examples by removing all citations within them (to avoid potential confusion with the citation for each example), and we spelled out abbreviations to aid comprehension. We encourage readers to submit evidence that informs any of the recommendations in PRISMA 2020 and any examples that could be added to our bank of examples of good reporting (via the PRISMA statement website http://www.prisma-statement.org/).

## Title

### Item 1. Identify the report as a systematic review

***Explanation:*** Inclusion of “systematic review” in the title facilitates identification by potential users (patients, healthcare providers, policy makers, etc) and appropriate indexing in databases. Terms such as “review,” “literature review,” “evidence synthesis,” or “knowledge synthesis” are not recommended because they do not distinguish systematic and non-systematic approaches. We also discourage using the terms “systematic review” and “meta-analysis” interchangeably because a systematic review refers to the entire set of processes used to identify, select, and synthesise evidence, whereas meta-analysis refers only to the statistical synthesis. Furthermore, a meta-analysis can be done outside the context of a systematic review (for example, when researchers meta-analyse results from a limited set of studies that they have conducted).

#### **Essential elements**

- Identify the report as a systematic review in the title.
- Report an informative title that provides key information about the main objective or question that the review addresses (for reviews of interventions, this usually includes the population and the intervention(s) that the review addresses).

#### **Additional elements**

- Consider providing additional information in the title, such as the method of analysis used (for example, “a systematic review with meta-analysis”), the designs of included studies (for example, “a systematic review of randomised trials”), or an indication that the review is an update of an existing review or a continually updated (“living”) systematic review.

**Example of item 1 of PRISMA 2020 checklist**

“Comparison of the therapeutic effects of rivaroxaban versus warfarin in antiphospholipid syndrome: a systematic review”^167^

## Abstract

### Item 2. See the PRISMA 2020 for Abstracts checklist (box 2)

Box 2 **Items in the PRISMA 2020 for Abstracts checklist**

The PRISMA 2020 for Abstracts checklist retains the same items as those included in the PRISMA for Abstracts statement published in 2013^29^ but has been revised to make the wording consistent with the PRISMA 2020 statement and includes a new item recommending authors specify the methods used to present and synthesize results (item #6). The checklist includes the following 12 items:

1. Identify the report as a systematic review
2. Provide an explicit statement of the main objective(s) or question(s) the review addresses
3. Specify the inclusion and exclusion criteria for the review
4. Specify the information sources (such as databases, registers) used to identify studies and the date when each was last searched
5. Specify the methods used to assess risk of bias in the included studies
6. Specify the methods used to present and synthesise results
7. Give the total number of included studies and participants and summarise relevant characteristics of studies
8. Present results for main outcomes, preferably indicating the number of included studies and participants for each. If meta-analysis was done, report the summary estimate and confidence/credible interval. If comparing groups, indicate the direction of the effect (that is, which group is favoured)
9. Provide a brief summary of the limitations of the evidence included in the review (such as study risk of bias, inconsistency, and imprecision)
10. Provide a general interpretation of the results and important implications
11. Specify the primary source of funding for the review
12. Provide the register name and registration number

***Explanation:*** An abstract providing key information about the main objective(s) or question(s) that the review addresses, methods, results, and implications of the findings should help readers decide whether to access the full report.^29^ For some readers, the abstract may be all that they have access to. Therefore, it is critical that results are presented for all main outcomes for the main review objective(s) or question(s) regardless of the statistical significance, magnitude, or direction of effect. Terms presented in the abstract will be used to index the systematic review in bibliographic databases. Therefore, reporting keywords that accurately describe the review question (such as population, interventions, outcomes) is recommended.

#### **Essential elements**

- Report an abstract addressing each item in the PRISMA 2020 for Abstracts checklist (see box 2).

**Example of item 2 of PRISMA 2020 checklist**

“Title: Psychological interventions for common mental disorders in women experiencing intimate partner violence in low-income and middle-income countries: a systematic review and meta-analysis.

Background: Evidence on the effectiveness of psychological interventions for women with common mental disorders (CMDs) who also experience intimate partner violence is scarce. We aimed to test our hypothesis that exposure to intimate partner violence would reduce intervention effectiveness for CMDs in low-income and middle-income countries (LMICs).

Methods: For this systematic review and meta-analysis, we searched MEDLINE, Embase, PsycINFO, Web of Knowledge, Scopus, CINAHL, LILACS, ScieELO, Cochrane, PubMed databases, trials registries, 3ie, Google Scholar, and forward and backward citations for studies published between database inception and Aug 16, 2019. All randomised controlled trials (RCTs) of psychological interventions for CMDs in LMICs which measured intimate partner violence were included, without language or date restrictions. We approached study authors to obtain unpublished aggregate subgroup data for women who did and did not report intimate partner violence. We did separate random-effects meta-analyses for anxiety, depression, post-traumatic stress disorder (PTSD), and psychological distress outcomes. Evidence from randomised controlled trials was synthesised as differences between standardised mean differences (SMDs) for change in symptoms, comparing women who did and who did not report intimate partner violence via random-effects meta-analyses. The quality of the evidence was assessed with the Cochrane risk of bias tool. This study is registered on PROSPERO, number CRD42017078611.

Findings: Of 8122 records identified, 21 were eligible and data were available for 15 RCTs, all of which had a low to moderate risk of overall bias. Anxiety (five interventions, 728 participants) showed a greater response to intervention among women reporting intimate partner violence than among those who did not (difference in standardised mean differences [dSMD] 0.31, 95% CI 0.04 to 0.57, I^2^=49.4%). No differences in response to intervention were seen in women reporting intimate partner violence for PTSD (eight interventions, n=1436; dSMD 0.14, 95% CI −0.06 to 0.33, I^2^=42.6%), depression (12 interventions, n=2940; 0.10, −0.04 to 0.25, I^2^=49.3%), and psychological distress (four interventions, n=1591; 0.07, −0.05 to 0.18, I^2^=0.0%, p=0.681).

Interpretation: Psychological interventions treat anxiety effectively in women with current or recent intimate partner violence exposure in LMICs when delivered by appropriately trained and supervised health-care staff, even when not tailored for this population or targeting intimate partner violence directly. Future research should investigate whether adapting evidence-based psychological interventions for CMDs to address intimate partner violence enhances their acceptability, feasibility, and effectiveness in LMICs.

Funding: UK National Institute for Health Research ASSET and King's IoPPN Clinician Investigator Scholarship.”^168^

## Rationale

### Item 3. Describe the rationale for the review in the context of existing knowledge

***Explanation:*** Describing the rationale should help readers understand why the review was conducted and what the review might add to existing knowledge.

#### **Essential elements**

- Describe the current state of knowledge and its uncertainties.
- Articulate why it is important to do the review.
- If other systematic reviews addressing the same (or a largely similar) question are available, explain why the current review was considered necessary (for example, previous reviews are out of date or have discordant results; new review methods are available to address the review question; existing reviews are methodologically flawed; or the current review was commissioned to inform a guideline or policy for a particular organisation). If the review is an update or replication of a particular systematic review, indicate this and cite the previous review.
- If the review examines the effects of interventions, also briefly describe how the intervention(s) examined might work.

#### **Additional elements**

- If there is complexity in the intervention or context of its delivery, or both (such as multi-component interventions, interventions targeting the population and individual level, equity considerations^30^), consider presenting a logic model (sometimes referred to as a conceptual framework or theory of change) to visually display the hypothesised relationship between intervention components and outcomes.^31^ ^32^

**Example of item 3 of PRISMA 2020 checklist**

“To contain widespread infection and to reduce morbidity and mortality among health-care workers and others in contact with potentially infected people, jurisdictions have issued conflicting advice about physical or social distancing. Use of face masks with or without eye protection to achieve additional protection is debated in the mainstream media and by public health authorities, in particular the use of face masks for the general population; moreover, optimum use of face masks in health-care settings, which have been used for decades for infection prevention, is facing challenges amid personal protective equipment (PPE) shortages. Any recommendations about social or physical distancing, and the use of face masks, should be based on the best available evidence. Evidence has been reviewed for other respiratory viral infections, mainly seasonal influenza, but no comprehensive review is available of information on SARS-CoV-2 or related betacoronaviruses that have caused epidemics, such as severe acute respiratory syndrome (SARS) or Middle East respiratory syndrome (MERS). We, therefore, systematically reviewed the effect of physical distance, face masks, and eye protection on transmission of SARS-CoV-2, SARS-CoV, and MERS-CoV.”^169^

## Objectives

### Item 4. Provide an explicit statement of the objective(s) or question(s) the review addresses

***Explanation:*** An explicit and concise statement of the review objective(s) or question(s) will help readers understand the scope of the review and assess whether the methods used in the review (such as eligibility criteria, search methods, data items, and the comparisons used in the synthesis) adequately address the objective(s). Such statements may be written in the form of objectives (“the objectives of the review were to examine the effects of…”) or as questions (“what are the effects of…?”).^31^

#### **Essential elements**

- Provide an explicit statement of all objective(s) or question(s) the review addresses, expressed in terms of a relevant question formulation framework (see Booth et al^33^ and Munn et al^34^ for various frameworks).
- If the purpose is to evaluate the effects of interventions, use the Population, Intervention, Comparator, Outcome (PICO) framework or one of its variants to state the comparisons that will be made.

**Example of item 4 of PRISMA 2020 checklist**

“Objectives: To evaluate the benefits and harms of down‐titration (dose reduction, discontinuation, or disease activity‐guided dose tapering) of anti‐tumour necrosis factor-blocking agents (adalimumab, certolizumab pegol, etanercept, golimumab, infliximab) on disease activity, functioning, costs, safety, and radiographic damage compared with usual care in people with rheumatoid arthritis and low disease activity.”^170^

## Eligibility criteria

### Item 5. Specify the inclusion and exclusion criteria for the review and how studies were grouped for the syntheses

***Explanation:*** Specifying the criteria used to decide what evidence was eligible or ineligible in sufficient detail should enable readers to understand the scope of the review and verify inclusion decisions.^35^ The PICO framework is commonly used to structure the reporting of eligibility criteria for reviews of interventions.^36^ In addition to specifying the review PICO, the intervention, outcome, and population groups that were used in the syntheses need to be identified and defined.^37^ For example, in a review examining the effects of psychological interventions for smoking cessation in pregnancy, the authors specified intervention groups (counselling, health education, feedback, incentive-based interventions, social support, and exercise) and the defining components of each group.^38^

#### **Essential elements**

- Specify all study characteristics used to decide whether a study was eligible for inclusion in the review, that is, components described in the PICO framework or one of its variants,^33^ ^34^ and other characteristics, such as eligible study design(s) and setting(s) and minimum duration of follow-up.
- Specify eligibility criteria with regard to report characteristics, such as year of dissemination, language, and report status (for example, whether reports such as unpublished manuscripts and conference abstracts were eligible for inclusion).
- Clearly indicate if studies were ineligible because the outcomes of interest were not measured, or ineligible because the results for the outcome of interest were not reported. Reporting that studies were excluded because they had “no relevant outcome data” is ambiguous and should be avoided.^39^
- Specify any groups used in the synthesis (such as intervention, outcome, and population groups) and link these to the comparisons specified in the objectives (item #4).

#### **Additional elements**

- Consider providing rationales for any notable restrictions to study eligibility. For example, authors might explain that the review was restricted to studies published from 2000 onward because that was the year the device was first available.

**Example of item 5 of PRISMA 2020 checklist**

“Population: We included randomized controlled trials of adult (age ≥18 years) patients undergoing non-cardiac surgery, excluding organ transplantation surgery (as findings in patients who need immunosuppression may not be generalisable to others).

“Intervention: We considered all perioperative care interventions identified by the search if they were protocolised (therapies were systematically provided to patients according to pre-defined algorithm or plan) and were started and completed during the perioperative pathway (that is, during preoperative preparation for surgery, intraoperative care, or inpatient postoperative recovery). Examples of interventions that we did or did not deem perioperative in nature included long term preoperative drug treatment (not included, as not started and completed during the perioperative pathway) and perioperative physiotherapy interventions (included, as both started and completed during the perioperative pathway). We excluded studies in which the intervention was directly related to surgical technique.

Outcomes: To be included, a trial had to use a defined clinical outcome relating to postoperative pulmonary complications, such as “pneumonia” diagnosed according to the Centers for Disease Control and Prevention’s definition. Randomized controlled trials reporting solely physiological (for example, lung volumes and flow measurements) or biochemical (for example, lung inflammatory markers) outcomes are valuable but neither patient centric nor necessarily clinically relevant, and we therefore excluded them. We applied no language restrictions. Our primary outcome measure was the incidence of postoperative pulmonary complications, with postoperative pulmonary complications being defined as the composite of any of respiratory infection, respiratory failure, pleural effusion, atelectasis, or pneumothorax…Where a composite postoperative pulmonary complication was not reported, we contacted corresponding authors via email to request additional information, including primary data.”^171^

## Information sources

### Item 6. Specify all databases, registers, websites, organisations, reference lists, and other sources searched or consulted to identify studies. Specify the date when each source was last searched or consulted

***Explanation:*** Authors should provide a detailed description of the information sources, such as bibliographic databases, registers and reference lists that were searched or consulted, including the dates when each source was last searched, to allow readers to assess the completeness and currency of the systematic review, and facilitate updating.^40^ Authors should fully report the “what, when, and how” of the sources searched; the “what” and “when” are covered in item #6, and the “how” is covered in item #7. Further guidance and examples about searching can be found in PRISMA-Search, an extension to the PRISMA statement for reporting literature searches in systematic reviews.^41^

#### **Essential elements**

- Specify the date when each source (such as database, register, website, organisation) was last searched or consulted.
- If bibliographic databases were searched, specify for each database its name (such as MEDLINE, CINAHL), the interface or platform through which the database was searched (such as Ovid, EBSCOhost), and the dates of coverage (where this information is provided).
- If study registers (such as ClinicalTrials.gov), regulatory databases (such as Drugs@FDA), and other online repositories (such as SIDER Side Effect Resource) were searched, specify the name of each source and any date restrictions that were applied.
- If websites, search engines, or other online sources were browsed or searched, specify the name and URL (uniform resource locator) of each source.
- If organisations or manufacturers were contacted to identify studies, specify the name of each source.
- If individuals were contacted to identify studies, specify the types of individuals contacted (such as authors of studies included in the review or researchers with expertise in the area).
- If reference lists were examined, specify the types of references examined (such as references cited in study reports included in the systematic review, or references cited in systematic review reports on the same or a similar topic).
- If cited or citing reference searches (also called backwards and forward citation searching) were conducted, specify the bibliographic details of the reports to which citation searching was applied, the citation index or platform used (such as Web of Science), and the date the citation searching was done.
- If journals or conference proceedings were consulted, specify the names of each source, the dates covered and how they were searched (such as handsearching or browsing online).

**Example of item 6 of PRISMA 2020 checklist**

“On 21 December 2017, MAJ searched 16 health, social care, education, and legal databases, the names and date coverage of which are given in the Table 1…We also carried out a ‘snowball’ search to identify additional studies by searching the reference lists of publications eligible for full-text review and using Google Scholar to identify and screen studies citing them…On 26 April 2018, we conducted a search of Google Scholar and additional supplementary searches for publications on websites of 10 relevant organisations (including government departments, charities, think-tanks, and research institutes). Full details of these supplementary searches can be found in the Additional file. Finally, we updated the database search on 7 May 2019, and the snowball and additional searches on 10 May 2019 as detailed in the Additional file. We used the same search method, except that we narrowed the searches to 2017 onwards.”^172^

**Table 1:** The table displays for each database consulted its name (such as MEDLINE), the interface or platform through which the database was searched (such as Ovid), and the dates of coverage (reproduced from Jay et al^172^)

Database	Coverage
Ovid	
Medline and Epub Ahead of Print, In-Process and Other Non-Index Citations, Daily and Versions	1946 to present
Embase and Embase Classic	1947 to present
PsycInfo	1806 to present
Social Policy and Practice	1890s to present
Scopus	1788 to present
EBSCOhost	
British Education Index	1929 to present
Education Abstracts	1983 to present 1995 to present (books)
The Education Resources Information Center	1966 to present
Index to Legal Periodicals and Books	1979 to present
ProQuest Central	
The Education Database	1988 to present
Social Science Database	1942 to present
The Applied Social Sciences Index and Abstracts	1987 to present
The International Bibliography of the Social Sciences	1951 to present
The Sociology Database	1985 to present
Sociological Abstracts	1952 to present
Westlaw UK	1986 to present

## Search strategy

### Item 7. Present the full search strategies for all databases, registers, and websites, including any filters and limits used

***Explanation:*** Reporting the full details of all search strategies (such as the full, line by line search strategy as run in each database) should enhance the transparency of the systematic review, improve replicability, and enable a review to be more easily updated.^40^ ^42^ Presenting only one search strategy from among several hinders readers’ ability to assess how comprehensive the searchers were and does not provide them with the opportunity to detect any errors. Furthermore, making only one search strategy available limits replication or updating of the searches in the other databases, as the search strategies would need to be reconstructed through adaptation of the one(s) made available. As well as reporting the search strategies, a description of the search strategy development process can help readers judge how far the strategy is likely to have identified all studies relevant to the review’s inclusion criteria. The description of the search strategy development process might include details of the approaches used to identify keywords, synonyms, or subject indexing terms used in the search strategies, or any processes used to validate or peer review the search strategies. Empirical evidence suggests that peer review of search strategies is associated with improvements to search strategies, leading to retrieval of additional relevant records.^43^ Further guidance and examples of reporting search strategies can be found in PRISMA-Search.^41^

#### **Essential elements**

- Provide the full line by line search strategy as run in each database with a sophisticated interface (such as Ovid), or the sequence of terms that were used to search simpler interfaces, such as search engines or websites.
- Describe any limits applied to the search strategy (such as date or language) and justify these by linking back to the review’s eligibility criteria.
- If published approaches such as search filters designed to retrieve specific types of records (for example, filter for randomised trials)^44^ or search strategies from other systematic reviews, were used, cite them. If published approaches were adapted—for example, if existing search filters were amended—note the changes made.
- If natural language processing or text frequency analysis tools were used to identify or refine keywords, synonyms, or subject indexing terms to use in the search strategy,^45^ ^46^ specify the tool(s) used.
- If a tool was used to automatically translate search strings for one database to another,^47^ specify the tool used.
- If the search strategy was validated—for example, by evaluating whether it could identify a set of clearly eligible studies—report the validation process used and specify which studies were included in the validation set.^40^
- If the search strategy was peer reviewed, report the peer review process used and specify any tool used, such as the Peer Review of Electronic Search Strategies (PRESS) checklist.^48^
- If the search strategy structure adopted was not based on a PICO-style approach, describe the final conceptual structure and any explorations that were undertaken to achieve it (for example, use of a multi-faceted approach that uses a series of searches, with different combinations of concepts, to capture a complex research question, or use of a variety of different search approaches to compensate for when a specific concept is difficult to define).^40^

**Example of item 7 of PRISMA 2020 checklist**

Note: the following is an abridged version of an example presented in full in supplementary table S1 on bmj.com.

“MEDLINE(R) In-Process & Other Non-Indexed Citations and Ovid MEDLINE were searched via OvidSP. The database coverage was 1946 to present and the databases were searched on 29 August 2013.

1. Urinary Bladder, Overactive/
2. ((overactiv$ or over-activ$ or hyperactiv$ or hyper-activ$ or unstable or instability or incontinen$) adj3 bladder$).ti,ab.
3. (OAB or OABS or IOAB or IOABS).ti,ab.
4. (urge syndrome$ or urge frequenc$).ti,ab.
5. ((overactiv$ or over-activ$ or hyperactiv$ or hyper-activ$ or unstable or instability) adj3 detrusor$).ti,ab.
6. Urination Disorders/
7. exp Urinary Incontinence/
8. Urinary Bladder Diseases/
9. (urge$ adj3 incontinen$).ti,ab.
10. (urin$ adj3 (incontinen$ or leak$ or urgen$ or frequen$)).ti,ab.
11. (urin$ adj3 (disorder$ or dysfunct$)).ti,ab.
12. (detrusor$ adj3 (hyperreflexia$ or hyper-reflexia$ or hypertoni$ or hyper-toni$)).ti,ab.
13. (void$ adj3 (disorder$ or dysfunct$)).ti,ab.
14. (micturition$ adj3 (disorder$ or dysfunct$)).ti,ab.
15. exp Enuresis/
16. Nocturia/
17. (nocturia or nycturia or enuresis).ti,ab.
18. or/1-17
19. (mirabegron or betmiga$ or myrbetriq$ or betanis$ or YM-178 or YM178 or 223673-61-8 or “223673618” or MVR3JL3B2V).ti,ab,rn.
20. exp Electric Stimulation Therapy/
21. Electric Stimulation/
22. ((sacral or S3) adj3 (stimulat$ or modulat$)).ti,ab.
23. (neuromodulat$ or neuro-modulat$ or neural modulat$ or electromodulat$ or electro-modulat$ or neurostimulat$ or neuro-stimulat$ or neural stimulat$ or electrostimulat$ or electro-stimulat$).ti,ab.
24. (InterStim or SNS).ti,ab.
25. ((electric$ or nerve$1) adj3 (stimulat$ or modulat$)).ti,ab.
26. (electric$ therap$ or electrotherap$ or electro-therap$).ti,ab.
27. TENS.ti,ab.
28. exp Electrodes/
29. electrode$1.ti,ab.
30. ((implant$ or insert$) adj3 pulse generator$).ti,ab.
31. ((implant$ or insert$) adj3 (neuroprosthe$ or neuro-prosthe$ or neural prosthe$)).ti,ab.
32. PTNS.ti,ab.
33. (SANS or Stoller Afferent or urosurg$).ti,ab.
34. (evaluat$ adj3 peripheral nerve$).ti,ab.
35. exp Botulinum Toxins/
36. (botulinum$ or botox$ or onabotulinumtoxin$ or 1309378-01-5 or “1309378015”).ti,ab,rn.
37. or/19-36
38. 18 and 37
39. randomized controlled trial.pt.
40. controlled clinical trial.pt.
41. random$.ti,ab.
42. placebo.ti,ab.
43. drug therapy.fs.
44. trial.ti,ab.
45. groups.ab.
46. or/39-45
47. 38 and 46
48. animals/ not humans/
49. 47 not 48
50. limit 49 to english language

Search strategy development process: Five known relevant studies were used to identify records within databases. Candidate search terms were identified by looking at words in the titles, abstracts and subject indexing of those records. A draft search strategy was developed using those terms and additional search terms were identified from the results of that strategy. Search terms were also identified and checked using the PubMed PubReMiner word frequency analysis tool. The MEDLINE strategy makes use of the Cochrane RCT filter reported in the Cochrane Handbook v5.2. As per the eligibility criteria the strategy was limited to English language studies. The search strategy was validated by testing whether it could identify the five known relevant studies and also three further studies included in two systematic reviews identified as part of the strategy development process. All eight studies were identified by the search strategies in MEDLINE and Embase. The strategy was developed by an information specialist and the final strategies were peer reviewed by an experienced information specialist within our team. Peer review involved proofreading the syntax and spelling and overall structure, but did not make use of the PRESS checklist.”^173^

## Selection process

### Item 8. Specify the methods used to decide whether a study met the inclusion criteria of the review, including how many reviewers screened each record and each report retrieved, whether they worked independently, and, if applicable, details of automation tools used in the process

***Explanation:*** Study selection is typically a multi-stage process in which potentially eligible studies are first identified from screening titles and abstracts, then assessed through full text review and, where necessary, contact with study investigators. Increasingly, a mix of screening approaches might be applied (such as automation to eliminate records before screening or prioritise records during screening). In addition to automation, authors increasingly have access to screening decisions that are made by people independent of the author team (such as crowdsourcing) (see box 3). Authors should describe in detail the process for deciding how records retrieved by the search were considered for inclusion in the review, to enable readers to assess the potential for errors in selection.^49^ ^50^ ^51^ ^52^

Box 3 **Study selection methods**

Several approaches to selecting studies exist. Here we comment on the advantages and disadvantages of each.

*Assessment of each record by one reviewer—*Single screening is an efficient use of time and resources, but there is a higher risk of missing relevant studies^49^ ^50^ ^51^
*Assessment of records by more than one reviewer—*Double screening can vary from duplicate checking of all records (by two or more reviewers independently) to a second reviewer checking a sample only (for example, a random sample of screened records, or all excluded records). This approach may be more reliable than single screening but at the expense of increased reviewer time, given the time needed to resolve discrepancies^49^ ^50^ ^51^
*Priority screening to focus early screening effort on most relevant records—*Instead of screening records in year, title, author or random order, machine learning is used to identify relevant studies earlier in the screening process than would otherwise be the case. Priority screening is an iterative process in which the machine continually reassesses unscreened records for relevance. This approach can increase review efficiency by enabling the review team to start on subsequent steps of the review while less relevant records are still being screened. Both single and multiple reviewer assessments can be combined with priority screening^52^ ^53^
*Priority screening with the automatic elimination of less relevant records—*Once the most relevant records have been identified using priority screening, teams may choose to stop screening based on the assumption that the remaining records are unlikely to be relevant. However, there is a risk of erroneously excluding relevant studies because of uncertainty about when it is safe to stop screening; the balance between efficiency gains and risk tolerance will be review-specific^52^ ^53^
*Machine learning classifiers—*Machine learning classifiers are statistical models that use training data to rank records according to their relevance. They can be calibrated to achieve a given level of recall, thus enabling reviewers to implement screening rules, such as eliminating records or replacing double with single screening. Because the performance of classifiers is highly dependent on the data used to build them, classifiers should only be used to classify records for which they are designed^53^ ^54^
*Previous “known” assessments—*Screening decisions for records that have already been manually checked can be reused to exclude the same records from being reassessed, provided the eligibility criteria are the same. For example, groups that maintain registers of controlled trials to facilitate systematic reviews can avoid continually rescreening the same records by matching and then including/excluding those records from further consideration.
*Crowdsourcing—*Crowdsourcing involves recruiting (usually via the internet) a large group of individuals to contribute to a task or project, such as screening records. If crowdsourcing is integrated with other study selection approaches, the specific platforms used should have well established and documented agreement algorithms, and data on crowd accuracy and reliability^55^ ^56^

#### **Essential elements for systematic reviews regardless of the selection processes used**

- Report how many reviewers screened each record (title/abstract) and each report retrieved, whether multiple reviewers worked independently (that is, were unaware of each other’s decisions) at each stage of screening or not (for example, records screened by one reviewer and exclusions verified by another), and any processes used to resolve disagreements between screeners (for example, referral to a third reviewer or by consensus).
- Report any processes used to obtain or confirm relevant information from study investigators.
- If abstracts or articles required translation into another language to determine their eligibility, report how these were translated (for example, by asking a native speaker or by using software programs).

#### **Essential elements for systematic reviews using automation tools in the selection process**

- Report how automation tools were integrated within the overall study selection process; for example, whether records were excluded based solely on a machine assessment or whether machine assessments were used to double-check human decisions.
- If an externally derived machine learning classifier was applied (such as Cochrane RCT Classifier), either to eliminate records or to replace a single screener, include a reference or URL to the version used. If the classifier was used to eliminate records before screening, report the number eliminated in the PRISMA flow diagram as “Records marked as ineligible by automation tools.”
- If an internally derived machine learning classifier was used to assist with the screening process, identify the software/classifier and version, describe how it was used (such as to remove records or replace a single screener) and trained (if relevant), and what internal or external validation was done to understand the risk of missed studies or incorrect classifications. For example, authors might state that the classifier was trained on the set of records generated for the review in question (as may be the case when updating reviews) and specify which thresholds were applied to remove records.
- If machine learning algorithms were used to prioritise screening (whereby unscreened records are continually re-ordered based on screening decisions), state the software used and provide details of any screening rules applied (for example, screening stopped altogether leaving some records to be excluded based on automated assessment alone, or screening switched from double to single screening once a pre-specified number or proportion of consecutive records was eliminated).

#### **Essential elements for systematic reviews using crowdsourcing or previous “known” assessments in the selection process**

- If crowdsourcing was used to screen records, provide details of the platform used and specify how it was integrated within the overall study selection process.
- If datasets of already-screened records were used to eliminate records retrieved by the search from further consideration, briefly describe the derivation of these datasets. For example, if prior work has already determined that a given record does not meet the eligibility criteria, it can be removed without manual checking. This is the case for Cochrane’s Screen4Me service, in which an increasingly large dataset of records that are known not to represent randomised trials can be used to eliminate any matching records from further consideration.

**Example of item 8 of PRISMA 2020 checklist**

“Three researchers (AP, HB-R, FG) independently reviewed titles and abstracts of the first 100 records and discussed inconsistencies until consensus was obtained. Then, in pairs, the researchers independently screened titles and abstracts of all articles retrieved. In case of disagreement, consensus on which articles to screen full-text was reached by discussion. If necessary, the third researcher was consulted to make the final decision. Next, two researchers (AP, HB-R) independently screened full-text articles for inclusion. Again, in case of disagreement, consensus was reached on inclusion or exclusion by discussion and if necessary, the third researcher (FG) was consulted.”^174^

For examples of systematic reviews using automation tools, crowdsourcing, or previous “known” assessments in the selection process, see supplementary table S1 on bmj.com

## Data collection process

### Item 9. Specify the methods used to collect data from reports, including how many reviewers collected data from each report, whether they worked independently, any processes for obtaining or confirming data from study investigators, and, if applicable, details of automation tools used in the process

***Explanation:*** Authors should report the methods used to collect data from reports of included studies, to enable readers to assess the potential for errors in the data presented.^57^ ^58^ ^59^

#### **Essential elements**

- Report how many reviewers collected data from each report, whether multiple reviewers worked independently or not (for example, data collected by one reviewer and checked by another),^60^ and any processes used to resolve disagreements between data collectors.
- Report any processes used to obtain or confirm relevant data from study investigators (such as how they were contacted, what data were sought, and success in obtaining the necessary information).
- If any automation tools were used to collect data, report how the tool was used (such as machine learning models to extract sentences from articles relevant to the PICO characteristics),^61^ ^62^ how the tool was trained, and what internal or external validation was done to understand the risk of incorrect extractions.
- If articles required translation into another language to enable data collection, report how these articles were translated (for example, by asking a native speaker or by using software programs).^63^
- If any software was used to extract data from figures,^64^ specify the software used.
- If any decision rules were used to select data from multiple reports corresponding to a study, and any steps were taken to resolve inconsistencies across reports, report the rules and steps used.^65^

**Example of item 9 of PRISMA 2020 checklist**

“We designed a data extraction form based on that used by Lumley 2009, which two review authors (RC and TC) used to extract data from eligible studies. Extracted data were compared, with any discrepancies being resolved through discussion. RC entered data into Review Manager 5 software (Review Manager 2014), double checking this for accuracy. When information regarding any of the above was unclear, we contacted authors of the reports to provide further details.”^175^

## Data items

### Item 10a. List and define all outcomes for which data were sought. Specify whether all results that were compatible with each outcome domain in each study were sought (for example, for all measures, time points, analyses), and, if not, the methods used to decide which results to collect

***Explanation:*** Defining outcomes in systematic reviews generally involves specifying outcome domains (such as pain, quality of life, adverse events such as nausea) and the time frame of measurement (such as less than six months).^37^ Included studies may report multiple results that are eligible for inclusion within the review outcome definition.^66^ ^67^ For example, a study may report results for two measures of pain (such as the McGill Pain Questionnaire and the Brief Pain Inventory), at two time points (such as four weeks and eight weeks), all of which are compatible with a review outcome defined as “pain <6 months.” Multiple results compatible with an outcome domain in a study might also arise when study investigators report results based on multiple analysis populations (such as all participants randomised, all participants receiving a specific amount of treatment), methods for handling missing data (such as multiple imputation, last-observation-carried-forward), or methods for handling confounding (such as adjustment for different covariates).^67^ ^68^ ^69^

Reviewers might seek all results that were compatible with each outcome definition from each study or use a process to select a subset of the results.^65^ ^69^ Examples of processes to select results include selecting the outcome definition that (*a*) was most common across studies, (*b*) the review authors considered “best” according to a prespecified hierarchy (for example, which prioritises measures included in a core outcome measurement set), or (*c*) the study investigators considered most important (such as the study’s primary outcome). It is important to specify the methods that were used to select the results when multiple results were available so that users are able to judge the appropriateness of those methods and whether there is potential for bias in the selection of results.

Reviewers may make changes to the inclusion or definition of the outcome domains or to the importance given to them in the review (for example, an outcome listed as “important” in the protocol is considered “critical” in the review). Providing a rationale for the change allows readers to assess the legitimacy of the change and whether it has potential to introduce bias in the review process.^70^

#### **Essential elements**

- List and define the outcome domains and time frame of measurement for which data were sought.
- Specify whether all results that were compatible with each outcome domain in each study were sought, and, if not, what process was used to select results within eligible domains.
- If any changes were made to the inclusion or definition of the outcome domains or to the importance given to them in the review, specify the changes, along with a rationale.
- If any changes were made to the processes used to select results within eligible outcome domains, specify the changes, along with a rationale.

#### **Additional elements**

- Consider specifying which outcome domains were considered the most important for interpreting the review’s conclusions (such as “critical” versus “important” outcomes) and provide rationale for the labelling (such as “a recent core outcome set identified the outcomes labelled ‘critical’ as being the most important to patients”).

**Example of item 10a of PRISMA 2020 checklist**

Note: the following is an abridged version of an example presented in full in supplementary table S1 on bmj.com.

“Eligible outcomes were broadly categorised as follows:

- Cognitive function
  - Global cognitive function
  - Domain-specific cognitive function (especially domains that reflect specific alcohol-related neuropathologies, such as psychomotor speed and working memory)
- Clinical diagnoses of cognitive impairment
  - Mild cognitive impairment (also referred to as mild neurocognitive disorders)

Any measure of cognitive function was eligible for inclusion. The tests or diagnostic criteria used in each study should have had evidence of validity and reliability for the assessment of mild cognitive impairment, but studies were not excluded on this basis…Results could be reported as an overall test score that provides a composite measure across multiple areas of cognitive ability (i.e. global cognitive function), sub-scales that provide a measure of domain-specific cognitive function or cognitive abilities (such as processing speed, memory), or both…Studies with a minimum follow-up of 6 months were eligible, a time frame chosen to ensure that studies were designed to examine more persistent effects of alcohol consumption…No restrictions were placed on the number of points at which the outcome was measured, but the length of follow-up and number of measurement points (including a baseline measure of cognition) was considered when interpreting study findings and in deciding which outcomes were similar enough to combine for synthesis.

We anticipated that individual studies would report data for multiple cognitive outcomes. Specifically, a single study may report results:

- For multiple constructs related to cognitive function, for example, global cognitive function and cognitive ability on specific domains (e.g. memory, attention, problem-solving, language);
- Using multiple methods or tools to measure the same or similar outcome, for example reporting measures of global cognitive function using both the Mini-Mental State Examination and the Montreal Cognitive Assessment;
- At multiple time points, for example, at 1, 5, and 10 years.

Where multiple cognition outcomes were reported, we selected one outcome for inclusion in analyses and for reporting the main outcomes (e.g. for GRADEing), choosing the result that provided the most complete information for analysis. Where multiple results remained, we listed all available outcomes (without results) and asked our content expert to independently rank these based on relevance to the review question, and the validity and reliability of the measures used. Measures of global cognitive function were prioritised, followed by measures of memory, then executive function. In the circumstance where results from multiple multivariable models were presented, we extracted associations from the most fully adjusted model, except in the case where an analysis adjusted for a possible intermediary along the causal pathway (i.e. post-baseline measures of prognostic factors (e.g. smoking, drug use, hypertension)).”^176^

### Item 10b. List and define all other variables for which data were sought (such as participant and intervention characteristics, funding sources). Describe any assumptions made about any missing or unclear information

***Explanation:*** Authors should report the data and information collected from the studies so that readers can understand the type of the information sought and to inform data collection in other similar reviews. Variables of interest might include characteristics of the study (such as countries, settings, number of centres, funding sources, registration status), characteristics of the study design (such as randomised or non-randomised), characteristics of participants (such as age, sex, socioeconomic status), number of participants enrolled and included in analyses, the results (such as summary statistics, estimates of effect and measures of precision, factors adjusted for in analyses), and competing interests of study authors. For reviews of interventions, authors may also collect data on characteristics of the interventions (such as what interventions and comparators were delivered, how they were delivered, by whom, where, and for how long).

#### **Essential elements**

- List and define all other variables for which data were sought. It may be sufficient to report a brief summary of information collected if the data collection and dictionary forms are made available (for example, as additional files or deposited in a publicly available repository).
- Describe any assumptions made about any missing or unclear information from the studies. For example, in a study that includes “children and adolescents,” for which the investigators did not specify the age range, authors might assume that the oldest participants would be 18 years, based on what was observed in similar studies included in the review, and should report that assumption.
- If a tool was used to inform which data items to collect (such as the Tool for Addressing Conflicts of Interest in Trials (TACIT)^71^ ^72^ or a tool for recording intervention details^73^ ^74^ ^75^), cite the tool used.

**Example of item 10b of PRISMA 2020 checklist**

“We collected data on:

- the report: author, year, and source of publication;
- the study: sample characteristics, social demography, and definition and criteria used for depression;
- the participants: stroke sequence (first ever vs recurrent), social situation, time elapsed since stroke onset, history of psychiatric illness, current neurological status, current treatment for depression, and history of coronary artery disease;
- the research design and features: sampling mechanism, treatment assignment mechanism, adherence, non‐response, and length of follow up;
- the intervention: type, duration, dose, timing, and mode of delivery.”^177^

## Study risk of bias assessment

### Item 11. Specify the methods used to assess risk of bias in the included studies, including details of the tool(s) used, how many reviewers assessed each study and whether they worked independently, and, if applicable, details of automation tools used in the process

***Explanation:*** Users of reviews need to know the risk of bias in the included studies to appropriately interpret the evidence. Numerous tools have been developed to assess study limitations for various designs.^76^ However, many tools have been criticised because of their content (which may extend beyond assessing study limitations that have the potential to bias findings) and the way in which the items are combined (such as scales where items are combined to yield a numerical score) (see box 4).^72^ Reporting details of the selected tool enables readers to assess whether the tool focuses solely on items that have the potential to bias findings. Reporting details of how studies were assessed (such as by one or two authors) allows readers to assess the potential for errors in the assessments.^58^ Reporting how risk of bias assessments were incorporated into the analysis is addressed in Items #13e and #13f.

Box 4 **Assessment of risk of bias in studies and bias due to missing results**

**Terminology**

The terms “quality assessment” and “critical appraisal” are often used to describe the process of evaluating the methodological conduct or reporting of studies.^76^ In PRISMA 2020, we distinguish “quality” from “risk of bias” and have focused the relevant items and elaborations on the latter. Risk of bias refers to the potential for study findings to systematically deviate from the truth due to methodological flaws in the design, conduct or analysis.^72^ Quality is not well defined, but has been shown to encompass constructs beyond those that may bias the findings, including, for example, imprecision, reporting completeness, ethics, and applicability.^77^ ^78^ ^79^ In systematic reviews, focus should be given to the design, conduct, and analysis features that may lead to important bias in the findings.

**Different types of risk of bias**

In PRISMA 2020, two aspects of risk of bias are considered. The first aspect is risk of bias in the results of the individual studies included in a systematic review. Empirical evidence and theoretical considerations suggest that several features of study design are associated with larger intervention effect estimates in studies; these features include inadequate generation and concealment of a random sequence to assign participants to groups, substantial loss to follow-up of participants, and unblinded outcome assessment.^80^

The second aspect is risk of bias in the result of a synthesis (such as meta-analysis) due to missing studies or results within studies. Missing studies/results may introduce bias when the decision to publish a study/result is influenced by the observed P value or magnitude or direction of the effect.^81^ For example, studies with statistically non-significant results may not have been submitted for publication (publication bias), or particular results that were statistically non-significant may have been omitted from study reports (selective non-reporting bias).^82^ ^83^

**Tools for assessing risk of bias**

Many tools have been developed to assess the risk of bias in studies^76^ ^78^ ^79^ or bias due to missing results.^84^ Existing tools typically take the form of composite scales and domain-based tools.^78^ ^85^ Composite scales include multiple items which each have a numeric score attached, from which an overall summary score might be calculated. Domain-based tools require users to judge risk of bias within specific domains, and to record the information on which each judgment was based.^72^ ^86^ ^87^ Specifying the components/domains in the tool used in the review can help readers determine whether the tool focuses on risk of bias only or addresses other “quality” constructs. Presenting assessments for each component/domain in the tool is preferable to reporting a single “quality score” because it enables users to understand the specific components/domains that are at risk of bias in each study.

**Incorporating assessments of risk of bias in studies into the analysis**

The risk of bias in included studies should be considered in the presentation and interpretation of results of individual studies and syntheses. Different analytic strategies may be used to examine whether the risks of bias of the studies may influence the study results: (i) restricting the primary analysis to studies judged to be at low risk of bias (sensitivity analysis); (ii) stratifying studies according to risk of bias using subgroup analysis or meta-regression; or (iii) adjusting the result from each study in an attempt to remove the bias. Further details about each approach are available elsewhere.^72^

#### **Essential elements**

- Specify the tool(s) (and version) used to assess risk of bias in the included studies.
- Specify the methodological domains/components/items of the risk of bias tool(s) used.
- Report whether an overall risk of bias judgment that summarised across domains/components/items was made, and if so, what rules were used to reach an overall judgment.
- If any adaptations to an existing tool to assess risk of bias in studies were made (such as omitting or modifying items), specify the adaptations.
- If a new risk of bias tool was developed for use in the review, describe the content of the tool and make it publicly accessible.
- Report how many reviewers assessed risk of bias in each study, whether multiple reviewers worked independently (such as assessments performed by one reviewer and checked by another), and any processes used to resolve disagreements between assessors.
- Report any processes used to obtain or confirm relevant information from study investigators.
- If an automation tool was used to assess risk of bias in studies, report how the automation tool was used (such as machine learning models to extract sentences from articles relevant to risk of bias^88^), how the tool was trained, and details on the tool’s performance and internal validation.

**Example of item 11 of PRISMA 2020 checklist**

“We assessed risk of bias in the included studies using the revised Cochrane ‘Risk of bias’ tool for randomised trials (RoB 2.0) (Higgins 2016a), employing the additional guidance for cluster-randomised and cross-over trials (Eldridge 2016; Higgins 2016b). RoB 2.0 addresses five specific domains: (1) bias arising from the randomisation process; (2) bias due to deviations from intended interventions; (3) bias due to missing outcome data; (4) bias in measurement of the outcome; and (5) bias in selection of the reported result. Two review authors independently applied the tool to each included study, and recorded supporting information and justifications for judgements of risk of bias for each domain (low; high; some concerns). Any discrepancies in judgements of risk of bias or justifications for judgements were resolved by discussion to reach consensus between the two review authors, with a third review author acting as an arbiter if necessary. Following guidance given for RoB 2.0 (Section 1.3.4) (Higgins 2016a), we derived an overall summary 'Risk of bias' judgement (low; some concerns; high) for each specific outcome, whereby the overall RoB for each study was determined by the highest RoB level in any of the domains that were assessed.”^178^

## Effect measures

### Item 12. Specify for each outcome the effect measure(s) (such as risk ratio, mean difference) used in the synthesis or presentation of results

***Explanation:*** To interpret a synthesised or study result, users need to know what effect measure was used. Effect measures refer to statistical constructs that compare outcome data between two groups. For instance, a risk ratio is an example of an effect measure that might be used for dichotomous outcomes.^89^ The chosen effect measure has implications for interpretation of the findings and might affect the meta-analysis results (such as heterogeneity^90^). Authors might use one effect measure to synthesise results and then re-express the synthesised results using another effect measure. For example, for meta-analyses of standardised mean differences, authors might re-express the combined results in units of a well known measurement scale, and for meta-analyses of risk ratios or odds ratios, authors might re-express results in absolute terms (such as risk difference).^91^ Furthermore, authors need to interpret effect estimates in relation to whether the effect is of importance to decision makers. For a particular outcome and effect measure, this requires specification of thresholds (or ranges) used to interpret the size of effect (such as minimally important difference; ranges for no/trivial, small, moderate, and large effects).^91^

#### **Essential elements**

- Specify for each outcome or type of outcome (such as binary, continuous) the effect measure(s) (such as risk ratio, mean difference) used in the synthesis or presentation of results.
- State any thresholds or ranges used to interpret the size of effect (such as minimally important difference; ranges for no/trivial, small, moderate, and large effects) and the rationale for these thresholds.
- If synthesised results were re-expressed to a different effect measure, report the methods used to re-express results (such as meta-analysing risk ratios and computing an absolute risk reduction based on an assumed comparator risk).

#### **Additional elements**

- Consider providing justification for the choice of effect measure. For example, a standardised mean difference may have been chosen because multiple instruments or scales were used across studies to measure the same outcome domain (such as different instruments to assess depression).

**Example of item 12 of PRISMA 2020 checklist**

“We planned to analyse dichotomous outcomes by calculating the risk ratio (RR) of a successful outcome (i.e. improvement in relevant variables) for each trial…Because the included resilience‐training studies used different measurement scales to assess resilience and related constructs, we used standardised mean difference (SMD) effect sizes (Cohen's d) and their 95% confidence intervals (CIs) for continuous data in pair‐wise meta‐analyses.”^179^

## Synthesis methods

### Item 13a. Describe the processes used to decide which studies were eligible for each synthesis (such as tabulating the study intervention characteristics and comparing against the planned groups for each synthesis (item #5))

***Explanation:*** Before undertaking any statistical synthesis (item #13d), decisions must be made about which studies are eligible for each planned synthesis (item #5). These decisions will likely involve subjective judgments that could alter the result of a synthesis, yet the processes used and information to support the decisions are often absent from reviews. Reporting the processes (whether formal or informal) and any supporting information is recommended for transparency of the decisions made in grouping studies for synthesis. Structured approaches may involve the tabulation and coding of the main characteristics of the populations, interventions, and outcomes.^92^ For example, in a review examining the effects of psychological interventions for smoking cessation in pregnancy, the main intervention component of each study was coded as one of the following based on pre-specified criteria: counselling, health education, feedback, incentive-based interventions, social support, and exercise.^38^ This coding provided the basis for determining which studies were eligible for each planned synthesis (such as incentive-based interventions versus usual care). Similar coding processes can be applied to populations and outcomes.

#### **Essential elements**

- Describe the processes used to decide which studies were eligible for each synthesis.

**Example of item 13a of PRISMA 2020 checklist**

“Given the complexity of the interventions being investigated, we attempted to categorize the included interventions along four dimensions: (1) was housing provided to the participants as part of the intervention; (2) to what degree was the tenants’ residence in the provided housing dependent on, for example, sobriety, treatment attendance, etc.; (3) if housing was provided, was it segregated from the larger community, or scattered around the city; and (4) if case management services were provided as part of the intervention, to what degree of intensity. We created categories of interventions based on the above dimensions:

1. Case management only
2. Abstinence-contingent housing
3. Non-abstinence-contingent housing
4. Housing vouchers
5. Residential treatment with case management

Some of the interventions had multiple components (e.g. abstinence-contingent housing with case management). These interventions were categorized according to the main component (the component that the primary authors emphasized). They were also placed in separate analyses. We then organized the studies according to which comparison intervention was used (any of the above interventions, or usual services).”^180^

### Item 13b. Describe any methods required to prepare the data for presentation or synthesis, such as handling of missing summary statistics or data conversions

***Explanation:*** Authors may need to prepare the data collected from studies so that it is suitable for presentation or to be included in a synthesis. This could involve algebraic manipulation to convert reported statistics to required statistics (such as converting standard errors to standard deviations),^89^ transforming effect estimates (such as converting standardised mean differences to odds ratios^93^), or imputing missing summary data (such as missing standard deviations for continuous outcomes, intra-cluster correlations in cluster randomised trials).^94^ ^95^ ^96^ Reporting the methods required to prepare the data will allow readers to judge the appropriateness of the methods used and the assumptions made and aid in attempts to replicate the synthesis.

#### **Essential elements**

- Report any methods required to prepare the data collected from studies for presentation or synthesis, such as handling of missing summary statistics or data conversions.

**Example of item 13b of PRISMA 2020 checklist**

“We used cluster-adjusted estimates from cluster randomised controlled trials (c-RCTs) where available. If the studies had not adjusted for clustering, we attempted to adjust their standard errors using the methods described in the Cochrane Handbook for Systematic Reviews of Interventions (Higgins 2019), using an estimate of the intra-cluster correlation coefficient (ICC) derived from the trial. If the trial did not report the cluster-adjusted estimated or the ICC, we imputed an ICC from a similar study included in the review, adjusting if the nature or size of the clusters was different (e.g. households compared to classrooms). We assessed any imputed ICCs using sensitivity analysis.”^181^

### Item 13c. Describe any methods used to tabulate or visually display results of individual studies and syntheses

***Explanation:*** Presentation of study results using tabulation and visual display is important for transparency (particularly so for reviews or outcomes within reviews where a meta-analysis has not been undertaken) and facilitates the identification of patterns in the data. Tables may be used to present results from individual studies or from a synthesis (such as Summary of Findings table^97^ ^98^; see item #22). The purpose of tabulating data varies but commonly includes the complete and transparent reporting of the results or comparing the results across study characteristics.^28^ Different purposes will likely lead to different table structures. Reporting the chosen structure(s), along with details of the data presented (such as effect estimates), can aid users in understanding the basis and rationale for the structure (such as, “Table have been structured by outcome domain, within which studies are ordered from low to high risk of bias to increase the prominence of the most trustworthy evidence.”).

The principal graphical method for meta-analysis is the forest plot, which displays the effect estimates and confidence intervals of each study and often the summary estimate.^99^ ^100^ Similar to tabulation, ordering the studies in the forest plot based on study characteristics (such as by size of the effect estimate, year of publication, study weight, or overall risk of bias) rather than alphabetically (as is often done) can reveal patterns in the data.^101^ Other graphs that aim to display information about the magnitude or direction of effects might be considered when a forest plot cannot be used due to incompletely reported effect estimates (such as no measure of precision reported).^28^ ^102^ Careful choice and design of graphs is required so that they effectively and accurately represent the data.^99^

#### **Essential elements**

- Report chosen tabular structure(s) used to display results of individual studies and syntheses, along with details of the data presented.
- Report chosen graphical methods used to visually display results of individual studies and syntheses.

#### **Additional elements**

- If studies are ordered or grouped within tables or graphs based on study characteristics (such as by size of the study effect, year of publication), consider reporting the basis for the chosen ordering/grouping.
- If non-standard graphs were used, consider reporting the rationale for selecting the chosen graph.

**Example of item 13c of PRISMA 2020 checklist**

“Meta-analyses could not be undertaken due to the heterogeneity of interventions, settings, study designs and outcome measures. Albatross plots were created to provide a graphical overview of the data for interventions with more than five data points for an outcome. Albatross plots are a scatter plot of p-values against the total number of individuals in each study. Small p-values from negative associations appear at the left of the plot, small p-values from positive associations at the right, and studies with null results towards the middle. The plot allows p-values to be interpreted in the context of the study sample size; effect contours show a standardised effect size (expressed as relative risk—RR) for a given p-value and study size, providing an indication of the overall magnitude of any association. We estimated an overall magnitude of association from these contours, but this should be interpreted cautiously.”^182^

### Item 13d. Describe any methods used to synthesise results and provide a rationale for the choice(s). If meta-analysis was performed, describe the model(s), method(s) to identify the presence and extent of statistical heterogeneity, and software package(s) used

***Explanation:*** Various statistical methods are available to synthesise results, the most common of which is meta-analysis of effect estimates (see box 5). Meta-analysis is used to synthesise effect estimates across studies, yielding a summary estimate. Different meta-analysis models are available, with the random-effects and fixed-effect models being in widespread use. Model choice can importantly affect the summary estimate and its confidence interval; hence the rationale for the selected model should be provided (see box 5). For random-effects models, many methods are available, and their performance has been shown to differ depending on the characteristics of the meta-analysis (such as the number and size of the included studies^113^ ^114^).

Box 5 **Meta-analysis and its extensions**

Meta-analysis is a statistical technique used to synthesise results when study effect estimates and their variances are available, yielding a quantitative summary of results.^103^ The method facilitates interpretation that would otherwise be difficult to achieve if, for example, a narrative summary of each result was presented, particularly as the number of studies increases. Furthermore, meta-analysis increases the chance of detecting a clinically important effect as statistically significant, if it exists, and increases the precision of the estimated effect.^104^

**Meta-analysis models and methods**

The summary estimate is a weighted average of the study effect estimates, where the study weights are determined primarily by the meta-analysis model. The two most common meta-analysis models are the “fixed-effect” and “random-effects” models.^103^ The assumption underlying the fixed-effect model is that there is one true (common) intervention effect and that the observed differences in results across studies reflect random variation only. This model is sometimes referred to as the “common-effects” or “equal-effects” model.^103^ A fixed-effect model can also be interpreted under a different assumption, that the true intervention effects are different and unrelated. This model is referred to as the “fixed-effects” model.^105^ The random-effects model assumes that there is not one true intervention effect but, rather, a distribution of true intervention effects and that the observed differences in results across studies reflect real differences in the effects of an intervention.^104^ The random-effects and fixed-effects models are similar in that they assume the true intervention effects are different, but they differ in that the random-effects model assumes the effects are related through a distribution, whereas the fixed-effects model does not make this assumption.

Many considerations may influence an author’s choice of meta-analysis model. For example, their choice may be based on the clinical and methodological diversity of the included studies and the expectation that the underlying intervention effects will differ (potentially leading to selection of a random-effects model) or concern about small-study effects (the tendency for smaller studies to show different effects to larger ones,^106^ potentially leading to fitting of both a random-effects and fixed-effect model). Sometimes authors select a model based on the heterogeneity statistics observed (for example, switch from a fixed-effect to a random-effects model if the I^2^ statistic was >50%).^107^ However, this practice is strongly discouraged.

There are different methods available to assign weights in fixed-effect or random-effects meta-analyses (such as Mantel-Haenszel, inverse-variance).^103^ For random-effects meta-analyses, there are also different ways to estimate the between-study variance (such as DerSimonian and Laird, restricted maximum likelihood (REML)) and calculate the confidence interval for the summary effect (such as Wald-type confidence interval, Hartung-Knapp-Sidik-Jonkman^108^). Readers are referred to Deeks et al^103^ for further information on how to select a particular meta-analysis model and method.

**Subgroup analyses, meta-regression, and sensitivity analyses**

Extensions to meta-analysis, including subgroup analysis and meta-regression, are available to explore causes of variation of results across studies (that is, statistical heterogeneity).^103^ Subgroup analyses involve splitting studies or participant data into subgroups and comparing the effects of the subgroups. Meta-regression is an extension of subgroup analysis that allows for the effect of continuous and categorical variables to be investigated.^109^ Authors might use either type of analysis to explore, for example, whether the intervention effect estimate varied with different participant characteristics (such as mild versus severe disease) or intervention characteristics (such as high versus low dose of a drug).

Sensitivity analyses are undertaken to examine the robustness of findings to decisions made during the review process. This involves repeating an analysis but using different decisions from those originally made and informally comparing the findings.^103^ For example, sensitivity analyses might have been done to examine the impact on the meta-analysis of including results from conference abstracts that have never been published in full, including studies where most (but not all) participants were in a particular age range, including studies at high risk of bias, or using a fixed-effect versus random-effects meta-analysis model.

Sensitivity analyses differ from subgroup analyses. Sensitivity analyses consist of making informal comparisons between different ways of estimating the same effect, whereas subgroup analyses consist of formally undertaking a statistical comparison across the subgroups.^103^

**Extensions to meta-analysis that model or account for dependency**

In most meta-analyses, effect estimates from independent studies are combined. Standard meta-analysis methods are appropriate for this situation, since an underlying assumption is that the effect estimates are independent. However, standard meta-analysis methods are not appropriate when the effect estimates are correlated. Correlated effect estimates arise when multiple effect estimates from a single study are calculated using some or all of the same participants and are included in the same meta-analysis. For example, where multiple effect estimates from a multi-arm trial are included in the same meta-analysis, or effect estimates for multiple outcomes from the same study are included. For this situation, a range of methods are available that appropriately model or account for the dependency of the effect estimates. These methods include multivariate meta-analysis,^110^ multilevel models,^111^ or robust variance estimation.^112^ See Lopez-Lopez for further discussion.^69^

When study data are not amenable to meta-analysis of effect estimates, alternative statistical synthesis methods (such as calculating the median effect across studies, combining P values) or structured summaries might be used.^28^ ^115^ Additional guidance for reporting alternative statistical synthesis methods is available (see Synthesis Without Meta-analysis (SWiM) reporting guideline^116^).

Regardless of the chosen synthesis method(s), authors should provide sufficient detail such that readers are able to assess the appropriateness of the selected methods and could reproduce the reported results (with access to the data).

#### **Essential elements**

- If statistical synthesis methods were used, reference the software, packages, and version numbers used to implement synthesis methods (such as *metan* in Stata 16,^117^ metafor (version 2.1-0) in R^118^).
- If it was not possible to conduct a meta-analysis, describe and justify the synthesis methods (such as combining P values was used because no or minimal information beyond P values and direction of effect was reported in the studies) or summary approach used.
- If meta-analysis was done, specify:
  - the meta-analysis model (fixed-effect, fixed-effects, or random-effects) and provide rationale for the selected model.
  - the method used (such as Mantel-Haenszel, inverse-variance).^103^
  - any methods used to identify or quantify statistical heterogeneity (such as visual inspection of results, a formal statistical test for heterogeneity,^103^ heterogeneity variance (τ^2^), inconsistency (such as I^2^ 119), and prediction intervals^120^).
- If a random-effects meta-analysis model was used, specify:
  - the between-study (heterogeneity) variance estimator used (such as DerSimonian and Laird, restricted maximum likelihood (REML)).
  - the method used to calculate the confidence interval for the summary effect (such as Wald-type confidence interval, Hartung-Knapp-Sidik-Jonkman^108^).
- If a Bayesian approach to meta-analysis was used, describe the prior distributions about quantities of interest (such as intervention effect being analysed, amount of heterogeneity in results across studies).^103^
- If multiple effect estimates from a study were included in a meta-analysis (as may arise, for example, when a study reports multiple outcomes eligible for inclusion in a particular meta-analysis), describe the method(s) used to model or account for the statistical dependency (such as multivariate meta-analysis, multilevel models, or robust variance estimation).^37^ ^69^
- If a planned synthesis was not considered possible or appropriate, report this and the reason for that decision.

#### **Additional elements**

- If a random-effects meta-analysis model was used, consider specifying other details about the methods used, such as the method for calculating confidence limits for the heterogeneity variance.

**Examples of item 13d of PRISMA 2020 checklist**

**Example 1: meta-analysis**

“As the effects of functional appliance treatment were deemed to be highly variable according to patient age, sex, individual maturation of the maxillofacial structures, and appliance characteristics, a random-effects model was chosen to calculate the average distribution of treatment effects that can be expected. A restricted maximum likelihood random-effects variance estimator was used instead of the older DerSimonian-Laird one, following recent guidance. Random-effects 95% prediction intervals were to be calculated for meta-analyses with at least three studies to aid in their interpretation by quantifying expected treatment effects in a future clinical setting. The extent and impact of between-study heterogeneity were assessed by inspecting the forest plots and by calculating the tau-squared and the I-squared statistics, respectively. The 95% CIs (uncertainty intervals) around tau-squared and the I-squared were calculated to judge our confidence about these metrics. We arbitrarily adopted the I-squared thresholds of >75% to be considered as signs of considerable heterogeneity, but we also judged the evidence for this heterogeneity (through the uncertainty intervals) and the localization on the forest plot…All analyses were run in Stata SE 14.0 (StataCorp, College Station, TX) by one author.”^183^

**Example 2: calculating the median effect across studies**

“We based our primary analyses upon consideration of dichotomous process adherence measures (for example, the proportion of patients managed according to evidence-based recommendations). In order to provide a quantitative assessment of the effects associated with reminders without resorting to numerous assumptions or conveying a misleading degree of confidence in the results, we used the median improvement in dichotomous process adherence measures across studies…With each study represented by a single median outcome, we calculated the median effect size and interquartile range across all included studies for that comparison.”^184^

### Item 13e. Describe any methods used to explore possible causes of heterogeneity among study results (such as subgroup analysis, meta-regression)

***Explanation:*** If authors used methods to explore possible causes of variation of results across studies (that is, statistical heterogeneity) such as subgroup analysis or meta-regression (see box 5), they should provide sufficient details so that readers are able to assess the appropriateness of the selected methods and could reproduce the reported results (with access to the data). Such methods might be used to explore whether, for example, participant or intervention characteristics or risk of bias of the included studies explain variation in results.

#### **Essential elements**

- If methods were used to explore possible causes of statistical heterogeneity, specify the method used (such as subgroup analysis, meta-regression).
- If subgroup analysis or meta-regression was performed, specify for each:
  - which factors were explored, levels of those factors, and which direction of effect modification was expected and why (where possible).
  - whether analyses were conducted using study-level variables (where each study is included in one subgroup only), within-study contrasts (where data on subsets of participants within a study are available, allowing the study to be included in more than one subgroup), or some combination of the above.^121^
  - how subgroup effects were compared (such as statistical test for interaction for subgroup analyses^103^).
- If other methods were used to explore heterogeneity because data were not amenable to meta-analysis of effect estimates, describe the methods used (such as structuring tables to examine variation in results across studies based on subpopulation, key intervention components, or contextual factors) along with the factors and levels.^28^ ^116^
- If any analyses used to explore heterogeneity were not pre-specified, identify them as such.

**Example of item 13e of PRISMA 2020 checklist**

“Given a sufficient number of trials, we used unadjusted and adjusted mixed-effects meta-regression analyses to assess whether variation among studies in smoking cessation effect size was moderated by tailoring of the intervention for disadvantaged groups. The resulting regression coefficient indicates how the outcome variable (log risk ratio (RR) for smoking cessation) changes when interventions take a socioeconomic-position-tailored versus non-socioeconomic-tailored approach. A statistically significant (p<0.05) coefficient indicates that there is a linear association between the effect estimate for smoking cessation and the explanatory variable. More moderators (study-level variables) can be included in the model, which might account for part of the heterogeneity in the true effects. We pre-planned an adjusted model to include important study covariates related to the intensity and delivery of the intervention (number of sessions delivered (above median vs below median), whether interventions involved a trained smoking cessation specialist (yes vs no), and use of pharmacotherapy in the intervention group (yes vs no). These covariates were included a priori as potential confounders given that programmes tailored to socioeconomic position might include more intervention sessions or components or be delivered by different professionals with varying experience. The regression coefficient estimates how the intervention effect in the socioeconomic-position-tailored subgroup differs from the reference group of non-socioeconomic-position-tailored interventions.”^185^

### Item 13f. Describe any sensitivity analyses conducted to assess robustness of the synthesised results

***Explanation:*** If authors performed sensitivity analyses to assess robustness of the synthesised results to decisions made during the review process (see box 5), they should provide sufficient details so that readers are able to assess the appropriateness of the analyses and could reproduce the reported results (with access to the data). Ideally, sensitivity analyses should be pre-specified in the protocol, but unexpected issues may emerge during the review process that necessitate their use.

#### **Essential elements**

- If sensitivity analyses were performed, provide details of each analysis (such as removal of studies at high risk of bias, use of an alternative meta-analysis model).
- If any sensitivity analyses were not pre-specified, identify them as such.

**Example of item 13f of PRISMA 2020 checklist**

“We conducted sensitivity meta-analyses restricted to trials with recent publication (2000 or later); overall low risk of bias (low risk of bias in all seven criteria); and enrolment of generally healthy women (rather than those with a specific clinical diagnosis). To incorporate trials with zero events in both intervention and control arms (which are automatically dropped from analyses of pooled relative risks), we also did sensitivity analyses for dichotomous outcomes in which we added a continuity correction of 0.5 to zero cells.”^186^

## Reporting bias assessment

### Item 14. Describe any methods used to assess risk of bias due to missing results in a synthesis (arising from reporting biases)

***Explanation:*** The validity of a synthesis may be threatened when the available results differ systematically from the missing results. This is known as “bias due to missing results” and arises from “reporting biases” such as selective non-publication and selective non-reporting of results (see box 4).^81^ Direct methods for assessing the risk of bias due to missing results include comparing outcomes and analyses pre-specified in study registers, protocols, and statistical analysis plans with results that were available in study reports. Statistical and graphical methods exist to assess whether the observed data suggest potential for missing results (such as contour enhanced funnel plots, Egger’s test) and how robust the synthesis is to different assumptions about the nature of potentially missing results (such as selection models).^84^ ^122^ ^123^ ^124^ Tools (such as checklists, scales, or domain-based tools) that prompt users to consider some or all of these approaches are available.^81^ ^84^ Therefore, reporting methods (tools, graphical, statistical, or other) used to assess risk of bias due to missing results is recommended so that readers are able to assess how appropriate the methods were. The process by which assessments were conducted should also be reported to enable readers to assess the potential for errors and facilitate replicability.

#### **Essential elements**

- Specify the methods (tool, graphical, statistical, or other) used to assess the risk of bias due to missing results in a synthesis (arising from reporting biases).
- If risk of bias due to missing results was assessed using an existing tool, specify the methodological components/domains/items of the tool, and the process used to reach a judgment of overall risk of bias.
- If any adaptations to an existing tool to assess risk of bias due to missing results were made (such as omitting or modifying items), specify the adaptations.
- If a new tool to assess risk of bias due to missing results was developed for use in the review, describe the content of the tool and make it publicly accessible.
- Report how many reviewers assessed risk of bias due to missing results in a synthesis, whether multiple reviewers worked independently, and any processes used to resolve disagreements between assessors.
- Report any processes used to obtain or confirm relevant information from study investigators.
- If an automation tool was used to assess risk of bias due to missing results, report how the tool was used, how the tool was trained, and details on the tool’s performance and internal validation.

**Example of item 14 of PRISMA 2020 checklist**

“To assess small-study effects, we planned to generate funnel plots for meta-analyses including at least 10 trials of varying size. If asymmetry in the funnel plot was detected, we planned to review the characteristics of the trials to assess whether the asymmetry was likely due to publication bias or other factors such as methodological or clinical heterogeneity of the trials. To assess outcome reporting bias, we compared the outcomes specified in trial protocols with the outcomes reported in the corresponding trial publications; if trial protocols were unavailable, we compared the outcomes reported in the methods and results sections of the trial publications.”^187^

## Certainty assessment

### Item 15. Describe any methods used to assess certainty (or confidence) in the body of evidence for an outcome

***Explanation:*** Authors typically use some criteria to decide how certain (or confident) they are in the body of evidence for each important outcome. Common factors considered include precision of the effect estimate (or sample size), consistency of findings across studies, study design limitations and missing results (risk of bias), and how directly the studies address the question. Tools and frameworks can be used to provide a systematic, explicit approach to assessing these factors and provide a common approach and terminology for communicating certainty.^125^ ^126^ ^127^ ^128^ For example, using the GRADE approach, authors will first apply criteria to assess each GRADE domain (imprecision, inconsistency, risk of bias, and so forth) and then make an overall judgment of whether the evidence supporting a result is of high, moderate, low, or very low certainty. Reporting the factors considered and the criteria used to assess each factor enables readers to determine which factors fed into reviewers’ assessment of certainty. Reporting the process by which assessments were conducted enables readers to assess the potential for errors and facilitates replication.

#### **Essential elements**

- Specify the tool or system (and version) used to assess certainty in the body of evidence.
- Report the factors considered (such as precision of the effect estimate, consistency of findings across studies) and the criteria used to assess each factor when assessing certainty in the body of evidence.
- Describe the decision rules used to arrive at an overall judgment of the level of certainty (such as high, moderate, low, very low), together with the intended interpretation (or definition) of each level of certainty.^125^
- If applicable, report any review-specific considerations for assessing certainty, such as thresholds used to assess imprecision and ranges of magnitude of effect that might be considered trivial, moderate or large, and the rationale for these thresholds and ranges (item #12).^129^
- If any adaptations to an existing tool or system to assess certainty were made, specify the adaptations in sufficient detail that the approach is replicable.
- Report how many reviewers assessed the certainty of evidence, whether multiple reviewers worked independently, and any processes used to resolve disagreements between assessors.
- Report any processes used to obtain or confirm relevant information from investigators.
- If an automation tool was used to support the assessment of certainty, report how the automation tool was used, how the tool was trained, and details on the tool’s performance and internal validation.
- Describe methods for reporting the results of assessments of certainty, such as the use of Summary of Findings tables (see item #22).
- If standard phrases that incorporate the certainty of evidence were used (such as “hip protectors probably reduce the risk of hip fracture slightly”),^130^ report the intended interpretation of each phrase and the reference for the source guidance.

Where a published system is adhered to, it may be sufficient to briefly describe the factors considered and the decision rules for reaching an overall judgment and reference the source guidance for full details of assessment criteria.

**Example of item 15 of PRISMA 2020 checklist**

“Two people (AM, JS) independently assessed the certainty of the evidence. We used the five GRADE considerations (study limitations, consistency of effect, imprecision, indirectness, and publication bias) to assess the certainty of the body of evidence as it related to the studies that contributed data to the meta-analyses for the prespecified outcomes. We assessed the certainty of evidence as high, moderate, low, or very low. We considered the following criteria for upgrading the certainty of evidence, if appropriate: large effect, dose-response gradient, and plausible confounding effect. We used the methods and recommendations described in sections 8.5 and 8.7, and chapters 11 and 12, of the Cochrane Handbook for Systematic Reviews of Interventions. We used GRADEpro GDT software to prepare the 'Summary of findings' tables (GRADEpro GDT 2015). We justified all decisions to down- or up-grade the certainty of studies using footnotes, and we provided comments to aid the reader’s understanding of the results where necessary.”^188^

## Study selection

### Item 16a. Describe the results of the search and selection process, from the number of records identified in the search to the number of studies included in the review, ideally using a flow diagram (see fig 1)

**Fig 1:**
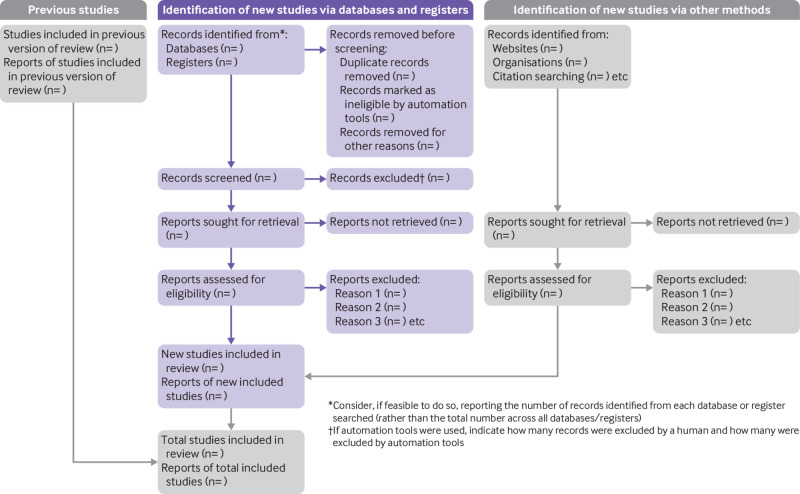
PRISMA 2020 flow diagram template for systematic reviews (adapted from flow diagrams proposed by Boers^131^ and Mayo-Wilson et al.^65^ and Stovold et al.^132^). The boxes in grey should only be completed if applicable; otherwise they should be removed from the flow diagram. Note that a “report” could be a journal article, preprint, conference abstract, study register entry, clinical study report, dissertation, unpublished manuscript, government report or any other document providing relevant information.

***Explanation:*** Review authors should report, ideally with a flow diagram (see fig 1), the results of the search and selection process so that readers can understand the flow of retrieved records through to inclusion in the review. Such information is useful for future systematic review teams seeking to estimate resource requirements and for information specialists in evaluating their searches.^133^ ^134^ Specifying the number of records yielded per database will make it easier for others to assess whether they have successfully replicated a search. The flow diagram in figure 1 provides a template of the flow of records through the review separated by source, although other layouts may be preferable depending on the information sources consulted.^65^

#### **Essential elements**

- Report, ideally using a flow diagram, the number of: records identified; records excluded before screening (for example, because they were duplicates or deemed ineligible by machine classifiers); records screened; records excluded after screening titles or titles and abstracts; reports retrieved for detailed evaluation; potentially eligible reports that were not retrievable; retrieved reports that did not meet inclusion criteria and the primary reasons for exclusion (such as ineligible study design, ineligible population); and the number of studies and reports included in the review. If applicable, authors should also report the number of ongoing studies and associated reports identified.
- If the review is an update of a previous review, report results of the search and selection process for the current review and specify the number of studies included in the previous review. An additional box could be added to the flow diagram indicating the number of studies included in the previous review (see fig 1).^132^
- If applicable, indicate in the PRISMA flow diagram how many records were excluded by a human and how many by automation tools.

**Example of item 16a of PRISMA 2020 checklist**

“We found 1,333 records in databases searching. After duplicates removal, we screened 1,092 records, from which we reviewed 34 full-text documents, and finally included six papers [each cited]. Later, we searched documents that cited any of the initially included studies as well as the references of the initially included studies. However, no extra articles that fulfilled inclusion criteria were found in these searches (a flow diagram is available at https://doi.org/10.1371/journal.pone.0233220).”^189^

### Item 16b. Cite studies that might appear to meet the inclusion criteria, but which were excluded, and explain why they were excluded

***Explanation:*** Identifying the excluded records allows readers to make an assessment of the validity and applicability of the systematic review.^40^ ^135^ At a minimum, a list of studies that might appear to meet the inclusion criteria but which were excluded, with citation and a reason for exclusion, should be reported. This would include studies meeting most inclusion criteria (such as those with appropriate intervention and population but an ineligible control or study design). It is also useful to list studies that were potentially relevant but for which the full text or data essential to inform eligibility were not accessible. This information can be reported in the text or as a list/table in the report or in an online supplement. Potentially contentious exclusions should be clearly stated in the report.

#### **Essential elements**

- Cite studies that might appear to meet the inclusion criteria, but which were excluded, and explain why they were excluded.

**Example of item 16b of PRISMA 2020 checklist**

“We excluded seven studies from our review (Bosiers 2015; ConSeQuent; DEBATE‐ISR; EXCITE ISR; NCT00481780; NCT02832024; RELINE), and we listed reasons for exclusion in the Characteristics of excluded studies tables. We excluded studies because they compared stenting in Bosiers 2015 and RELINE, laser atherectomy in EXCITE ISR, or cutting balloon angioplasty in NCT00481780 versus uncoated balloon angioplasty for in‐stent restenosis. The ConSeQuent trial compared DEB versus uncoated balloon angioplasty for native vessel restenosis rather than in‐stent restenosis. The DEBATE‐ISR study compared a prospective cohort of patients receiving DEB therapy for in‐stent restenosis against a historical cohort of diabetic patients. Finally, the NCT02832024 study compared stent deployment versus atherectomy versus uncoated balloon angioplasty alone for in‐stent restenosis.”^190^

## Study characteristics

### Item 17. Cite each included study and present its characteristics

***Explanation:*** Reporting the details of the included studies allows readers to understand the characteristics of studies that have addressed the review question(s) and is therefore important for understanding the applicability of the review. Characteristics of interest might include study design features, characteristics of participants, how outcomes were ascertained (such as smoking cessation self reported or biochemically validated, or specific harms systematically assessed or reported by participants as they emerged), funding source, and competing interests of study authors. Presenting the key characteristics of each study in a table or figure can facilitate comparison of characteristics across the studies.^92^ Citing each study enables retrieval of relevant reports if desired.

For systematic reviews of interventions, presenting an additional table that summarises the intervention details for each study (such as using the template based on the Template for Intervention Description and Replication (TIDieR)^73^) has several benefits. An intervention summary table helps readers compare the characteristics of the interventions and consider those that may be feasible for implementation in their setting; highlights missing or unavailable details; shows which studies did not specify certain characteristics as part of the intervention; and highlights characteristics that have not been investigated in existing studies.^73^ ^75^

#### **Essential elements**

- Cite each included study.
- Present the key characteristics of each study in a table or figure (considering a format that will facilitate comparison of characteristics across the studies).

#### **Additional elements**

- If the review examines the effects of interventions, consider presenting an additional table that summarises the intervention details for each study.

**Example of item 17 of PRISMA 2020 checklist**

In a review examining the association between aspirin use and fracture risk, the authors included a table presenting for each included study the citation, study design, country, sample size, setting, mean age, percentage of females, number of years follow-up, exposure details, and outcomes assessed (table 2).^191^

**Table 2:** The table displays for each included study the citation, study design, country, sample size, setting, mean age, percentage of females, number of years follow-up, exposure details and outcomes assessed. Reproduced from Barker et al.^191^

Study ID		Population						Exposure to aspirin		Outcomes	
Author (year)	Study design	Country	Sample size	Source of participants	Age, mean	Female, %	Follow-up (years)	Identification	Dose	Fracture	Bone mineral density
Bauer (1996)	Cohort	USA	7786	Community	73.1	100	1.6	Self-report	1–4 times/week	✓	✓
					74.1				5–7 times/week		
Bleicher (2011)	Cross-sectional	Australia	1705	Community	77.0	0	–	Medication verified in clinic	NR	–	✓
Bonten (2017)	Cross-sectional	Netherlands	854	Community	59.0	34	–	Medication verified in clinic	30–125 mg/day	✓	✓
Carbone (2003)	Cross-sectional	USA	2853	Community	73.6	50	–	Medication verified in clinic	328 mg/day	✓	✓
Chuang (2016)	Case-control	Taiwan	555	Community	74.0	61	5	Prescription history	106 mg	✓	–
Dobnig (2007)	Cohort	Austria	1664	Nursing homes	–	100	2	Not reported	Not reported	✓	–
Hill (2008)	Cross-sectional	Trinidad and Tobago	340	Community	63.9	100	–	Medication verified in clinic	≥3 times/week	–	✓
Hill (2008)	Cross-sectional	Trinidad and Tobago	2501	Community	56.3	0	–	Self-report	NR	–	✓
Lane (1997)	Cross-sectional	USA	499	Community	73.6	100	–	Self-report	5–7 days/week	–	✓
Vestergaard (2006, 2012)	Case-control	Denmark	498 617	Community	43.4	52	1	Prescription history	≤150 mg/day	✓	–
Vestergaard (2012)	Cohort	Denmark	2016	Community	50.8	100	10	Self-report	325 mg/day	✓	✓

## Risk of bias in studies

### Item 18. Present assessments of risk of bias for each included study

***Explanation:*** For readers to understand the internal validity of a systematic review’s results, they need to know the risk of bias in results of each included study. Reporting only summary data (such as “two of eight studies successfully blinded participants”) is inadequate because it fails to inform readers which studies had each particular methodological shortcoming. A more informative approach is to present tables or figures indicating for each study the risk of bias in each domain/component/item assessed (such as blinding of outcome assessors, missing outcome data), so that users can understand what factors led to the overall study-level risk of bias judgment.^72^ ^136^

#### **Essential elements**

- Present tables or figures indicating for each study the risk of bias in each domain/component/item assessed and overall study-level risk of bias.
- Present justification for each risk of bias judgment—for example, in the form of relevant quotations from reports of included studies.

#### **Additional elements**

- If assessments of risk of bias were done for specific outcomes or results in each study, consider displaying risk of bias judgments on a forest plot, next to the study results, so that the limitations of studies contributing to a particular meta-analysis are evident (see Sterne et al^86^ for an example forest plot).

**Example of item 18 of PRISMA 2020 checklist**

“We used the RoB 2.0 tool to assess risk of bias for each of the included studies. A summary of these assessments is provided in table 3. In terms of overall risk of bias, there were concerns about risk of bias for the majority of studies (20/24), with two of these assessed as at high risk of bias (Musher‐Eizenman 2010; Wansink 2013a). A text summary is provided below for each of the six individual components of the ‘Risk of bias’ assessment. Justifications for assessments are available at the following (https://dx.doi.org/10.6084/m9.figshare.9159824).”^178^

**Table 3:** The table displays for each included study the risk-of-bias judgment for each of six domains of bias, and for the overall risk of bias in two results (selection of a product, consumption of a product); the following is an abridged version of the table presented in the review. Reproduced from Hollands et al.^178^

Study	Bias arising from the randomisation process	Bias arising from the timing of identification and recruitment of individual participants in relation to timing of randomisation (CRCT only)	Bias due to deviations from intended interventions	Bias due to missing outcome data	Bias in measurement of the outcome	Bias in selection of the reported result	Overall risk of bias (selection of a product)	Overall risk of bias (consumption of a product)
Fiske 2004	Some concerns	Low risk	Low risk	Low risk	Low risk	Low risk	Some concerns	Not applicable
Foster 2014	Low risk	Low risk	Low risk	Low risk	Low risk	Low risk	Low risk	Not applicable
Kocken 2012	Some concerns	Low risk	Low risk	Low risk	Low risk	Low risk	Some concerns	Not applicable
Pechey 2019	Some concerns	Not applicable	Low risk	Low risk	Low risk	Low risk	Some concerns	Not applicable
Roe 2013	Some concerns	Not applicable	Low risk	Low risk	Low risk	Low risk	Some concerns	Some concerns
Stubbs 2001	Some concerns	Not applicable	Low risk	Low risk	Low risk	Low risk	Not applicable	Some concerns

CRCT: cluster-randomised controlled trials. Justifications for assessments are available at the following (https://dx.doi.org/10.6084/m9.figshare.9159824).

## Results of individual studies

### Item 19. For all outcomes, present for each study (*a*) summary statistics for each group (where appropriate) and (*b*) an effect estimate and its precision (such as confidence/credible interval), ideally using structured tables or plots

***Explanation:*** Presenting data from individual studies facilitates understanding of each study’s contribution to the findings and reuse of the data by others seeking to perform additional analyses or perform an update of the review. There are different ways of presenting results of individual studies (such as table, forest plot).^28^ ^115^ Visual display of results supports interpretation by readers, while tabulation of the results makes it easier for others to reuse the data.

Displaying summary statistics by group is helpful, because it allows an assessment of the severity of the problem in the studies (such as level of depression symptoms), which is not available from between-group results (that is, effect estimates).^137^ However, there are some scenarios where presentation of simple summary statistics for each group may be misleading. For example, in the case of cluster-randomised designs, the observed number of events and sample size in each group does not reflect the effective sample size (that is, the sample size adjusted for correlation among observations). However, providing the estimated proportion of events (or another summary statistic) per group will be helpful.^138^ The effect estimates from models that appropriately adjust for clustering (and other design features) should be reported and included in the meta-analysis in such instances.

#### **Essential elements**

- For all outcomes, irrespective of whether statistical synthesis was undertaken, present for each study summary statistics for each group (where appropriate). For dichotomous outcomes, report the number of participants with and without the events for each group; or the number with the event and the total for each group (such as 12/45). For continuous outcomes, report the mean, standard deviation, and sample size of each group.
- For all outcomes, irrespective of whether statistical synthesis was undertaken, present for each study an effect estimate and its precision (such as standard error or 95% confidence/credible interval). For example, for time-to-event outcomes, present a hazard ratio and its confidence interval.
- If study-level data are presented visually or reported in the text (or both), also present a tabular display of the results.
- If results were obtained from multiple sources (such as journal article, study register entry, clinical study report, correspondence with authors), report the source of the data. This need not be overly burdensome. For example, a statement indicating that, unless otherwise specified, all data came from the primary reference for each included study would suffice. Alternatively, this could be achieved by, for example, presenting the origin of each data point in footnotes, in a column of the data table, or as a hyperlink to relevant text highlighted in reports (such as using SRDR Data Abstraction Assistant^139^).
- If applicable, indicate which results were not reported directly and had to be computed or estimated from other information (see item #13b).

**Example of item 19 of PRISMA 2020 checklist**

**Fig 2:**
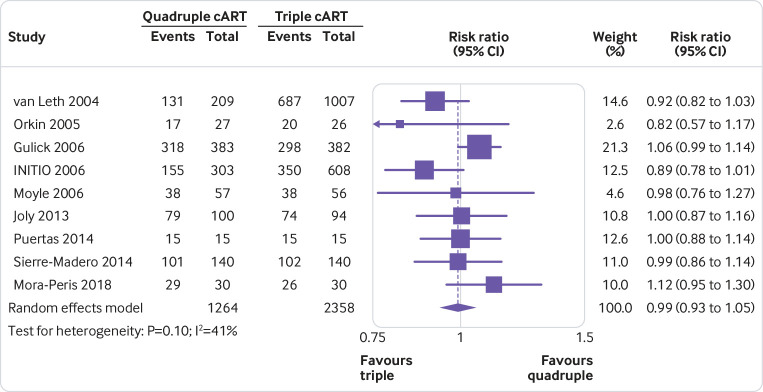
The figure displays for each study included in the meta-analysis the summary statistics (number of events and sample size) for the quadruple and triple combination antiretroviral therapies (cART) groups, and the risk ratio and its 95% confidence interval for the dichotomous outcome, undetectable HIV-1 RNA. Reproduced from Feng et al.^192^

**Fig 3:**
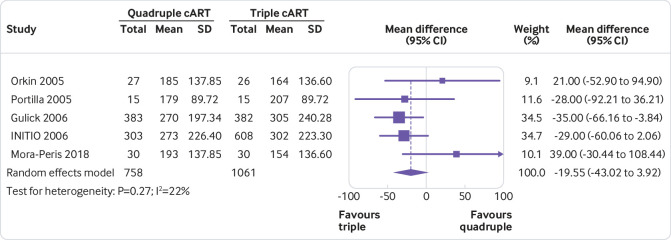
The figure displays for each study included in the meta-analysis the summary statistics (mean, standard deviation, and sample size) for the quadruple and triple combination antiretroviral therapies (cART) groups, and the mean difference and its 95% confidence interval for the continuous outcome, CD4 T cell count (cells/μL). Reproduced from Feng et al.^192^

For an example of individual study results presented for a dichotomous outcome, see figure 2. For an example of individual study results presented for a continuous outcome, see figure 3.^192^

## Results of syntheses

### Item 20a. For each synthesis, briefly summarise the characteristics and risk of bias among contributing studies

***Explanation:*** Many systematic review reports include narrative summaries of the characteristics and risk of bias across all included studies.^36^ However, such general summaries are not useful when the studies contributing to each synthesis vary, and particularly when there are many studies. For example, one meta-analysis might include three studies of participants aged 30 years on average, whereas another meta-analysis might include 10 studies of participants aged 60 years on average; in this case, knowing the mean age per synthesis is more meaningful than the overall mean age across all 13 studies. Providing a brief summary of the characteristics and risk of bias among studies contributing to each synthesis (meta-analysis or other) should help readers understand the applicability and risk of bias in the synthesised result. Furthermore, a summary at the level of the synthesis is more usable since it obviates the need for readers to refer to multiple sections of the review in order to interpret results.^92^

#### **Essential elements**

- Provide a brief summary of the characteristics and risk of bias among studies contributing to each synthesis (meta-analysis or other). The summary should focus only on study characteristics that help in interpreting the results (especially those that suggest the evidence addresses only a restricted part of the review question, or indirectly addresses the question). If the same set of studies contribute to more than one synthesis, or if the same risk of bias issues are relevant across studies for different syntheses, such a summary need be provided once only.
- Indicate which studies were included in each synthesis (such as by listing each study in a forest plot or table or citing studies in the text).

**Example of item 20a of PRISMA 2020 checklist**

“Nine randomized controlled trials (RCTs) directly compared delirium incidence between haloperidol and placebo groups [9 studies cited]. These RCTs enrolled 3,408 patients in both surgical and medical intensive care and non-intensive care unit settings and used a variety of validated delirium detection instruments. Five of the trials were low risk of bias [5 studies cited], three had unclear risk of bias [3 studies cited], and one had high risk of bias owing to lack of blinding and allocation concealment [1 study cited]. Intravenous haloperidol was administered in all except two trials; in those two exceptions, oral doses were given [two studies cited]. These nine trials were pooled, as they each identified new onset of delirium (incidence) within the week after exposure to prophylactic haloperidol or placebo.”^193^

### Item 20b. Present results of all statistical syntheses conducted. If meta-analysis was done, present for each the summary estimate and its precision (such as confidence/credible interval) and measures of statistical heterogeneity. If comparing groups, describe the direction of the effect

***Explanation:*** Users of reviews rely on the reporting of all statistical syntheses conducted so that they have complete and unbiased evidence on which to base their decisions. Studies examining selective reporting of results in systematic reviews have found that 11% to 22% of reviews did not present results for at least one pre-specified outcome of the review.^140^ ^141^ ^142^ ^143^

#### **Essential elements**

- Report results of all statistical syntheses described in the protocol and all syntheses conducted that were not pre-specified.
- If meta-analysis was conducted, report for each:
  - the summary estimate and its precision (such as standard error or 95% confidence/credible interval).
  - measures of statistical heterogeneity (such as τ^2^, I^2^, prediction interval).
- If other statistical synthesis methods were used (such as summarising effect estimates, combining P values), report the synthesised result and a measure of precision (or equivalent information, for example, the number of studies and total sample size).
- If the statistical synthesis method does not yield an estimate of effect (such as when P values are combined), report the relevant statistics (such as P value from the statistical test), along with an interpretation of the result that is consistent with the question addressed by the synthesis method (for example, “There was strong evidence of benefit of the intervention in at least one study (P < 0.001, 10 studies)” when P values have been combined).^28^
- If comparing groups, describe the direction of effect (such as fewer events in the intervention group, or higher pain in the comparator group).
- If synthesising mean differences, specify for each synthesis, where applicable, the unit of measurement (such as kilograms or pounds for weight), the upper and lower limits of the measurement scale (for example, anchors range from 0 to 10), direction of benefit (for example, higher scores denote higher severity of pain), and the minimally important difference, if known. If synthesising standardised mean differences and the effect estimate is being re-expressed to a particular instrument, details of the instrument, as per the mean difference, should be reported.

**Example of item 20b of PRISMA 2020 checklist**

“Twelve studies, including a total of 159,086 patients, reported on the rate of major bleeding complications. Aspirin use was associated with a 46% relative risk increase of major bleeding complications (risk ratio 1.46; 95% CI, 1.30-1.64; p < 0.00001; I^2^ = 31%; absolute risk increase 0.077%; number needed to treat to harm 1295)”^194^

### Item 20c. Present results of all investigations of possible causes of heterogeneity among study results

***Explanation:*** Presenting results from all investigations of possible causes of heterogeneity among study results is important for users of reviews and for future research. For users, understanding the factors that may, and equally, may not, explain variability in the effect estimates, may inform decision making. Similarly, presenting all results is important for designing future studies. For example, the results may help to generate hypotheses about potential modifying factors that can be tested in future studies, or help identify “active” intervention ingredients that might be combined and tested in a future randomised trial. Selective reporting of the results leads to an incomplete representation of the evidence that risks misdirecting decision making and future research.

#### **Essential elements**

- If investigations of possible causes of heterogeneity were conducted:
  - present results regardless of the statistical significance, magnitude, or direction of effect modification.
  - identify the studies contributing to each subgroup.
  - report results with due consideration to the observational nature of the analysis and risk of confounding due to other factors.^109^ ^144^
- If subgroup analysis was conducted, report for each analysis the exact P value for a test for interaction as well as, within each subgroup, the summary estimates, their precision (such as standard error or 95% confidence/credible interval) and measures of heterogeneity. Results from subgroup analyses might usefully be presented graphically (see Fisher et al^121^).
- If meta-regression was conducted, report for each analysis the exact P value for the regression coefficient and its precision.
- If informal methods (that is, those that do not involve a formal statistical test) were used to investigate heterogeneity—which may arise particularly when the data are not amenable to meta-analysis—describe the results observed. For example, present a table that groups study results by dose or overall risk of bias and comment on any patterns observed.^116^

#### **Additional elements**

- If subgroup analysis was conducted, consider presenting the estimate for the difference between subgroups and its precision.
- If meta-regression was conducted, consider presenting a meta-regression scatterplot with the study effect estimates plotted against the potential effect modifier.^109^

**Example of item 20c of PRISMA 2020 checklist**

“Among the 4 trials that recruited critically ill patients who were and were not receiving invasive mechanical ventilation at randomization, the association between corticosteroids and lower mortality was less marked in patients receiving invasive mechanical ventilation (ratio of odds ratios (ORs), 4.34 [95% CI, 1.46-12.91]; P = 0.008 based on within-trial estimates combined across trials); however, only 401 patients (120 deaths) contributed to this comparison…All trials contributed data according to age group and sex. For the association between corticosteroids and mortality, the OR was 0.69 (95% CI, 0.51-0.93) among 880 patients older than 60 years, the OR was 0.67 (95% CI, 0.48-0.94) among 821 patients aged 60 years or younger (ratio of ORs, 1.02 [95% CI, 0.63-1.65], P = 0.94), the OR was 0.66 (95% CI, 0.51-0.84) among 1215 men, and the OR was 0.66 (95% CI, 0.43-0.99) among 488 women (ratio of ORs, 1.07 [95% CI, 0.58-1.98], P = 0.84).”^195^

### Item 20d. Present results of all sensitivity analyses conducted to assess the robustness of the synthesised results

***Explanation:*** Presenting results of sensitivity analyses conducted allows readers to assess how robust the synthesised results were to decisions made during the review process. Reporting results of all sensitivity analyses is important; presentation of a subset, based on the nature of the results, risks introducing bias due to selective reporting. Forest plots are a useful way to present results of sensitivity analyses; however, these may be best placed in an appendix, with the main forest plots presented in the main report, to not reduce readability. An exception may be when sensitivity analyses reveal the results are not robust to decisions made during the review process.

#### **Essential elements**

- If any sensitivity analyses were conducted:
  - report the results for each sensitivity analysis.
  - comment on how robust the main analysis was given the results of all corresponding sensitivity analyses.

#### **Additional elements**

- If any sensitivity analyses were conducted, consider:
  - presenting results in tables that indicate: (i) the summary effect estimate, a measure of precision (and potentially other relevant statistics, for example, *I^2^* statistic) and contributing studies for the original meta-analysis; (ii) the same information for the sensitivity analysis; and (iii) details of the original and sensitivity analysis assumptions.
  - presenting results of sensitivity analyses visually using forest plots.

**Example of item 20d of PRISMA 2020 checklist**

“Sensitivity analyses that removed studies with potential bias showed consistent results with the primary meta-analyses (risk ratio 1.00 for undetectable HIV-1 RNA, 1.00 for virological failure, 0.98 for severe adverse effects, and 1.02 for AIDS defining events; supplement 3E, 3F, 3H, and 3I, respectively). Such sensitivity analyses were not performed for other outcomes because none of the studies reporting them was at a high risk of bias. Sensitivity analysis that pooled the outcome data reported at 48 weeks, which also showed consistent results, was performed for undetectable HIV-1 RNA and increase in CD4 T cell count only (supplement 3J and 3K) and not for other outcomes owing to lack of relevant data. When the standard deviations for increase in CD4 T cell count were replaced by those estimated by different methods, the results of figure 3 either remained similar (that is, quadruple and triple arms not statistically different) or favoured triple therapies (supplement 2).”^192^

## Risk of reporting biases in syntheses

### Item 21. Present assessments of risk of bias due to missing results (arising from reporting biases) for each synthesis assessed

***Explanation:*** Presenting assessments of the risk of bias due to missing results in syntheses allows readers to assess potential threats to the trustworthiness of a systematic review’s results. Providing the evidence used to support judgments of risk of bias allows readers to determine the validity of the assessments.

#### **Essential elements**

- Present assessments of risk of bias due to missing results (arising from reporting biases) for each synthesis assessed.
- If a tool was used to assess risk of bias due to missing results in a synthesis, present responses to questions in the tool, judgments about risk of bias, and any information used to support such judgments to help readers understand why particular judgments were made.
- If a funnel plot was generated to evaluate small-study effects (one cause of which is reporting biases), present the plot and specify the effect estimate and measure of precision used in the plot (presented typically on the horizontal axis and vertical axis respectively^106^). If a contour-enhanced funnel plot was generated, specify the “milestones” of statistical significance that the plotted contour lines represent (P=0.01, 0.05, 0.1, etc).^145^
- If a test for funnel plot asymmetry was used, report the exact P value observed for the test and potentially other relevant statistics, such as the standardised normal deviate, from which the P value is derived.^106^
- If any sensitivity analyses seeking to explore the potential impact of missing results on the synthesis were conducted, present results of each analysis (see item #20d), compare them with results of the primary analysis, and report results with due consideration of the limitations of the statistical method.^123^

#### **Additional elements**

- If studies were assessed for selective non-reporting of results by comparing outcomes and analyses pre-specified in study registers, protocols, and statistical analysis plans with results that were available in study reports, consider presenting a matrix (with rows as studies and columns as syntheses) to present the availability of study results.^124^
- If an assessment of selective non-reporting of results reveals that some studies are missing from the synthesis, consider displaying the studies with missing results underneath a forest plot or including a table with the available study results (for example, see forest plot in Page et al^81^).

**Example of item 21 of PRISMA 2020 checklist**

“Clinical global impression of change was assessed in Doody 2008, NCT00912288, CONCERT and CONNECTION using the CIBIC-Plus. However, we were only able to extract results from Doody 2008 [because no results for CIBIC-Plus were reported in the other three studies]…The authors reported small but significant improvements on the CIBIC‐Plus for 183 patients (89 on latrepirdine and 94 on placebo) favouring latrepirdine following the 26‐week primary endpoint (MD −0.60, 95% CI −0.89 to −0.31, P<0.001). Similar results were found at the additional 52‐week follow‐up (MD −0.70, 95% CI −1.01 to −0.39, P<0.001). However, we considered this to be low quality evidence due to imprecision and reporting bias. Thus, we could not draw conclusions about the efficacy of latrepirdine in terms of changes in clinical impression.”^196^

## Certainty of evidence

### Item 22. Present assessments of certainty (or confidence) in the body of evidence for each outcome assessed

***Explanation:*** An important feature of systems for assessing certainty, such as GRADE, is explicit reporting of both the level of certainty (or confidence) in the evidence and the basis for judgments.^97^ ^98^ ^127^ Evidence summary tables, such as GRADE Summary of Findings tables, are an effective and efficient way to report assessments of the certainty of evidence.^97^ ^127^ ^146^ ^147^

#### **Essential elements**

- Report the overall level of certainty in the body of evidence (such as high, moderate, low, or very low) for each important outcome.
- Provide an explanation of reasons for rating down (or rating up) the certainty of evidence (such as in footnotes to an evidence summary table). Explanations for each judgment should be concise, informative, relevant to the target audience, easy to understand, and accurate (that is, addressing criteria specified in the methods guidance).^148^
- Communicate certainty in the evidence wherever results are reported (that is, abstract, evidence summary tables, results, conclusions). Use a format appropriate for the section of the review. For example, in text, certainty might be reported explicitly in a sentence (such as “Moderate-certainty evidence (downgraded for bias) indicates that…”) or in brackets alongside an effect estimate (such as “[RR 1.17, 95% CI 0.81 to 1.68; 4 studies, 1781 participants; moderate certainty evidence]”). When interpreting results in “summary of findings” tables or conclusions, certainty might be communicated implicitly using standard phrases (such as “Hip protectors probably reduce the risk of hip fracture slightly”).^130^

#### **Additional elements**

- Consider including evidence summary tables, such as GRADE Summary of Findings tables.

**Example of item 22 of PRISMA 2020 checklist**

“Compared with non-operative treatment, low-certainty evidence indicates surgery (repair with subacromial decompression) may have little or no effect on function at 12 months. The evidence was downgraded two steps, once for bias and once for imprecision—the 95% CIs overlap minimal important difference in favour of surgery at this time point.” A summary of findings table presents the same information as the text above, with footnotes explaining judgments.^187^

## Discussion

### Item 23a. Provide a general interpretation of the results in the context of other evidence

***Explanation:*** Discussing how the results of the review relate to other relevant evidence should help readers interpret the findings. For example, authors might compare the current results to results of other similar systematic reviews (such as reviews that addressed the same question using different methods or that addressed slightly different questions) and explore possible reasons for discordant results. Similarly, authors might summarise additional information relevant to decision makers that was not explored in the review, such as findings of studies evaluating the cost-effectiveness of the intervention or surveys gauging the values and preferences of patients.

#### **Essential elements**

- Provide a general interpretation of the results in the context of other evidence.

**Example of item 23a of PRISMA 2020 checklist**

“Although we need to exercise caution in interpreting these findings because of the small number of studies, these findings nonetheless appear to be largely in line with the recent systematic review on what works to improve education outcomes in low‐ and middle‐income countries of Snilstveit et al. (2012). They found that structured pedagogical interventions may be among the effective approaches to improve learning outcomes in low‐ and middle‐income countries. This is consistent with our findings that teacher training is only effective in improving early grade literacy outcomes when it is combined with teacher coaching. The finding is also consistent with our result that technology in education programs may have at best no effects unless they are combined with a focus on pedagogical practices. In line with our study, Snilstveit et al. (2012) also do not find evidence for statistically significant effects of the one‐laptop‐per‐child program. These results are consistent with the results of a meta‐analysis showing that technology in education programs are not effective when not accompanied by parent or student training (McEwan, 2015). However, neither Snilstveit et al. (2012) nor McEwan (2015) find evidence for negative effects of the one‐laptop‐per‐child program on early grade literacy outcomes.”^197^

### Item 23b. Discuss any limitations of the evidence included in the review

***Explanation:*** Discussing the completeness, applicability, and uncertainties in the evidence included in the review should help readers interpret the findings appropriately. For example, authors might acknowledge that they identified few eligible studies or studies with a small number of participants, leading to imprecise estimates; have concerns about risk of bias in studies or missing results; or identified studies that only partially or indirectly address the review question, leading to concerns about their relevance and applicability to particular patients, settings, or other target audiences. The assessments of certainty (or confidence) in the body of evidence (item #22) can support the discussion of such limitations.

#### **Essential elements**

- Discuss any limitations of the evidence included in the review.

**Example of item 23b of PRISMA 2020 checklist**

“Study populations were young, and few studies measured longitudinal exposure. The included studies were often limited by selection bias, recall bias, small sample of marijuana-only smokers, reporting of outcomes on marijuana users and tobacco users combined, and inadequate follow-up for the development of cancer…Most studies poorly assessed exposure, and some studies did not report details on exposure, preventing meta-analysis for several outcomes.”^198^

### Item 23c. Discuss any limitations of the review processes used

***Explanation:*** Discussing limitations, avoidable or unavoidable, in the review process should help readers understand the trustworthiness of the review findings. For example, authors might acknowledge the decision to restrict eligibility to studies in English only, search only a small number of databases, have only one reviewer screen records or collect data, or not contact study authors to clarify unclear information. They might also acknowledge that they were unable to access all potentially eligible study reports or to carry out some of the planned analyses because of insufficient data.^149^ ^150^ While some limitations may affect the validity of the review findings, others may not.

#### **Essential elements**

- Discuss any limitations of the review processes used and comment on the potential impact of each limitation.

**Example of item 23c of PRISMA 2020 checklist**

“Because of time constraints…we dually screened only 30% of the titles and abstracts; for the rest, we used single screening. A recent study showed that single abstract screening misses up to 13% of relevant studies (Gartlehner 2020). In addition, single review authors rated risk of bias, conducted data extraction and rated certainty of evidence. A second review author checked the plausibility of decisions and the correctness of data. Because these steps were not conducted dually and independently, we introduced some risk of error…Nevertheless, we are confident that none of these methodological limitations would change the overall conclusions of this review. Furthermore, we limited publications to English and Chinese languages. Because COVID-19 has become a rapidly evolving pandemic, we might have missed recent publications in languages of countries that have become heavily affected in the meantime (e.g. Italian or Spanish).” 199

### Item 23d. Discuss implications of the results for practice, policy, and future research

***Explanation:*** There are many potential end users of a systematic review (such as patients, healthcare providers, researchers, insurers, and policy makers), each of whom will want to know what actions they should take given the review findings. Patients and healthcare providers may be primarily interested in the balance of benefits and harms, while policy makers and administrators may value data on organisational impact and resource utilisation. For reviews of interventions, authors might clarify trade-offs between benefits and harms and how the values attached to the most important outcomes of the review might lead different people to make different decisions. In addition, rather than making recommendations for practice or policy that apply universally, authors might discuss factors that are important in translating the evidence to different settings and factors that may modify the magnitude of effects.

Explicit recommendations for future research—as opposed to general statements such as “More research on this question is needed”—can better direct the questions future studies should address and the methods that should be used. For example, authors might consider describing the type of understudied participants who should be enrolled in future studies, the specific interventions that could be compared, suggested outcome measures to use, and ideal study design features to employ.

#### **Essential elements**

- Discuss implications of the results for practice and policy.
- Make explicit recommendations for future research.

**Example of item 23d of PRISMA 2020 checklist**

“Implications for practice and policy: Findings from this review indicate that bystander programs have significant beneficial effects on bystander intervention behaviour. This provides important evidence of the effectiveness of mandated programs on college campuses. Additionally, the fact that our (preliminary) moderator analyses found program effects on bystander intervention to be similar for adolescents and college students suggests early implementation of bystander programs (i.e. in secondary schools with adolescents) may be warranted. Importantly, although we found that bystander programs had a significant beneficial effect on bystander intervention behaviour, we found no evidence that these programs had an effect on participants' sexual assault perpetration. Bystander programs may therefore be appropriate for targeting bystander behaviour, but may not be appropriate for targeting the behaviour of potential perpetrators. Additionally, effects of bystander programs on bystander intervention behaviour diminished by 6‐month post‐intervention. Thus, programs effects may be prolonged by the implementation of booster sessions conducted prior to 6 months post‐intervention.

Implications for research: Findings from this review suggest there is a fairly strong body of research assessing the effects of bystander programs on attitudes and behaviours. However, there are a couple of important questions worth further exploration…Our understanding of the causal mechanisms of program effects on bystander behaviour would benefit from further analysis (e.g., path analysis mapping relationships between specific knowledge/attitude effects and bystander intervention)…Our understanding of the differential effects of gendered versus gender neutral programs would benefit from the design and implementation of high-quality primary studies that make direct comparisons between these two types of programs (e.g., RCTs comparing the effects of two active treatment arms that differ in their gendered approach)…Our understanding of bystander programs' generalizability to non-US contexts would be greatly enhanced by high quality research conducted across the world.”^200^

## Registration and protocol

### Item 24a. Provide registration information for the review, including register name and registration number, or state that the review was not registered

***Explanation:*** Stating where the systematic review was registered (such as PROSPERO, Open Science Framework) and the registration number or DOI for the register entry (see box 6) facilitates identification of the systematic review in the register. This allows readers to compare what was pre-specified with what was eventually reported in the review and decide if any deviations may have introduced bias. Reporting registration information also facilitates linking of publications related to the same systematic review (such as when a review is presented at a conference and published in a journal).^154^

Box 6 **Systematic review registration and protocols**

Registration aims to reduce bias, increase transparency, facilitate scrutiny and improve trustworthiness of systematic reviews.^151^ ^152^ Registration also aims to reduce unintended duplication; researchers planning a new review should search register listings to identify similar completed or ongoing reviews before deciding whether their review is needed, noting that planned duplication may be justified.^151^

A registration entry captures key elements of the review protocol and is submitted to a host register, ideally before starting the review. The register maintains a permanent public record of this information along with any subsequent amendments (date-stamped) and issues a unique number to link the registration entry to completed review publications.^153^ Publicly recording details of inclusion and exclusion criteria, planned outcomes, and syntheses enables peer reviewers, journal editors, and readers to compare the completed review with what was planned, identify any deviations, and decide whether these may have introduced bias.

PROSPERO (www.crd.york.ac.uk/prospero/) currently registers systematic reviews with direct health outcomes. It also accepts systematic reviews of animal studies that have direct implications for human health, and methodology reviews which have direct bearing on human health or systematic review conduct. Reviews not meeting the criteria for inclusion in PROSPERO could be registered elsewhere; for example, in the Open Science Framework (OSF) repository. Both PROSPERO and OSF allow for registration without cost.

A review protocol is distinct from a register entry for a review. A review protocol outlines in detail the pre-planned objectives and methods intended to be used to conduct the review, helping to anticipate/avoid potential problems before embarking on a review and providing a methodical approach to prevent arbitrary decision making during the review process.^22^ Systematic reviewers are encouraged to report their protocols in accordance with the PRISMA guidance for protocols (PRISMA-P).^21^ PRISMA-P consists of a checklist^21^ accompanied by a detailed guidance document providing researchers with a step-by-step approach for documenting a systematic review protocol.^22^

A review protocol should be a public document in order to facilitate future purposeful replications or updates of the review and to help future users evaluate whether selective reporting and potential bias were present in the review process.^22^ Review protocols can be made public through one of several routes. One option is to upload a PDF of the protocol to the corresponding PROSPERO registration record so they are linked in perpetuity. Another option is to make a protocol a document with its own unique identifier (that is, a DOI) so it can be cited across various documents including the PROSPERO registration record and in the full text of the completed review. To achieve this, reviewers may opt to publish a protocol in a journal that is open access or provides free access to content (such as *Systematic Reviews*, *BMJ Open*) or a journal using the Registered Reports publishing framework (https://cos.io/rr/), where it will benefit from external feedback before publication, or deposit a protocol in a general purpose or institutional open access repository (such as Open Science Framework Registries, Zenodo).

#### **Essential elements**

- Provide registration information for the review, including register name and registration number, or state that the review was not registered.

**Example of item 24a of PRISMA 2020 checklist**

“…this systematic review has been registered in the international prospective register of systematic reviews (PROSPERO) under the registration number: CRD42019128569”^201^

### Item 24b. Indicate where the review protocol can be accessed, or state that a protocol was not prepared

***Explanation:*** The review protocol may contain information about the methods that is not provided in the final review report (see box 6). Providing a citation, DOI, or link to the review protocol allows readers to locate the protocol more easily. Comparison of the methods pre-specified in the review protocol with what was eventually done allows readers to assess whether any deviations may have introduced bias.^155^ If the review protocol was not published or deposited in a public repository, or uploaded as a supplementary file to the review report, we recommend providing the contact details of the author responsible for sharing the protocol. If authors did not prepare a review protocol, or prepared one but are not willing to make it accessible, this should be stated to prevent users spending time trying to locate the document.

#### **Essential elements**

- Indicate where the review protocol can be accessed (such as by providing a citation, DOI, or link) or state that a protocol was not prepared.

**Example of item 24b of PRISMA 2020 checklist**

“…this systematic review and meta-analysis protocol has been published elsewhere [citation for the protocol provided].”^202^

### Item 24c. Describe and explain any amendments to information provided at registration or in the protocol

***Explanation:*** Careful consideration of a review’s methodological and analytical approach early on is likely to lessen unnecessary changes after protocol development.^22^ However, it is difficult to anticipate all scenarios that will arise, necessitating some clarifications, modifications, and changes to the protocol (such as data available may not be amenable to the planned meta-analysis).^155^ ^156^ For reasons of transparency, authors should report details of any amendments. Amendments could be recorded in various places, including the full text of the review, a supplementary file, or as amendments to the published protocol or registration record.

#### **Essential elements**

- Report details of any amendments to information provided at registration or in the protocol, noting: (*a*) the amendment itself, (*b*) the reason for the amendment, and (*c*) the stage of the review process at which the amendment was implemented.

**Example of item 24c of PRISMA 2020 checklist**

“Differences from protocol: We modified the lower limit for age in our eligibility criteria from 12 years of age to 10 years of age because the age of adolescence was reduced. We used the WHO measures for severe anaemia, defined by haemoglobin levels < 80 g/L instead of < 70 g/L as stated in the protocol. We decided to add adverse events to our list of primary outcomes (instead of secondary) and we changed reinfection rate to a secondary outcome.”^203^

## Support

### Item 25. Describe sources of financial or non-financial support for the review, and the role of the funders or sponsors in the review

***Explanation:*** As with any research report, authors should be transparent about the sources of support received to conduct the review. For example, funders may provide salary to researchers to undertake the review, the services of an information specialist to conduct searches, or access to commercial databases that would otherwise not have been available. Authors may have also obtained support from a translation service to translate articles or in-kind use of software to manage or analyse the study data. In some reviews, the funder or sponsor (that is, the individual or organisation assuming responsibility for the initiation and management of the review) may have contributed to defining the review question, determining eligibility of studies, collecting data, analysing data, interpreting results, or approving the final review report. There is potential for bias in the review findings arising from such involvement, particularly when the funder or sponsor has an interest in obtaining a particular result.^157^

#### **Essential elements**

- Describe sources of financial or non-financial support for the review, specifying relevant grant ID numbers for each funder. If no specific financial or non-financial support was received, this should be stated.
- Describe the role of the funders or sponsors (or both) in the review. If funders or sponsors had no role in the review, this should be declared—for example, by stating, “The funders had no role in the design of the review, data collection and analysis, decision to publish, or preparation of the manuscript.”

**Example of item 25 of PRISMA 2020 checklist**

“Funding/Support: This research was funded under contract HHSA290201500009i, Task Order 7, from the Agency for Healthcare Research and Quality (AHRQ), US Department of Health and Human Services, under a contract to support the US Preventive Services Task Force (USPSTF). Role of the Funder/Sponsor: Investigators worked with USPSTF members and AHRQ staff to develop the scope, analytic framework, and key questions for this review. AHRQ had no role in study selection, quality assessment, or synthesis. AHRQ staff provided project oversight, reviewed the report to ensure that the analysis met methodological standards, and distributed the draft for peer review. Otherwise, AHRQ had no role in the conduct of the study; collection, management, analysis, and interpretation of the data; and preparation, review, or approval of the manuscript findings. The opinions expressed in this document are those of the authors and do not reflect the official position of AHRQ or the US Department of Health and Human Services.”^204^

## Competing interests

### Item 26. Declare any competing interests of review authors

***Explanation:*** Authors of a systematic review may have relationships with organisations or entities with an interest in the review findings (for example, an author may serve as a consultant for a company manufacturing the drug or device under review).^158^ Such relationships or activities are examples of a competing interest (or conflict of interest), which can negatively affect the integrity and credibility of systematic reviews. For example, evidence suggests that systematic reviews with financial competing interests more often have conclusions favourable to the experimental intervention than systematic reviews without financial competing interests.^159^ Information about authors’ relationships or activities that readers could consider pertinent or to have influenced the review should be disclosed using the format requested by the publishing entity (such as using the International Committee of Medical Journal Editors (ICMJE) disclosure form).^160^ Authors should report how competing interests were managed for particular review processes. For example, if a review author was an author of an included study, they may have been prevented from assessing the risk of bias in the study results.

#### **Essential elements**

- Disclose any of the authors’ relationships or activities that readers could consider pertinent or to have influenced the review.
- If any authors had competing interests, report how they were managed for particular review processes.

**Example of item 26 of PRISMA 2020 checklist**

“Declarations of interest: R Buchbinder was a principal investigator of Buchbinder 2009. D Kallmes was a principal investigator of Kallmes 2009 and Evans 2015. D Kallmes participated in IDE trial for Benvenue Medical spinal augmentation device. He is a stockholder, Marblehead Medical, LLC, Development of spine augmentation devices. He holds a spinal fusion patent license, unrelated to spinal augmentation/vertebroplasty. R Buchbinder and D Kallmes did not perform risk of bias assessments for their own or any other placebo‐controlled trials included in the review.”^205^

## Availability of data, code, and other materials

### Item 27. Report which of the following are publicly available and where they can be found: template data collection forms; data extracted from included studies; data used for all analyses; analytic code; any other materials used in the review

***Explanation:*** Sharing of data, analytic code, and other materials enables others to reuse the data, check the data for errors, attempt to reproduce the findings, and understand more about the analysis than may be provided by descriptions of methods.^161^ ^162^ Support for sharing of data, analytic code, and other materials is growing, including from patients^163^ and journal editors, including *BMJ* and *PLOS Medicine*.^164^

Sharing of data, analytic code, and other materials relevant to a systematic review includes making various items publicly available, such as the template data collection forms; all data extracted from included studies; a file indicating necessary data conversions; the clean dataset(s) used for all analyses in a format ready for reuse (such as CSV file); metadata (such as complete descriptions of variable names, README files describing each file shared); analytic code used in software with a command-line interface or complete descriptions of the steps used in point-and-click software to run all analyses. Other materials might include more detailed information about the intervention delivered in the primary studies that are otherwise not available, such as a video of the specific cognitive behavioural therapy supplied by the study investigators to reviewers.^73^ Similarly, other material might include a list of all citations screened and any decisions about eligibility.

Because sharing of data, analytic code, and other materials is not yet universal in health and medical research,^164^ even interested authors may not know how to make their materials publicly available. Data, analytic code, and other materials can be uploaded to one of several publicly accessible repositories (such as Open Science Framework, Dryad, figshare). The Systematic Review Data Repository (https://srdr.ahrq.gov/) is another example of a platform for sharing materials specific to the systematic review community.^165^ All of these open repositories should be given consideration, particularly if the completed review is to be considered for publication in a paywalled journal. The Findable, Accessible, Interoperable, Reusable (FAIR) data principles are also a useful resource for authors to consult,^166^ as they provide guidance on the best way to share information.

There are some situations where authors might not be able to share review materials, such as when the review team are custodians rather than owners of individual participant data, or when there are legal or licensing restrictions. For example, records exported directly from bibliographic databases (such as Ovid MEDLINE) typically include copyrighted material; authors should read the licensing terms of the databases they search to see what they can share and to consider the copyright legislation of their countries.

#### **Essential elements**

- Report which of the following are publicly available: template data collection forms; data extracted from included studies; data used for all analyses; analytic code; any other materials used in the review.
- If any of the above materials are publicly available, report where they can be found (such as provide a link to files deposited in a public repository).
- If data, analytic code, or other materials will be made available upon request, provide the contact details of the author responsible for sharing the materials and describe the circumstances under which such materials will be shared.

**Example of item 27 of PRISMA 2020 checklist**

“All meta-analytic data and all codebooks and analysis scripts (for Mplus and R) are publicly available at the study’s associated page on the Open Science Framework (https://osf.io/r8a24/)...The precise sources (table, section, or paragraph) for each estimate are described in notes in the master data spreadsheet, available on the Open Science Framework page for this study (https://osf.io/r8a24/)”^206^

## Conclusion to PRISMA 2020 explanation and elaboration

This explanation and elaboration paper has been designed to assist authors seeking comprehensive guidance on what to include in systematic review reports. We hope that use of this resource will lead to more transparent, complete, and accurate reporting of systematic reviews, thus facilitating evidence-based decision making.

## Web Extra

Extra material supplied by the author

Further examples of good reporting practice

## Notes

Contributors: DM and JEM are joint senior authors. MJP, JEM, PMB, IB, TCH, CDM, LS and DM conceived this paper and designed the literature review and survey conducted to inform the guideline content. MJP conducted the literature review, administered the survey and analysed the data for both. MJP prepared all materials for the development meeting. MJP and JEM presented proposals at the development meeting. All authors except for TCH, JMT, EAA, SEB and LAM attended the development meeting. MJP and JEM took and consolidated notes from the development meeting. MJP and JEM led the drafting and editing of the article. JEM, PMB, IB, TCH, LS, JMT, EAA, SEB, RC, JG, AH, TL, EMW, SM, LAM, LAS, JT, ACT, PW and DM drafted particular sections of the article. All authors were involved in revising the article critically for important intellectual content. All authors approved the final version of the article. MJP is the guarantor of this work. The corresponding author attests that all listed authors meet authorship criteria and that no others meeting the criteria have been omitted.

Patient and public involvement: Patients and the public were not involved in this methodological research. We plan to disseminate the research widely, including to community participants in evidence synthesis organisations.

Provenance and peer review: Not commissioned; externally peer reviewed.
