# Supplementary material for: PRISMA 2020 explanation and elaboration: updated guidance and exemplars for reporting systematic reviews
Source: BMJ. 2021 Mar 29;372:n160. doi: 10.1136/bmj.n160 (PMC8005925; doi:10.1136/bmj.n160)
Supplement: Supplementary file 1 — Further examples of good reporting practice [file pagm061901.w1.pdf]

**Supplementary Material to:**

# PRISMA 2020 explanation and elaboration: updated guidance and exemplars for reporting systematic reviews

Matthew J Page*, David Moher, Patrick M Bossuyt, Isabelle Boutron, Tammy C Hoffmann, Cynthia D Mulrow, Larissa Shamseer, Jennifer M Tetzlaff, Elie A Akl, Sue E Brennan, Roger Chou, Julie Glanville, Jeremy M Grimshaw, Asbjørn Hróbjartsson, Manoj M Lalu, Tianjing Li, Elizabeth W Loder, Evan Mayo-Wilson, Steve McDonald, Luke A McGuinness, Lesley A Stewart, James Thomas, Andrea C Tricco, Vivian A Welch, Penny Whiting, Joanne E McKenzie

***Correspondence to**: Dr. Matthew Page, School of Public Health and Preventive Medicine, Monash University, 553 St Kilda Road, Melbourne, Victoria, 3004, Australia. Email address: matthew.page@monash.edu

**Supplementary Table 1. PRISMA 2020 examples**

Note: We edited the examples by removing all citations within them (to avoid potential confusion with the citation for each example) and spelled out abbreviations to aid comprehension.

| Checklist item | Examples |
|---|---|
| Item 1. TITLE: Identify the report as a systematic review. | **Example 1: In a review examining the effects of rivaroxaban versus warfarin for people with antiphospholipid syndrome, the authors identify the report as a systematic review:**<br>“Comparison of the Therapeutic Effects of Rivaroxaban Versus Warfarin in Antiphospholipid Syndrome: A Systematic Review” (1)<br>**Example 2: In a review examining the effects of repetitive transcranial magnetic stimulation on patients post-stroke, the authors identify the report as a systematic review and note that meta-analysis was conducted:**<br>“Repetitive transcranial magnetic stimulation for the treatment of lower limb dysfunction in patients poststroke: a systematic review with meta-analysis” (2)<br>**Example 3: In a review examining the effects of cannabis-based medicines for cancer pain, the authors identify the report as a systematic review with meta-analysis, and indicate that the review includes evidence from randomized trials only:**<br>“Efficacy, tolerability and safety of cannabis-based medicines for cancer pain: A systematic review with meta-analysis of randomised controlled trials” (3)<br>**Example 4: In a review examining the effects of routine anti-osteoporosis medication in men, the authors identify the report as an *updated* systematic review with meta-analysis:**<br>“Does routine anti-osteoporosis medication lower the risk of fractures in male subjects? An updated systematic review with meta-analysis of clinical trials” (4) |
| Item 2. ABSTRACT: See the PRISMA 2020 for Abstracts checklist. | **Example 1: In a review examining the effects of psychological interventions for common mental disorders in women experiencing intimate partner violence in low-income and middle-income countries, the authors summarise the objectives, eligibility criteria, databases consulted, methods for collecting data, assessing risk of bias and synthesising results, along with presenting results and commenting on the limitations of the evidence, and specify the registration number and funding source for the review.**<br>“Background: Evidence on the effectiveness of psychological interventions for women with common mental disorders (CMDs) who also experience intimate partner violence is scarce. We aimed to test our hypothesis that exposure to intimate partner violence would reduce intervention effectiveness for CMDs in low-income and middle-income countries (LMICs). Methods: For |

| Checklist item | Examples |
|---|---|
| | this systematic review and meta-analysis, we searched MEDLINE, Embase, PsycINFO, Web of Knowledge, Scopus, CINAHL, LILACS, ScieELO, Cochrane, PubMed databases, trials registries, 3ie, Google Scholar, and forward and backward citations for studies published between database inception and Aug 16, 2019. All randomised controlled trials (RCTs) of psychological interventions for CMDs in LMICs which measured intimate partner violence were included, without language or date restrictions. We approached study authors to obtain unpublished aggregate subgroup data for women who did and did not report intimate partner violence. We did separate random-effects meta-analyses for anxiety, depression, post-traumatic stress disorder (PTSD), and psychological distress outcomes. Evidence from randomised controlled trials was synthesised as differences between standardised mean differences (SMDs) for change in symptoms, comparing women who did and who did not report intimate partner violence via random-effects meta-analyses. The quality of the evidence was assessed with the Cochrane risk of bias tool. This study is registered on PROSPERO, number CRD42017078611. Findings: Of 8122 records identified, 21 were eligible and data were available for 15 RCTs, all of which had a low to moderate risk of overall bias. Anxiety (five interventions, 728 participants) showed a greater response to intervention among women reporting intimate partner violence than among those who did not (difference in standardised mean differences [dSMD] 0.31, 95% CI 0.04 to 0.57, $I^2$=49.4%). No differences in response to intervention were seen in women reporting intimate partner violence for PTSD (eight interventions, n=1436; dSMD 0.14, 95% CI -0.06 to 0.33, $I^2$=42.6%), depression (12 interventions, n=2940; 0.10, -0.04 to 0.25, $I^2$=49.3%), and psychological distress (four interventions, n=1591; 0.07, -0.05 to 0.18, $I^2$=0.0%, p=0.681). Interpretation: Psychological interventions treat anxiety effectively in women with current or recent intimate partner violence exposure in LMICs when delivered by appropriately trained and supervised health-care staff, even when not tailored for this population or targeting intimate partner violence directly. Future research should investigate whether adapting evidence-based psychological interventions for CMDs to address intimate partner violence enhances their acceptability, feasibility, and effectiveness in LMICs. Funding: UK National Institute for Health Research ASSET and King's IoPPN Clinician Investigator Scholarship." (5)<br><br>**Example 2: In a review examining the effects of cognitive bias modification interventions for people with anxiety and depressive disorders, the authors summarise the objectives, eligibility criteria, databases consulted, methods for collecting data, assessing risk of bias and synthesising results, along with presenting results and commenting on the limitations of the evidence, and specify the registration number and funding source for the review.**<br><br>"Background: Cognitive bias modification (CBM) therapies, including attention bias modification, interpretation bias modification, or approach and avoidance training, are prototypical examples of mechanistically derived treatments, but their effectiveness is contentious. We aimed to assess the relative effectiveness of various CBM interventions for anxious and depressive symptomatology. Methods: For this systematic review and network meta-analysis, we searched PubMed, PsycINFO, Embase, and Cochrane Central Register from database inception up until Feb 7, 2020. We included randomised controlled trials of CBM versus control conditions or other forms of CBM for adults aged 18 years and older with clinical or subclinical anxiety or depression |

| Checklist item | Examples |
|---|---|
| | measured with a diagnostic interview or a validated clinical scale. We excluded studies comparing CBM with a non-CBM active intervention. Two researchers independently selected studies and evaluated risk of bias with the Cochrane Collaboration tool. Primary outcomes encompassed anxiety and depressive symptoms measured with validated clinical scales. We computed standardised mean differences (SMDs) with a restricted maximum likelihood random effects model. This study is registered with PROSPERO, CRD42018086113. Findings: From 2125 records we selected 85 trials, 65 (n=3897) on anxiety and 20 (n=1116) on depression. In a well-connected network of anxiety trials, interpretation bias modification outperformed waitlist (SMD -0.55, 95% CI -0.91 to -0.19) and sham training (SMD -0.30, -0.50 to -0.10) for the primary outcome. Attention bias modification showed benefits only in post-hoc sensitivity analyses excluding post-traumatic stress disorder trials. Prediction intervals for all findings were large, including an SMD of 0. Networks of depression trials displayed evidence of inconsistency. Only four randomised controlled trials had low risk of bias on all six domains assessed. Interpretation: CBM interventions showed consistent but small benefits; however heterogeneity and risk of bias undermine the reliability of these findings. Larger, definitive trials for interpretation bias modification for anxiety might be warranted, but insufficient evidence precludes conclusions for depression. Funding: Romanian Ministry of Research and Innovation, The National Council for Scientific Research-The Executive Agency for Higher Education, Research, Development and Innovation Funding." (6) <br><br> **Example 3: In a review examining the effects of interventions designed to reduce antibiotic use and/or inappropriate use in long-term care facilities, the authors summarise the objectives, eligibility criteria, databases searched, and methods to assess risk of bias and synthesise results, along with results of meta-analyses, indicating the number of studies and participants included in each:** <br><br> "Objectives: There are high levels of inappropriate antibiotic use in long-term care facilities (LTCFs). Our objective was to examine evidence of the effectiveness of interventions designed to reduce antibiotic use and/or inappropriate use in LTCFs. Design: Systematic review and meta-analysis. Data sources: MEDLINE, Embase and CINAHL from 1997 until November 2018. Eligibility criteria: Controlled and uncontrolled studies in LTCFs measuring intervention effects on rates of overall antibiotic use and/or appropriateness of use were included. Secondary outcomes were intervention implementation barriers from process evaluations. Data extraction and synthesis: Two reviewers independently applied the Cochrane Effective Practice and Organisation of Care group's resources to classify interventions and assess risk of bias. Meta-analyses used random effects models to pool results. Results: Of include studies (n=19), 10 had a control group and 17 had a high risk of bias. All interventions had multiple components. Eight studies (with high risk of bias) showed positive impacts on outcomes and included one of the following interventions: audit and feedback, introduction of care pathways or an infectious disease team. Meta-analyses on change in the percentage of residents on antibiotics (pooled relative risk (RR) (three studies, 6862 residents): 0.85, 95% CI: 0.61 to 1.18), appropriateness of decision to treat with antibiotics (pooled RR (three studies, 993 antibiotic orders): 1.10, 95% CI: 0.64 to 1.91) and appropriateness of antibiotic selection for respiratory tract infections (pooled RR (three studies, 292 orders): 1.15, 95% CI: |

| Checklist item | Examples |
|---|---|
| | 0.95 to 1.40), showed no significant intervention effects. However, meta-analyses only included results from intervention groups since most studies lacked a control group. Insufficient data prevented meta-analysis on other outcomes. Process evaluations (n=7) noted poor intervention adoption, low physician engagement and high staff turnover as barriers. Conclusions: There is insufficient evidence that interventions employed to date are effective at improving antibiotic use in LTCFs. Future studies should use rigorous study designs and tailor intervention implementation to the setting." (7) |
| | **Example 4: In a review examining the effect of dose and duration of reduction in dietary sodium on blood pressure levels, the authors summarise the objective, eligibility criteria, databases searched, methods for selecting studies, collecting data and synthesising results, along with results (clearly indicating of the direction of benefit) and registration number for the review:** |
| | "Objective: To examine the dose-response relation between reduction in dietary sodium and blood pressure change and to explore the impact of intervention duration. Design: Systematic review and meta-analysis following PRISMA guidelines. Data sources: Ovid MEDLINE(R), EMBASE, and Cochrane Central Register of Controlled Trials (Wiley) and reference lists of relevant articles up to 21 January 2019. Inclusion criteria: Randomised trials comparing different levels of sodium intake undertaken among adult populations with estimates of intake made using 24 hour urinary sodium excretion. Data extraction and analysis: Two of three reviewers screened the records independently for eligibility. One reviewer extracted all data and the other two reviewed the data for accuracy. Reviewers performed random effects meta-analyses, subgroup analyses, and meta-regression. Results: 133 studies with 12,197 participants were included. The mean reductions (reduced sodium v usual sodium) of 24 hour urinary sodium, systolic blood pressure (SBP), and diastolic blood pressure (DBP) were 130 mmol (95% confidence interval 115 to 145, P<0.001), 4.26 mm Hg (3.62 to 4.89, P<0.001), and 2.07 mm Hg (1.67 to 2.48, P<0.001), respectively. Each 50 mmol reduction in 24 hour sodium excretion was associated with a 1.10 mm Hg (0.66 to 1.54; P<0.001) reduction in SBP and a 0.33 mm Hg (0.04 to 0.63; P=0.03) reduction in DBP. Reductions in blood pressure were observed in diverse population subsets examined, including hypertensive and non-hypertensive individuals. For the same reduction in 24 hour urinary sodium there was greater SBP reduction in older people, non-white populations, and those with higher baseline SBP levels. In trials of less than 15 days' duration, each 50 mmol reduction in 24 hour urinary sodium excretion was associated with a 1.05 mm Hg (0.40 to 1.70; P=0.002) SBP fall, less than half the effect observed in studies of longer duration (2.13 mm Hg; 0.85 to 3.40; P=0.002). Otherwise, there was no association between trial duration and SBP reduction. Conclusions: The magnitude of blood pressure lowering achieved with sodium reduction showed a dose-response relation and was greater for older populations, non-white populations, and those with higher blood pressure. Short term studies underestimate the effect of sodium reduction on blood pressure. Systematic review registration: PROSPERO CRD42019140812." (8) |

| Checklist item | Examples |
|---|---|
| Item 3. RATIONALE: Describe the rationale for the review in the context of existing knowledge. | **Example 1: In a review examining the effects of physical distancing, face masks and eye protection to prevent person-to-person transmission of SARS-CoV-2, the authors report acknowledge the conflicting advice issued by different jurisdictions and note no previous review addressing their question exists:**<br><br>"To contain widespread infection and to reduce morbidity and mortality among health-care workers and others in contact with potentially infected people, jurisdictions have issued conflicting advice about physical or social distancing. Use of face masks with or without eye protection to achieve additional protection is debated in the mainstream media and by public health authorities, in particular the use of face masks for the general population; moreover, optimum use of face masks in health-care settings, which have been used for decades for infection prevention, is facing challenges amid personal protective equipment (PPE) shortages. Any recommendations about social or physical distancing, and the use of face masks, should be based on the best available evidence. Evidence has been reviewed for other respiratory viral infections, mainly seasonal influenza, but no comprehensive review is available of information on SARS-CoV-2 or related betacoronaviruses that have caused epidemics, such as severe acute respiratory syndrome (SARS) or Middle East respiratory syndrome (MERS). We, therefore, systematically reviewed the effect of physical distance, face masks, and eye protection on transmission of SARS-CoV-2, SARS-CoV, and MERS-CoV." (9)<br><br>**Example 2: In a review examining the effects of mass deworming for improving health and cognition of children in endemic helminth areas, the authors report using alternative synthesis methods to those used in a previous version of the review, to address new questions and overcome limitations of the previous review:**<br><br>"A recent Campbell systematic review and network meta-analysis (NMA) by members of our team (V.W., P.T., G.A.W., E.G., Z.B.), with 47 randomised trials and >1 million children, found little to no overall effect on growth, attention and school attendance (Welch et al., 2016). With NMA, we were able to explore the size of effect with different types and frequency of drugs and their combination with food or micronutrients; none of which contributed to larger effects. Our review also did not find larger effects in subgroups of children at the aggregate level across characteristics such as age, baseline nutritional status, prevalence or intensity of infection that have been postulated to be important (Welch et al., 2016). These analyses were conducted at the study level, rather than using data for each individual child, which limits the power to detect effect modification by individual participant characteristics. This review was therefore unable to identify whether mass deworming was more effective for children with certain characteristics. There was substantial unexplained heterogeneity between studies, with some studies finding larger effects than others, and no single individual-level, setting-level or methodology characteristic explaining this variation. Thus, we concluded that our analysis of effect modifiers was limited by the aggregate level data…We decided in collaboration with several authors of primary trials that there would be value in conducting an individual participant data (IPD) meta-analysis to explore the question of whether mass deworming is more effective for subgroups of children defined by characteristics such as infection |

| Checklist item | Examples |
|---|---|
| | intensity or status, age or nutritional status. This understanding could help to develop targeted strategies to reach these children better with deworming and guide policy regarding deworming.” (10)<br>**Example 3: In a review examining the effects of surgical treatments for women with stress urinary incontinence, the authors report the knowledge gap and limitations of the existing evidence base:**<br>“Currently there is no clear evidence to indicate which surgery is the best choice. It is unclear if the older operations that were previously available (such as anterior repair and colposuspension) really result in equivalent or better outcomes than the polypropylene mid-urethral sling. However, the feeling of our clinical experts who used to offer colposuspension and traditional slings is that these techniques had more frequent and severe associated complications and returning to them may be detrimental to women. To enable women to make an evidence-based choice and inform practice guidelines, it is essential to collect reliable evidence in a transparent, concise manner to allow impartial counselling of women regarding the benefits and risks of the alternative surgical operations for the management of stress urinary incontinence. The wide range of surgical operations available, the different techniques used to perform these operations and the lack of a consensus among surgeons make it challenging to establish which procedure is the most effective. The existing evidence base, including the Cochrane systematic reviews, has focused on discrete two-way comparisons, with no attempt being made to collate all of the evidence on the surgical options available and rank them in terms of clinical effectiveness, safety and cost-effectiveness. This has resulted in a piecemeal evidence base that is difficult for women and clinicians to interpret. This assessment includes an evidence synthesis of all available randomized controlled trials to determine the relative clinical effectiveness and safety of interventions, a discrete choice experiment (DCE) to explore women’s preferences, an economic decision model to determine the most cost-effective treatment and a value-of-information (VOI) analysis to help inform the focus of further research.” (11)<br>**Example 4: In a review examining the effects of dietary inorganic nitrate for lowering blood pressure in hypertensive adults, the authors report what information the review seeks to add to current knowledge, and indicate that no systematic review addressing the same question exists:**<br>“…it is well known that the organic nitrates lower blood pressure in hypertensive individuals, which brings about the question of whether inorganic nitrates have the same ability. This review focuses on the dietary alteration component of lifestyle modifications by the use of inorganic nitrate in the treatment of hypertension. The appraisal of the evidence was completed to ultimately help providers make informed decisions regarding interventions to address one of the nation's biggest killers. There was a systematic review published in 2013 that addressed the effects of dietary inorganic nitrate on blood pressure with an overrepresentation of healthy, normotensive participants. That review found that inorganic nitrates decrease blood pressure. For this reason, this review examines studies published from 2013 through 2018 with blood pressure greater than 120/80 mmHg in participants, which would be considered elevated according to the guidelines published by the American College of Cardiology |

| Checklist item | Examples |
| --- | --- |
| | (ACC) and American Heart Association (AHA). The results of this review will contribute towards a greater understanding of possible treatments for hypertension, sequentially resulting in less morbidity and mortality from cardiovascular diseases. At the time of this systematic review, there was no systematic review that evaluated the effects of inorganic nitrate specifically on adults with blood pressure greater than 120/80 mmHg." (12) |
| Item 4. OBJECTIVES: Provide an explicit statement of the objective(s) or question(s) the review addresses. | **Example 1: In a review examining the effects of anti-tumour necrosis factor-blocking agents for rheumatoid arthritis, the authors report a single objective of the review:**<br><br>"Objectives: To evaluate the benefits and harms of down-titration (dose reduction, discontinuation, or disease activity-guided dose tapering) of anti-tumour necrosis factor-blocking agents (adalimumab, certolizumab pegol, etanercept, golimumab, infliximab) on disease activity, functioning, costs, safety, and radiographic damage compared with usual care in people with rheumatoid arthritis and low disease activity." (13)<br><br>**Example 2: In a review examining the effects of pre-exposure prophylaxis for the prevention of HIV infection, the authors report five key questions the review addresses:**<br><br>"Key Questions:<br>1. What are the benefits of PrEP in persons without pre-existing HIV infection versus placebo or no PrEP (including deferred PrEP) on the prevention of HIV infection and quality of life?<br>a. How do the benefits of PrEP differ by population subgroups?<br>b. How do the benefits of PrEP differ by dosing strategy or regimen?<br>2. What is the diagnostic accuracy of provider or patient risk assessment tools in identifying persons at increased risk of HIV acquisition who are candidates for PrEP?<br>3. What are rates of adherence to PrEP in U.S. primary care–applicable settings?<br>4. What is the association between adherence to PrEP and effectiveness for preventing HIV acquisition?<br>5. What are the harms of PrEP versus placebo or no PrEP when used for the prevention of HIV infection?" (14)<br><br>**Example 3: In a review examining the effects of mobile health interventions during the perinatal period for mothers in low- and middle-income countries, the authors report the primary objective of the review and specify two questions the review addresses:**<br><br>"The primary objective of this review was to determine the impact of mother-targeted mHealth educational interventions during the perinatal period in low- and middle-income countries on maternal and neonatal outcomes. Thus, this quantitative review aimed to answer the following questions: |

| Checklist item | Examples |
|---|---|
| | i. What is the impact of mother-targeted mHealth educational interventions on maternal knowledge, self-efficacy and antenatal/postnatal care clinic attendance in low- and middle-income countries?<br>ii. What is the impact of mother-targeted mHealth educational interventions on neonatal mortality and morbidity in low- and middle-income countries?" (15)<br><br>**Example 4: In a review examining the effects of screening for esophageal adenocarcinoma in patients with chronic gastroesophageal reflux disease, the authors report two key questions the review addresses:**<br><br>"In order to determine the effectiveness of screening for esophageal adenocarcinoma among gastroesophageal reflux disease patients, the following key questions were addressed:<br><br>1a. In adults (≥ 18 years) with chronic gastroesophageal reflux disease with or without other risk factors, what is the effectiveness (benefits and harms) of screening for esophageal adenocarcinoma and precancerous conditions (Barrett's Esophagus and low- and high-grade dysplasia)? What are the effects in relevant subgroup populations?"<br><br>1b. If there is evidence of effectiveness, what is the optimal time to initiate and to end screening, and what is the optimal screening interval (includes single and multiple tests and ongoing 'surveillance')?" (16) |
| Item 5. ELIGIBILITY CRITERIA: Specify the inclusion and exclusion criteria for the review and how studies were grouped for the syntheses. | **Example 1: In a review examining the effects of family therapy for people with anorexia nervosa, the authors report the types of studies, participants, interventions, comparators, and outcomes that were eligible for inclusion in the review, and state that there were no restrictions on the type of reports that were eligible (i.e. published or unpublished, any language, any date of publication):**<br><br>"Types of studies: We include all published or unpublished randomised controlled trials (RCTs). We would also have included cluster-randomised controlled trials and cross-over trials, but we found none. There were no language restrictions, nor did we exclude studies on the basis of the date of publication.<br><br>Types of participants: We included people of any age or gender with a primary clinical diagnosis of anorexia nervosa (AN), either or both purging or restricting subtypes, based on DSM (APA 2013) or ICD criteria (WHO 1992) or clinicians' judgement, and of any severity. We included those with chronic AN. We included those with psychiatric comorbidity, with the details of comorbidity documented. Participants may have received the intervention in any setting (including in-, day- or outpatient) and may have started in the trial at the beginning of treatment or part-way through (e.g. after discharge from hospital or some other indication/definition of stabilisation). We included those living in a family unit (of any nature, as described/defined by study authors), and those living outside of a family unit. |

| Checklist item | Examples |
| --- | --- |
| | Types of interventions: Trials where the intervention describes inclusion of the family in some way and is labelled 'family therapy'. These interventions may have been delivered as a monotherapy or in conjunction with other interventions (including standard care, which may or may not be in the context of an inpatient admission). The main categories of family therapy approaches considered were:<br>• Structural family therapy<br>• Systems (systemic) family therapy<br>• Strategic family therapy<br>• Family-based therapy and its variants (including short-term, long-term, and separated) and behavioural family systems therapy (these two therapies were grouped together, given the similarity of approach)<br>• Other (including other approaches that use family involvement in therapy but are less specific about the theoretical underpinning of the therapy and its procedures).<br>Family therapy approaches were compared with:<br>• Standard care or treatment as usual<br>• Biological interventions (for example, antidepressants, antipsychotics, mood stabilisers, anxiolytics, neutraceuticals, and other agents such as anti-glucocorticoids)<br>• Educational interventions (for example, nutritional interventions and dietetics)<br>• Psychological interventions (for example, cognitive behavioural therapy (CBT) and its derivatives, cognitive analytical therapy, interpersonal therapy, supportive therapy, psychodynamic therapy, play therapy, other)<br>• Alternative or complementary interventions (for example, massage, exercise, light therapies).<br>Additionally, different types of family therapy approaches were compared to each other. The addition of a family therapy approach to other interventions (including standard care) was also compared to other interventions alone. We would also have included the following comparisons: Family therapy approaches versus biological interventions; and Family therapy approaches versus alternative/complementary interventions; however, we had neither the relevant trials nor useable data from these.<br>Types of outcome measures: Primary outcomes included:<br>• Remission (by DSM or ICD or trialist-defined cut-off on standardised scale measure for remission versus no remission)<br>• All-cause mortality<br>Secondary outcomes included: |

| Checklist item | Examples |
| --- | --- |
| | • Family functioning as measured on standardised, validated and reliable measures, e.g. Family Environment Scale (Moos 1994), Expressed Emotions (Vaughn 1976), FACES III (Olson 1985)<br>• General functioning, measured by return to school or work, or by general mental health functioning measures, e.g. Global Assessment of Functioning (GAF) (APA 1994)<br>• Dropout (by rates per group during treatment)<br>• Eating disorder psychopathology (evidence of ongoing preoccupation with weight/shape/food/eating by eating-disorder symptom measures using any recognised validated eating disorders questionnaire or interview schedule, e.g. the Morgan-Russell Assessment Schedule (Morgan 1988), Eating Attitudes Test (EAT, Garner 1979), Eating Disorders Inventory (Garner 1983; Garner 1991).<br>• Weight, including all representations of this measure such as kilograms, body mass index (BMI, kg/m2) and average body weight (ABW) calculations. We included this measure after the finalisation of our protocol, due to the lack of universal reporting on remission, and the differing definitions used for remission<br>• Relapse (by DSM or ICD or trialist-defined criteria for relapse or hospitalisation)" (17)<br><br>**Example 2: In a review examining the effects of perioperative interventions for prevention of postoperative pulmonary complications, the authors report the types of studies, participants, interventions and outcomes that were eligible for inclusion in the review, indicating that studies were excluded if they measured outcomes that were neither patient centric nor clinically relevant.**<br><br>"Population: We included RCTs of adult (age ≥18 years) patients undergoing non-cardiac surgery, excluding organ transplantation surgery (as findings in patients who need immunosuppression may not be generalisable to others).<br><br>Intervention: We considered all perioperative care interventions identified by the search if they were protocolised (therapies were systematically provided to patients according to pre-defined algorithm or plan) and were started and completed during the perioperative pathway (that is, during preoperative preparation for surgery, intraoperative care, or inpatient postoperative recovery). Examples of interventions that we did or did not deem perioperative in nature included long term preoperative drug treatment (not included, as not started and completed during the perioperative pathway) and perioperative physiotherapy interventions (included, as both started and completed during the perioperative pathway). We excluded studies in which the intervention was directly related to surgical technique.<br><br>Outcomes: To be included, a trial had to use a defined clinical outcome relating to postoperative pulmonary complications, such as "pneumonia" diagnosed according to the Centers for Disease Control and Prevention's definition. RCTs reporting solely physiological (for example, lung volumes and flow measurements) or biochemical (for example, lung inflammatory markers) outcomes are valuable but neither patient centric nor necessarily clinically relevant, and we therefore excluded them. We applied |

| Checklist item | Examples |
|---|---|
| | no language restrictions. Our primary outcome measure was the incidence of PPCs, with PPCs being defined as the composite of any of respiratory infection, respiratory failure, pleural effusion, atelectasis, or pneumothorax…Where a composite PPC was not reported, we contacted corresponding authors via email to request additional information, including primary data." (18)<br><br>**Example 3: In a review examining the effects of pharmacological or non-pharmacological interventions for adults with exacerbation of chronic obstructive pulmonary disease, the authors report inclusion and exclusion criteria for participants, interventions, comparators, outcomes, settings, study designs, and language of publication. More detail is provided in a table.**<br><br>"The eligible studies had to meet all of the following criteria: 1) adult 18 years and older with exacerbations of chronic obstructive pulmonary disease (ECOPD); 2) received pharmacologic intervention or nonpharmacologic interventions; 3) compared with placebo, standard care, for antibiotics and systemic corticosteroids: different types of agents, different delivery modes, and different durations of treatments; 4) reported outcomes of interest; 5) conducted in outpatient, inpatients, and emergency department; 6) randomized controlled trials (RCTs); and 7) published in English. We excluded studies conducted in the intensive care unit, or chronic ventilator unit or respiratory care unit; studies of patients with exacerbation of chronic bronchitis if they did not have any evidence of airflow limitation on spirometry (at any time, including during a stable state); and studies of health service interventions (e.g. hospital in the home as alternative to hospitalization). We focused only on interventions during the initial acute phase of an exacerbation of COPD and not during the convalescence period. We did not restrict study location or sample size. The detailed inclusion and exclusion criteria are listed in Table 1. All outcomes were final health outcomes except for the intermediate outcome, "forced expiratory volume in one second" (FEV1). FEV1 was included because it is a commonly used outcome in COPD studies and has been shown to be highly predictive of final health outcomes during ECOPD (including mortality, need for intubation, or hospital admission for COPD)." (19) |
| Item 6. INFORMATION SOURCES: Specify all databases, registers, websites, organisations, reference lists and other sources searched or consulted to identify studies. Specify the date | **Example 1: In a review examining the effects of altering the availability or proximity of food, alcohol, and tobacco products to change their selection and consumption, the authors list the electronic bibliographic databases (with dates of coverage for each), trials registers and websites searched. They also indicate that reference lists of all eligible study reports were reviewed and forward citation tracking of all eligible study reports was conducted:**<br><br>"We conducted electronic searches for eligible studies within each of the following databases:<br><br>• Cochrane Central Register of Controlled Trials (CENTRAL) (1992 to 23rd July 2018);<br>• MEDLINE (including MEDLINE In-Process) (OvidSP) (1946 to 23rd July 2018);<br>• Embase (OvidSP) (1980 to 23rd July 2018); |

| Checklist item | Examples |
|---|---|
| when each source was last searched or consulted. | • PsycINFO (OvidSP) (1806 to 23rd July 2018);<br>• Applied Social Sciences Index and Abstracts (ASSIA) (ProQuest) (1987 to 24th July 2018);<br>• Science Citation Index Expanded (Web of Science) (1900 to 24th July 2018);<br>• Social Sciences Citation Index (Web of Science) (1956 to 24th July 2018); and<br>• Trials Register of Promoting Health Interventions (EPPI Centre) (2004 to 27th July 2018).<br>We conducted electronic searches of the following grey literature databases using search strategies adapted from the final MEDLINE search strategy, as described above:<br>• Conference Proceedings Citation Index - Science (Web of Science) (1990 to 24th July 2018);<br>• Conference Proceedings Citation Index - Social Science & Humanities (Web of Science) (1990 to 24th July 2018); and<br>• OpenGrey (1997 to 24th July 2018).<br>We searched trial registers (US National Institutes of Health Ongoing Trials Register ClinicalTrials.gov (www.clinicaltrials.gov/), the World Health Organization International Clinical Trials Registry Platform (apps.who.int/trialsearch/), and the EU Clinical Trials Register (www.clinicaltrialsregister.eu/) to identify registered trials (up to 25th July 2018), and the websites of key organisations in the area of health and nutrition, including the following:<br>• UK Department of Health;<br>• Centers for Disease Control and Prevention (CDC), USA;<br>• World Health Organization (WHO);<br>• International Obesity Task Force; and<br>• EU Platform for Action on Diet, Physical Activity and Health.<br>In addition, we searched the reference lists of all eligible study reports and undertook forward citation tracking (using Google Scholar) to identify further eligible studies or study reports (up to 25th July 2018)" (20)<br>**Example 2: In a review examining the educational outcomes of children in contact with social care in England, the authors report the databases and other sources consulted, along with the date each source was searched:** |

| Checklist item | Examples |
|---|---|
| | "On 21 December 2017, MAJ searched 16 health, social care, education and legal databases, the names and date coverage of which are given in Table 2. […] We also carried out a 'snowball' search to identify additional studies by searching the reference lists of publications eligible for full-text review and using Google Scholar to identify and screen studies citing them. […] On 26 April 2018, we conducted a search of Google Scholar and additional supplementary searches for publications on websites of 10 relevant organisations (including government departments, charities, think-tanks and research institutes). Full details of these supplementary searches can be found in the Additional file 2. Finally, we updated the database search on 7 May 2019, and the snowball and additional searches on 10 May 2019 as detailed in Additional file 3. We used the same search method, except that we narrowed the searches to 2017 onwards." (21)<br><br>**Example 3: In a review examining the effects of environmental interventions to reduce the consumption of sugar-sweetened beverages, the authors report the databases, trials registers and websites searched (noting the date when each was searched) and indicate in an Appendix the reports for which forward and backward citation searching occurred:**<br><br>"We performed searches in the following databases:<br><br>• Health:<br>  ○ MEDLINE<br>  ○ Embase (Excerpta Medica dataBASE)<br>  ○ CENTRAL (Cochrane Central Register of Controlled Trials)<br>• Multidisciplinary:<br>  ○ Scopus<br>  ○ Google Scholar<br>  ○ Social Science Citation Index<br>• Public health, health promotion and occupational health databases:<br>  ○ BiblioMap (EPPI-Centre database of health promotion research)<br>  ○ TRoPHI (EPPI-Centre Trials Register of Promoting Health Interventions)<br>• Nutrition: |

| Checklist item | Examples |
| --- | --- |
| | ○ eLENA (WHO e-Library of Evidence for Nutrition Actions)<br>• Sources for grey literature:<br>○ openGrey (formerly openSIGLE)<br>• Unpublished studies:<br>○ ClinicalTrials.gov<br>○ ICTRP (International Clinical Trials Registry Platform)<br>• Databases with a regional focus:<br>○ LILACS<br>○ SciELO Citation Index<br>We used the Ovid search interface for MEDLINE, Embase and CENTRAL.<br>In addition, we searched the websites of key organisations in the area of health, health promotion and nutrition, including the following:<br>• EU platform for action on diet, physical activity and health (ec.europa.eu/health/ph_determinants/life_style/nutrition/platform/database/dsp_search.cfm).<br>• U.S. Centers for Disease Control and Prevention (www.cdc.gov/nutrition/data-statistics/sugar-sweetened-beverages-intake.html).<br>• Rudd Center for Food Policy and Obesity (www.uconnruddcenter.org/publications).<br>• Harvard TH Chan School of Public Health Obesity Prevention Source (www.hsph.harvard.edu/obesity-prevention-source).<br>• World Obesity (www.worldobesity.org/what-we-do/policy-prevention).<br>We handsearched reference lists of included studies and previously published reviews, and contacted the corresponding author of included studies and previously published reviews as well as the members of the Review Advisory Group to identify additional studies. We also conducted a citing studies search with Scopus, i.e. we searched for studies that have cited included studies and previously published reviews. The studies used for these forward and backward citation searches are provided in Appendix 6… |

| Checklist item | Examples |
|---|---|
| | We updated searches to 24 January 2018. We included ScieELO, Google Scholar, Open Grey and Bibliomap in our original search (conducted on 27 - 28 June 2016), but not in our 2018 search update." (22)<br><br>**Example 4: In a review examining factors associated with HPV vaccine uptake among American Indians and Alaska Natives in the USA, the authors present a table specifying for each database or other source consulted, the name of the source, interface/platform through which it was searched, coverage range, date when the search was executed, and a brief description of what the source included.**<br><br>"…we searched several resources to maximise the inclusion of all relevant studies. A list of sources that were searched with their brief description is presented in table 1." (23) |
| Item 7. SEARCH STRATEGY: Present the full search strategies for all databases, registers and websites, including any filters and limits used. | **Example 1: In a review examining the effects of onabotulinumtoxinA and mirabegron for overactive bladder, the authors report the full search strategy for MEDLINE and Embase, along with the list of terms used when searching three websites. The authors also describe the methods used to develop the search strategies. The following example is an unpublished extension of the information provided in Freemantle et al. (24)**<br><br>"MEDLINE(R) In-Process & Other Non-Indexed Citations and Ovid MEDLINE were searched via OvidSP. The database coverage was 1946 to present and the databases were searched on 29 August 2013.<br><br>1. Urinary Bladder, Overactive/<br>2. ((overactiv$ or over-activ$ or hyperactiv$ or hyper-activ$ or unstable or instability or incontinen$) adj3 bladder$).ti,ab.<br>3. (OAB or OABS or IOAB or IOABS).ti,ab.<br>4. (urge syndrome$ or urge frequenc$).ti,ab.<br>5. ((overactiv$ or over-activ$ or hyperactiv$ or hyper-activ$ or unstable or instability) adj3 detrusor$).ti,ab.<br>6. Urination Disorders/<br>7. exp Urinary Incontinence/<br>8. Urinary Bladder Diseases/<br>9. (urge$ adj3 incontinen$).ti,ab.<br>10. (urin$ adj3 (incontinen$ or leak$ or urgen$ or frequen$)).ti,ab.<br>11. (urin$ adj3 (disorder$ or dysfunct$)).ti,ab.<br>12. (detrusor$ adj3 (hyperreflexia$ or hyper-reflexia$ or hypertoni$ or hyper-toni$)).ti,ab.<br>13. (void$ adj3 (disorder$ or dysfunct$)).ti,ab.<br>14. (micturition$ adj3 (disorder$ or dysfunct$)).ti,ab.<br>15. exp Enuresis/ |

| Checklist item | Examples |
|---|---|
| | 16. Nocturia/<br>17. (nocturia or nycturia or enuresis).ti,ab.<br>18. or/1-17<br>19. (mirabegron or betmiga$ or myrbetriq$ or betanis$ or YM-178 or YM178 or 223673-61-8 or "223673618" or MVR3JL3B2V).ti,ab,rn.<br>20. exp Electric Stimulation Therapy/<br>21. Electric Stimulation/<br>22. ((sacral or S3) adj3 (stimulat$ or modulat$)).ti,ab.<br>23. (neuromodulat$ or neuro-modulat$ or neural modulat$ or electromodulat$ or electro-modulat$ or neurostimulat$ or neuro-stimulat$ or neural stimulat$ or electrostimulat$ or electro-stimulat$).ti,ab.<br>24. (InterStim or SNS).ti,ab.<br>25. ((electric$ or nerve$1) adj3 (stimulat$ or modulat$)).ti,ab.<br>26. (electric$ therap$ or electrotherap$ or electro-therap$).ti,ab.<br>27. TENS.ti,ab.<br>28. exp Electrodes/<br>29. electrode$1.ti,ab.<br>30. ((implant$ or insert$) adj3 pulse generator$).ti,ab.<br>31. ((implant$ or insert$) adj3 (neuroprosthe$ or neuro-prosthe$ or neural prosthe$)).ti,ab.<br>32. PTNS.ti,ab.<br>33. (SANS or Stoller Afferent or urosurg$).ti,ab.<br>34. (evaluat$ adj3 peripheral nerve$).ti,ab.<br>35. exp Botulinum Toxins/<br>36. (botulinum$ or botox$ or onabotulinumtoxin$ or 1309378-01-5 or "1309378015").ti,ab,rn.<br>37. or/19-36<br>38. 18 and 37<br>39. randomized controlled trial.pt.<br>40. controlled clinical trial.pt.<br>41. random$.ti,ab.<br>42. placebo.ti,ab.<br>43. drug therapy.fs.<br>44. trial.ti,ab.<br>45. groups.ab. |

| Checklist item | Examples |
|---|---|
| | 46. or/39-45<br>47. 38 and 46<br>48. animals/ not humans/<br>49. 47 not 48<br>50. limit 49 to english language<br><br>Embase was searched via OvidSP. The database coverage was 1974 to Aug 28 2013 and the databases were searched on 29 August 2013.<br><br>1. overactive bladder/<br>2. ((overactiv$ or over-activ$ or hyperactiv$ or hyper-activ$ or unstable or instability or incontinen$) adj3 bladder$).ti,ab.<br>3. (OAB or OABS or IOAB or IOABS).ti,ab.<br>4. (urge syndrome$ or urge frequenc$).ti,ab.<br>5. ((overactiv$ or over-activ$ or hyperactiv$ or hyper-activ$ or unstable or instability) adj3 detrusor$).ti,ab.<br>6. micturition disorder/<br>7. exp urine incontinence/<br>8. bladder disease/<br>9. (urge$ adj3 incontinen$).ti,ab.<br>10. (urin$ adj3 (incontinen$ or leak$ or urgen$ or frequen$)).ti,ab.<br>11. (urin$ adj3 (disorder$ or dysfunct$)).ti,ab.<br>12. detrusor dyssynergia/<br>13. (detrusor$ adj3 (hyperreflexia$ or hyper-reflexia$ or hypertoni$ or hyper-toni$)).ti,ab.<br>14. (void$ adj3 (disorder$ or dysfunct$)).ti,ab.<br>15. (micturition$ adj3 (disorder$ or dysfunct$)).ti,ab.<br>16. nocturia/<br>17. (nocturia or nycturia or enuresis).ti,ab.<br>18. or/1-17<br>19. mirabegron/<br>20. (mirabegron or betmiga$ or myrbetriq$ or betanis$ or YM-178 or YM178 or 223673-61-8 or "223673618" or MVR3JL3B2V).ti,ab,rn,tn.<br>21. exp electrostimulation therapy/<br>22. electrostimulation/<br>23. ((sacral or S3) adj3 (stimulat$ or modulat$)).ti,ab. |

| Checklist item | Examples |
|---|---|
| | 24. neuromodulation/<br>25. (neuromodulat$ or neuro-modulat$ or neural modulat$ or electromodulat$ or electro-modulat$ or neurostimulat$ or neuro-stimulat$ or neural stimulat$ or electrostimulat$ or electro-stimulat$).ti,ab.<br>26. (InterStim or SNS).ti,ab.<br>27. ((electric$ or nerve$1) adj3 (stimulat$ or modulat$)).ti,ab.<br>28. (electric$ therap$ or electrotherap$ or electro-therap$).ti,ab.<br>29. TENS.ti,ab.<br>30. exp electrode/<br>31. electrode$1.ti,ab.<br>32. ((implant$ or insert$) adj3 pulse generator$).ti,ab.<br>33. ((implant$ or insert$) adj3 (neuroprosthe$ or neuro-prosthe$ or neural prosthe$)).ti,ab.<br>34. PTNS.ti,ab.<br>35. (SANS or Stoller Afferent or urosurg$).ti,ab.<br>36. (evaluat$ adj3 peripheral nerve$).ti,ab.<br>37. botulinum toxin/<br>38. botulinum toxin A/<br>39. (botulinum$ or botox$ or onabotulinumtoxin$ or 1309378-01-5 or "1309378015").ti,ab,rn,tn.<br>40. or/19-39<br>41. 18 and 40<br>42. randomized controlled trial/<br>43. "randomized controlled trial (topic)"/<br>44. crossover procedure/<br>45. double blind procedure/<br>46. single blind procedure/<br>47. random$.ti,ab.<br>48. factorial$.ti,ab.<br>49. (crossover$ or cross-over$).ti,ab.<br>50. placebo$.ti,ab.<br>51. doubl$ blind$.ti,ab.<br>52. singl$ blind$.ti,ab.<br>53. assign$.ti,ab.<br>54. allocat$.ti,ab. |

| Checklist item | Examples |
|---|---|
| | 55. volunteer$.ti,ab.<br>56. trial.ti,ab.<br>57. groups.ab.<br>58. or/42-57<br>59. 41 and 58<br>60. (animal experiment/ or animal model/ or animal tissue/ or nonhuman/) not exp human/<br>61. 59 not 60<br>62. limit 61 to english language<br><br>Searches of two selected websites were undertaken as follows:<br><br>Source: US Food and Drug Administration (FDA)<br><br>Interface / URL: http://www.accessdata.fda.gov/scripts/cder/drugsatfda/index.cfm<br><br>Search date: 09/09/13<br><br>The following terms were searched individually in the Drugs@FDA database [http://www.accessdata.fda.gov/scripts/cder/drugsatfda/index.cfm].<br><br>• mirabegron<br>• betmiga<br>• myrbetriq<br>• betanis<br>• botox<br>• botulinum<br>• onabotulinumtoxin<br>• onabotulinumtoxinA<br><br>The following terms were searched individually in the Medical Devices section of the FDA website using the search engine at: http://www.fda.gov/MedicalDevices/default.htm.<br><br>• Sacral nerve stimulation<br>• Sacral neurostimulation<br>• SNS |

| Checklist item | Examples |
|---|---|
| | • Tibial nerve stimulation<br>• Tibial neurostimulation<br>• PTNS<br><br>Source: Canadian Agency for Drugs and Technologies in Health (CADTH)<br><br>Interface / URL: http://www.cadth.ca/en/search?q=<br><br>Search date: 09/09/13<br><br>The following terms were searched individually using the CADTH site search engine.<br><br>• Bladder<br>• overactive<br>• mirabegron<br>• betmiga<br>• myrbetriq<br>• betanis<br>• botox<br>• botulinum<br>• onabotulinumtoxin<br>• onabotulinumtoxinA<br>• "Sacral nerve stimulation"<br>• neurostimulation<br>• SNS<br>• "tibial nerve stimulation"<br>• PTNS<br><br>Five known relevant studies were used to identify records within databases. Candidate search terms were identified by looking at words in the titles, abstracts and subject indexing of those records. A draft search strategy was developed using those terms and additional search terms were identified from the results of that strategy. Search terms were also identified and checked using the PubMed PubReMiner word frequency analysis tool. The MEDLINE strategy makes use of the Cochrane RCT filter reported in the Cochrane Handbook v5.2. The RCT filter used in the Embase search was developed by the authors. Animal studies are removed from MEDLINE by using a standard algorithm and from Embase using an approach that uses animal-related subject headings but |

| Checklist item | Examples |
|---|---|
| | excluding records that are also indexed with the Emtree heading 'Human'. As per the eligibility criteria the strategy was limited to English language studies.<br><br>The search strategy was validated by testing whether it could identify the five known relevant studies and also three further studies included in two systematic reviews identified as part of the strategy development process. All eight studies were identified by the search strategies in MEDLINE and Embase.<br><br>The strategy was developed by an information specialist and the final strategies were peer reviewed by an experienced information specialist within our team. Peer review involved proofreading the syntax and spelling and overall structure, but did not make use of the PRESS checklist.<br><br>Three additional approaches were used to identify further studies. The reference lists of the eligible trials were screened, the included studies of two recent systematic reviews were screened and a forward citation search of the eligible trials to identify publications that had cited them was conducted using Web of Science on 3 Dec 2013. (24)<br><br>**Example 2: In a review examining the effects of environmental interventions to reduce the consumption of sugar-sweetened beverages, the authors report the full search strategy for all databases searched. The following is an excerpt of how they reported the search strategy for trials registers:**<br><br>"For clinicaltrials.gov we used the advanced search interface, and used the search syntax "(sugar-sweetened beverage) OR SSB OR soda" to run searches in the following fields:<br><br>• Condition or disease<br>• Other terms<br>• Intervention/treatment<br>• Title/Acronym<br>• Outcome Measure<br><br>The search yielded 646 records, which we collated and de-duplicated in MS Excel. After de-duplication, 282 unique records remained.<br><br>For the International Clinical Trials Registry Platform (ICTRP) we used the advanced search interface, and used the search syntax "sugar-sweetened beverage OR SSB OR soda" to run searches in the following fields (with synonyms, all recruitment status):<br><br>• Title<br>• OR Condition |

| Checklist item | Examples |
|---|---|
| | • OR Intervention<br><br>The search resulted in 171 hits.<br><br>Based on the search, we identified two completed studies eligible for inclusion in our review (Collins 2016 SNAP; Collins 2016 WIC), which we found through clinicaltrials.gov. Moreover, we identified 10 ongoing studies which we judged likely to meet our eligibility criteria upon completion. We present details of these in Characteristics of ongoing studies. We found eight of these through our search in clincialtrials.gov, and two through our search in the ICTRP. We ran trial register searches on 21 June 2018." (22) |
| Item 8. SELECTION PROCESS: Specify the methods used to decide whether a study met the inclusion criteria of the review, including how many reviewers screened each record and each report retrieved, whether they worked independently, and if applicable, details of automation tools used in the process. | **Example 1: In a review examining the key components of shared decision-making models, the authors report piloting, double screening, and consensus methods for study selection:**<br><br>"Three researchers (AP, HB-R, FG) independently reviewed titles and abstracts of the first 100 records and discussed inconsistencies until consensus was obtained. Then, in pairs, the researchers independently screened titles and abstracts of all articles retrieved. In case of disagreement, consensus on which articles to screen full-text was reached by discussion. If necessary, the third researcher was consulted to make the final decision. Next, two researchers (AP, HB-R) independently screened full-text articles for inclusion. Again, in case of disagreement, consensus was reached on inclusion or exclusion by discussion and if necessary, the third researcher (FG) was consulted." (25)<br><br>**Example 2: In a review examining the long-term effects of alcohol consumption on cognitive function, the authors report piloting, single screening titles/abstracts, partial single screening of full-text, and linking reports to studies:**<br><br>"Citations identified from the literature searches and reference list checking were imported to EndNote and duplicates were removed. Three reviewers independently screened a sample of 109 citations to pre-test and refine coding guidance based on the inclusion criteria. Disagreements about eligibility were resolved through discussion. One reviewer (SB, JR, or SM) then each screened about a third of the remaining citations (grouped by year of publication) for inclusion in the review using the pre-tested coding guidance.<br><br>Full-text of all potentially eligible studies were retrieved. A sample of full-text studies was independently screened by two reviewers (SB and JR) until concordance was achieved (~15%; 37/228 of full-text studies screened). The remaining full-text studies were screened by one reviewer (SB or JR). All included studies, and those for which eligibility was uncertain, were screened by a second reviewer (JR or SB). Disagreements or uncertainty about eligibility were resolved through discussion, with advice from the review biostatisticians (JM, AF, or both) to confirm eligibility based on study design and analysis methods. Further information was sought from the authors of two studies (Piumatti 2018, Wardzala 2018) to clarify methods and interpretation of the analysis. |

| Checklist item | Examples |
|---|---|
| | Citations that did not meet the inclusion criteria were excluded and the reason for exclusion was recorded at the full-text screening. Cohort names, author names, and study locations, dates and sample characteristics were used to identify multiple reports arising from the same study (deemed to be a 'cohort'). These reports were matched and data extracted only from the report that provided the most relevant analysis and complete information for the review. In most cases, the decision was based on the outcome reported (global function was prioritised)." (26) |
| | **Example 3: In a review examining the effects of altering the availability or proximity of food, alcohol, and tobacco products to change their selection and consumption, the authors report priority screening methods and how non-English language articles were handled:** |
| | "We imported titles and abstracts retrieved by the searches into EPPI Reviewer v.4.10.2 (ER4) systematic review software. Duplicate records were identified, manually reviewed and then removed using ER4's automatic de-duplication feature, with the similarity threshold initially set to 0.85 and then to 0.80. Due to the large number of records retrieved, we developed a semi-automated screening workflow in ER4 that used machine learning to assign title-abstract records for duplicate manual screening. This workflow was designed to maximise the recall of eligible studies while reducing the overall screening workload to match the resources available. We planned for duplicate manual screening to apply to up to a third of records retrieved. |
| | In developing the workflow, we first screened a random sample of 500 title-abstract records to calculate inter-rater reliability and establish an initial estimate of the baseline inclusion rate (sample sized determined as per Shemilt 2014). Secondly, title-abstract records were prioritised for manual screening using active learning to distinguish between relevant and irrelevant records in conjunction with manual user input. This phase of the workflow stopped when each review author had completed 15 hours of duplicate screening without identifying any further potentially eligible studies. In practice, this equated to 1700 title-abstract records. |
| | When we found non-English language articles, we used Google Translate in the first instance to determine potential eligibility. We intended that if an article appeared to be eligible, we would have the article translated by a native language speaker or professional translation service, however no articles needed translating." (20) |
| | **Example 4: The following is a made-up example showing how to report use of machine learning and crowdsourcing in the study selection process:** |
| | Study selection followed a three-stage process that involved machine learning classifiers, crowdsourcing and manual screening. After removing duplicates, we applied Cochrane's RCT machine learning classifier (Thomas 2020) and removed from further consideration any record classified as highly unlikely to report a randomized trial (i.e. below the externally calibrated recall threshold of 99%). Records that remained were then screened by Cochrane Crowd (Noel-Storr 2020), a crowdsourcing platform |

| Checklist item | Examples |
|---|---|
| | that has consistently shown to be over 99% accurate. In Cochrane Crowd, every record is screened by at least two crowd members, with all disagreements resolved by two expert screeners. Records rejected by the crowd were removed from further consideration. Finally, records the crowd deemed likely to be reports of randomized trials were screened independently by two members of the review team in Covidence. [Example drafted by Steve McDonald and James Thomas, March 2020] |
| Item 9. DATA COLLECTION PROCESS: Specify the methods used to collect data from reports, including how many reviewers collected data from each report, whether they worked independently, any processes for obtaining or confirming data from study investigators, and if applicable, details of automation tools used in the process. | **Example 1: In a review examining the effects of pharmacological interventions for promoting smoking cessation during pregnancy, the authors report using a data collection form, the number of authors collecting data from studies and the process for resolving disagreements, and indicate that study authors were contacted if any data were unclear:**<br>"We designed a data extraction form based on that used by Lumley 2009, which two review authors (RC and TC) used to extract data from eligible studies. Extracted data were compared, with any discrepancies being resolved through discussion. RC entered data into Review Manager 5 software (Review Manager 2014), double checking this for accuracy. When information regarding any of the above was unclear, we contacted authors of the reports to provide further details." (27)<br>**Example 2: In a review examining the effects of pharmacological or non-pharmacological interventions for adults with exacerbation of chronic obstructive pulmonary disease, the authors report using a standardized form that was pilot tested, and indicate that independent reviewers extracted data, which was checked by another reviewer:**<br>"We developed a standardized data extraction form to extract study characteristics...The standardized form was pilot-tested by all study team members using five randomly selected studies. Reviewers worked independently to extract study details. A third reviewer reviewed data extraction, and resolve conflicts." (19)<br>**Example 3: In a review examining the association between smoking and sickness absence, the authors report using a standardized form that was pilot tested, that one reviewer extracted data which was checked by another reviewer, and that study authors were contacted:**<br>"A data extraction sheet was developed, pilot tested on ten randomly selected included articles and then refined. After finalizing the data extraction sheet, one reviewer performed the initial data extraction for all included articles and a second reviewer checked all proceedings…. Corresponding authors were asked for additional information in cases where data provided in the published articles were insufficient." (28) |
| Item 10a. DATA ITEMS: List and define all outcomes for which data were | **Example 1: In a review examining the long-term effects of alcohol consumption on cognitive function, the authors list and define the outcomes for which data were sought (e.g. cognitive function), and specify the decision rules used to decide which results to collect when multiple were available in studies (e.g. when multiple measures, time points and unadjusted and adjusted analyses were available):** |

| Checklist item | Examples |
|---|---|
| sought. Specify whether all results that were compatible with each outcome domain in each study were sought (e.g. for all measures, time points, analyses), and if not, the methods used to decide which results to collect. | "Eligible outcomes were broadly categorised as follows:<br><br>Cognitive function<br><br>• Global cognitive function<br>• Domain-specific cognitive function (especially domains that reflect specific alcohol-related neuropathologies, such as psychomotor speed and working memory)<br><br>Clinical diagnoses of cognitive impairment<br><br>• Mild cognitive impairment (also referred to as mild neurocognitive disorders)<br><br>These conditions were 'characterised by a decline from a previously attained cognitive level'.<br><br>Major cognitive impairment (also referred to as major neurocognitive disorders; including dementia) was excluded.<br><br>We expected that definitions and diagnostic criteria would vary across studies, so we accepted a range of definitions as noted under 'Methods of outcome assessment' section. Table 1 provides an example of specific domains of cognitive function used in the diagnosis of mild and major cognitive impairment in the Diagnostic and Statistical Manual of Mental Disorders, Fifth Edition (DSM-5)).<br><br>*Method of outcome measurement*<br><br>Any measure of cognitive function was eligible for inclusion. The tests or diagnostic criteria used in each study should have had evidence of validity and reliability for the assessment of mild cognitive impairment, but studies were not excluded on this basis.<br><br>We anticipated that many different methods would be used to assess cognitive functioning across studies. These include the following.<br><br>Clinical diagnoses of<br><br>• Mild cognitive impairment using explicit criteria (e.g. National Institute on Aging and the Alzheimer's Association (United States; NIA-AA) criteria; any of the definitions of mild cognitive impairment described in Matthews et al. 2008)<br><br>Neuropsychological tests used to assess global cognitive function, for example the:<br><br>• Mini-Mental State Examination (MMSE) |

| Checklist item | Examples |
|---|---|
| | • Addenbrooke's Cognitive Examination-Revised (ACE-R) which "incorporates the MMSE and assesses attention, orientation, fluency, language, visuospatial function, and memory, yielding subscale scores for each domain"<br>• Montreal Cognitive Assessment (MOCA), which provides measures for specific cognitive abilities and may be more suitable for assessing mild cognitive impairment than the MMSE<br><br>Neuropsychological tests for assessing domain-specific cognitive function, for example, tests of:<br>• Attention and processing speed, for example, the Trail making test (TMT-A)<br>• Memory, for example, the Hopkins verbal learning test (HVLT-R; immediate, delay)<br>• Visuospatial ability, for example the Block design test<br>• Executive function, for example, the Controlled Oral Word Association Test (COWAT)<br><br>Results could be reported as an overall test score that provides a composite measure across multiple areas of cognitive ability (i.e. global cognitive function), sub-scales that provide a measure of domain-specific cognitive function or cognitive abilities (e.g. processing speed, memory), or both.<br><br>*Timing of outcome assessment*<br><br>Studies with a minimum follow-up of 6 months were eligible, a time frame chosen to ensure that studies were designed to examine more persistent effects of alcohol consumption. This threshold was based on previous reviews examining the association between long-term cognitive impairment and alcohol consumption (e.g. Anstey 2009 specified 12 months) and guidance from the Cochrane Dementia and Cochrane Improvement Group, which suggests a minimum follow-up of 9 months for studies examining progression from mild cognitive impairment to dementia. We deliberately specified a shorter period to ensure studies reporting important long-term effects were not missed.<br><br>No restrictions were placed on the number of points at which the outcome was measured, but the length of follow-up and number of measurement points (including a baseline measure of cognition) was considered when interpreting study findings and in deciding which outcomes were similar enough to combine for synthesis. Since long-term cognitive impairment is characterised as a decline from a previous level of cognitive function and implies a persistent effect, studies with longer-term outcome follow up at multiple time points should provide the most direct evidence.<br><br>*Selection of cognitive outcomes where multiple were reported* |

| Checklist item | Examples |
|---|---|
| | We anticipated that individual studies would report data for multiple cognitive outcomes. Specifically, a single study may report results:<br>• For multiple constructs related to cognitive function, for example, global cognitive function and cognitive ability on specific domains (e.g. memory, attention, problem-solving, language);<br>• Using multiple methods or tools to measure the same or similar outcome, for example reporting measures of global cognitive function using both the Mini-Mental State Examination and the Montreal Cognitive Assessment;<br>• At multiple time points, for example, at 1, 5, and 10 years.<br>Where multiple cognition outcomes were reported, we selected one outcome for inclusion in analyses and for reporting the main outcomes (e.g. for GRADEing), choosing the result that provided the most complete information for analysis. Where multiple results remained, we listed all available outcomes (without results) and asked our content expert to independently rank these based on relevance to the review question, and the validity and reliability of the measures used. Measures of global cognitive function were prioritised, followed by measures of memory, then executive function.<br>In the circumstance where results from multiple multivariable models were presented, we extracted associations from the most fully adjusted model, except in the case where an analysis adjusted for a possible intermediary along the causal pathway (i.e. post-baseline measures of prognostic factors (e.g. smoking, drug use, hypertension))" (26)<br>**Example 2: In a review examining the effects of shockwave therapy for rotator cuff disease, the authors list and define the outcomes for which data were sought (e.g. pain, function), and specify the decision rules used to decide which results to collect when multiple were available in studies (e.g. when multiple measures, time points and unadjusted and adjusted analyses were available):**<br>"We presented the major outcomes below in the 'Summary of findings' tables.<br>• Participant-reported pain relief of 30% or greater.<br>• Mean pain score, or mean change in pain score on VAS or Numerical Rating Scale (NRS) or categorical rating scale (in that order of preference).<br>• Disability or function.<br>• Composite endpoints measuring 'success' of treatment such as participants feeling no further symptoms.<br>• Quality of life.<br>• Number of participant withdrawals, for example, due to adverse events or intolerance to treatment.<br>• Number of participants experiencing any adverse event. |

| Checklist item | Examples |
| --- | --- |
| | We extracted outcome measures assessing benefits of treatment (e.g. pain, function, success, quality of life) at the time points:<br>• up to six weeks;<br>• greater than six weeks to three months (this was the primary time point);<br>• greater than three months to up to six months;<br>• greater than six months to 12 months;<br>• greater than 12 months.<br>If data were available in a trial at multiple time points within each of the above periods (e.g. at four, five and six weeks), we only extracted data at the latest possible time point of each period. We extracted adverse events, calcification resolution and treatment success at the end of the trial.<br>For a particular systematic review outcome there may be a multiplicity of results available in the trial reports (e.g. multiple scales, time points and analyses). To prevent selective inclusion of data based on the results, we used the following *a priori* defined decision rules to select data from trials.<br>• Where trialists reported both final values and change from baseline values for the same outcome, we extracted final values.<br>• Where trialists reported both unadjusted and adjusted values for the same outcome, we extracted unadjusted values.<br>• Where trialists reported data analysed based on the intention-to-treat (ITT) sample and another sample (e.g. per-protocol, as-treated), we extracted ITT-analysed data.<br>Where trials did not include a measure of overall pain but included one or more other measures of pain, for the purpose of combining data for the primary analysis of overall pain, we combined overall pain with other types of pain in the following hierarchy:<br>• overall or unspecified pain;<br>• pain at rest;<br>• pain with activity;<br>• daytime pain;<br>• night-time pain.<br>Where trials included more than one measure of disability or function, we extracted data from the one function scale that was highest on the following *a priori* defined list: |

| Checklist item | Examples |
|---|---|
| | • Shoulder Pain And Disability Index (SPADI);<br>• Shoulder Disability Questionnaire (SDQ);<br>• Constant score;<br>• Disabilities of the Arm, Shoulder and Hand (DASH);<br>• Health Assessment Questionnaire (HAQ);<br>• any other function scale.<br><br>Where trials included more than one measure of treatment success, we extracted data from the one function scale that was highest on the following *a priori* defined list:<br><br>• participant-defined measures of success, such as asking participants if treatment was successful;<br>• trialist-defined measures of success, such as a 30-point increase on the Constant Score." (29)<br><br>**Example 3: In a review examining the effects of strategies to improve the implementation of healthy eating, physical activity and obesity prevention policies, practices or programmes within childcare services, the authors report the following decision rules to select results when multiple were available in study reports (e.g. multiple time points, multiple outcome measures, change scores versus final values):**<br><br>"We reported measures of treatment effect from included studies that were adjusted for potential confounding variables over reported estimates that were not adjusted for potential confounding. Where studies used multiple follow-up periods, we used data from the final (most recent) study follow-up. We included data from the primary implementation outcome in meta-analyses. In instances where the authors of included studies did not identify a primary implementation outcome, we used the outcome on which the study sample size and power calculation was based. In its absence, for studies using score-based measures of implementation, and reporting total and subscale scores, we assumed the total score represented the primary implementation outcome. Otherwise, we attempted to calculate a relative effect size for each implementation outcome measure, rank these based on effect size and used the measure reporting the median effect size to include in any pooled analysis. We calculated the effect size by subtracting the change from baseline of the primary implementation outcome for the control or comparison group from the change from baseline in the experimental or intervention group. If data to enable calculation of the change from baseline were unavailable, we used the differences between groups post-intervention. For score-based measures, we calculated a standardised ('d') measure of effect size for each outcome to rank the effect size. Where there were an even number of implementation outcomes, one of the two measures at the median was randomly selected and used for inclusion in meta-analysis." (30) |

| Checklist item | Examples |
| --- | --- |
| | **Example 4: In a review examining the effects of, the authors report how the outcome domains were selected and decision rules used to select results from among multiple measurement instruments:**<br><br>"Twelve dementia care partners (nurses, allied health professionals, physicians, and a caregiver) selected our study outcomes (18) by independently ranking a group of commonly reported neuropsychiatric symptoms (for example, aggression, agitation, and sleep disturbances) in descending order of importance. The care partners selected change in aggression as our main outcome and change in agitation as our secondary outcome… For all of our NMAs, we preferentially abstracted a scale (e.g. Neuropsychiatry inventory (NPI) – agitation subscale, CMAI) reported by study authors before abstracting an individual aggressive or agitated behaviour (e.g. kicking, biting, screaming). Only in the case of our NMA for the outcome of overall agitation and aggression were there cases where study authors reported more than one scale for the same outcome (e.g. NPI-agitation subscale and CMAI). The CMAI was the most commonly reported scale for the outcome of overall agitation and aggression. The NPI-agitation subscale was the second most common scale for the outcome of overall agitation and aggression. Other scales were reported much less frequently. Therefore, the CMAI was always preferentially abstracted, where reported. If the CMAI was not reported, but the NPI-agitation subscale was reported, then it was preferentially abstracted before any other scales used to report the outcome of overall agitation and aggression." (31) |
| Item 10b. DATA ITEMS: List and define all other variables for which data were sought (e.g. participant and intervention characteristics, funding sources). Describe any assumptions made about any missing or unclear information. | **Example 1: In a review examining the long-term effects of alcohol consumption on cognitive function, the authors list and define all variables for which data were sought, including characteristics of the study design, exposure and comparator, and participants:**<br><br>"We extracted information relating to the characteristics of included studies and results as follows.<br><br>1. Study identifiers and characteristics of the study design<br><br>• Study references (multiple publications arising from the same study were matched to an index reference, which is the study from which results were selected for analysis or summary)<br>• Study or cohort name, location, and commencement date<br>• Study design (categorised as 'prospective cohort study', 'nested case-control study', or 'other' using the checklist of study design features developed by Reeves and colleagues)<br>• Funding sources and funder involvement in the study<br><br>2. Characteristics of the exposure and comparator groups<br><br>• Levels of alcohol consumption as defined in the study, including details of how consumption was measured and categorised, and information required to convert data for reporting and analysis |

| Checklist item | Examples |
|---|---|
| | ○ Qualitative descriptors of each category, if used (e.g. never or non-drinker, abstainer, former drinker, low/moderate/heavy consumption)<br>○ Upper and lower boundaries of each category (e.g. 1 to 29 g per day; 5.1 to 10 units per week based on a standard drink in the UK)<br>○ Group used as referent category (comparator) in analyses and how defined<br>○ Units of measurement (e.g. standard units of alcohol per day and definition of unit)<br>○ Method of collecting alcohol consumption data (e.g. retrospective survey involving recall of alcohol consumption over different periods of life; intake diaries to measure current alcohol consumption); time points at which exposure data were collected<br>○ Sample size for each exposure group at each measurement point and included in analysis; number lost to follow up [these data were used in the analysis and risk of bias assessment]<br>○ Any additional parameters used to derive each category or exposure measure (e.g. alcohol consumption at each drinking occasion; frequency of drinking; recall period)<br>• Patterns of exposure<br>○ Any additional data not listed above that characterises and quantifies different patterns of alcohol exposure (e.g. consumption on heaviest drinking day; diagnosis of an alcohol-use disorder such as dependence or harmful drinking, and the method of assessment; definition of other frequency-based categories used to characterise patterns of drinking such as occasional drinking or infrequent consumption)<br>○ Duration/length of exposure period at study baseline and follow-up (directly reported or data that can be used to calculate)<br>○ Age at commencement of drinking (initial exposure)<br><br>3. Characteristics of participants<br><br>• Age at baseline and follow up, sex, ethnicity, co-morbidities, socio-economic status (including education), use of licit or illicit drugs, family history of alcohol dependence<br>• Other characteristics of importance within the context of each study<br>• Eligibility criteria used in the study” (26)<br><br>**Example 2: In this review examining the effects of pharmacological, psychological, and non-invasive brain stimulation interventions for treating depression after stroke, the authors list and define all variables for which data were sought, including characteristics of the report, participants, study design and intervention:** |

| Checklist item | Examples |
|---|---|
| | “We collected data on:<br>• the report: author, year, and source of publication;<br>• the study: sample characteristics, social demography, and definition and criteria used for depression;<br>• the participants: stroke sequence (first ever vs recurrent), social situation, time elapsed since stroke onset, history of psychiatric illness, current neurological status, current treatment for depression, and history of coronary artery disease;<br>• the research design and features: sampling mechanism, treatment assignment mechanism, adherence, non-response, and length of follow up;<br>• the intervention: type, duration, dose, timing, and mode of delivery.” (32)<br>**Example 3: In this review examining the effects of caregiver involvement in interventions for improving children's dietary intake and physical activity behaviours, the authors report their assumption about ages of children when such information was not reported:**<br>“When trial authors reported child grade rather than age, we assumed the following age distributions: kindergarten, four to six years; first grade, five to seven years; second grade, six to eight years, third grade, seven to nine; fourth grade, 8 to 10; fifth grade 9 to 11; sixth grade, 10 to 12; seventh grade, 11 to 13; eighth grade, 12 to 14; ninth grade, 13 to 15; tenth grade, 14 to 16; eleventh grade, 15 to 17; and twelfth grade, 16 to 18.” (33) |
| Item 11. STUDY RISK OF BIAS ASSESSMENT: Specify the methods used to assess risk of bias in the included studies, including details of the tool(s) used, how many reviewers assessed each study and whether they worked independently, and if applicable, details of | **Example 1: In a review examining the effects of altering the availability or proximity of food, alcohol, and tobacco products to change their selection and consumption, the authors specify the risk of bias tool used, the domains of bias addressed by the tool, how many reviewers assessment each study and how an overall judgement was reached:**<br>“We assessed risk of bias in the included studies using the revised Cochrane ‘Risk of bias’ tool for randomised trials (RoB 2.0) (Higgins 2016a), employing the additional guidance for cluster-randomised and cross-over trials (Eldridge 2016; Higgins 2016b). RoB 2.0 addresses five specific domains: (1) bias arising from the randomisation process; (2) bias due to deviations from intended interventions; (3) bias due to missing outcome data; (4) bias in measurement of the outcome; and (5) bias in selection of the reported result. Two review authors independently applied the tool to each included study, and recorded supporting information and justifications for judgements of risk of bias for each domain (low; high; some concerns). Any discrepancies in judgements of risk of bias or justifications for judgements were resolved by discussion to reach consensus between the two review authors, with a third review author acting as an arbiter if necessary. Following guidance given for RoB 2.0 (Section 1.3.4) (Higgins 2016a), we derived an overall summary 'Risk of bias' judgement (low; some concerns; high) for each specific outcome, whereby the overall RoB for each study was determined by the highest RoB level in any of the domains that were assessed.” (20) |

| Checklist item | Examples |
|---|---|
| automation tools used in the process. | **Example 2: In a review examining the effects of red light camera interventions for reducing traffic violations and traffic crashes, the authors report the risk of bias domains they assessed, how each were rated, and how many reviewers performed assessments:**<br><br>"The expanded risk of bias analysis was based on six dimensions that focused on the design of the study, the analysis of the data, and the contents of the study report. These six dimensions, which conform to the requirements set forth by the UK Economic and Social Research Council (ESRC), are:<br><br>1. Selection and matching of intervention and control areas<br>2. Blinding of data collection and analysis<br>3. Pre- and postintervention data collection periods<br>4. Reporting of results<br>5. Control of confounders<br>6. Control of other potential sources of bias<br><br>See Appendix G for a list of the 17 specific criteria included in each dimension. Each individual criterion statement was scored on whether it was True, False, or Unclear and these were used to assess each study on whether it presented a high, low, or unclear risk of bias across the six domains.<br><br>Risk of bias assessment was performed independently by three review authors (E.G.C., S.K., and C.P.). For the studies identified in the previous review, the same three review authors independently assessed the risk of bias of the included studies. Any discrepancies were resolved by deferment to further review authors (R.S. and P.E.). All disagreements were resolved by consensus." (34) |
| Item 12. EFFECT MEASURES: Specify for each outcome the effect measure(s) (e.g. risk ratio, mean difference) used in the synthesis or presentation of results. | **Example 1: In a review examining the effects of psychological interventions to foster resilience in healthcare students, the authors report planning to use the risk ratio for dichotomous outcomes and the standardised mean difference for continuous outcomes:**<br><br>"We planned to analyse dichotomous outcomes by calculating the risk ratio (RR) of a successful outcome (i.e. improvement in relevant variables) for each trial…Because the included resilience-training studies used different measurement scales to assess resilience and related constructs, we used standardised mean difference (SMD) effect sizes (Cohen's d) and their 95% confidence intervals (CIs) for continuous data in pair-wise meta-analyses." (35) |

| Checklist item | Examples |
|---|---|
| | **Example 2: In a review comparing the effects of pars plana vitrectomy combined with scleral buckle with pars plana vitrectomy alone for giant retinal tear, the authors report using the risk ratio in the synthesis or presentation of results for dichotomous outcomes:**<br><br>"We estimated the risk ratio (RR) and its 95% confidence interval (CI) after surgery (pars plana vitrectomy combined with scleral buckle vs pars plana vitrectomy alone) for the following dichotomous outcomes with information obtained from the included studies.<br><br>• Primary surgical success.<br>• Second surgery for retinal reattachment.<br>• Development of adverse events such as retinal detachment recurrence, elevation of intraocular pressure above 21 mmHg, choroidal detachment, cystoid macular edema, macular pucker, proliferative vitreoretinopathy, progression of cataract in initially phakic eyes, and any other adverse events reported by included trials at any time from day one up to the last reported follow-up visit after surgery." (36)<br><br>**Example 3: In a review examining the effects of metformin for endometrial hyperplasia, the authors report using the hazard ratio or odds ratio in the synthesis or presentation of time-to-event (survival) outcomes"**<br><br>"For survival outcomes (e.g. regression of endometrial hyperplasia, recurrence of endometrial hyperplasia, progression to endometrial carcinoma), we planned to calculate hazard ratios if data were available. Otherwise, we would calculate rates at a set time point, using the Mantel-Haenszel odds ratio (OR) and the numbers of events in control and intervention groups." (37) |
| Item 13a. SYNTHESIS METHODS: Describe the processes used to decide which studies were eligible for each synthesis (e.g. tabulating the study intervention characteristics and comparing against the planned groups | **Example 1: In a review examining the effects of interventions to reduce homelessness, the authors report categorising the interventions delivered in the included studies according to four dimensions:**<br><br>"Given the complexity of the interventions being investigated, we attempted to categorize the included interventions along four dimensions: (1) was housing provided to the participants as part of the intervention; (2) to what degree was the tenants' residence in the provided housing dependent on, for example, sobriety, treatment attendance, etc.; (3) if housing was provided, was it segregated from the larger community, or scattered around the city; and (4) if case management services were provided as part of the intervention, to what degree of intensity. We created categories of interventions based on the above dimensions:<br><br>1. Case management only<br>2. Abstinence-contingent housing<br>3. Non-abstinence-contingent housing<br>4. Housing vouchers |

| Checklist item | Examples |
|---|---|
| for each synthesis (item #5)). | 5. Residential treatment with case management<br><br>Some of the interventions had multiple components (e.g. abstinence-contingent housing with case management). These interventions were categorized according to the main component (the component that the primary authors emphasized). They were also placed in separate analyses. We then organized the studies according to which comparison intervention was used (any of the above interventions, or usual services).” (38) |
| Item 13b. SYNTHESIS METHODS: Describe any methods required to prepare the data for presentation or synthesis, such as handling of missing summary statistics, or data conversions. | **Example 1: In a review examining the effects of interventions to reduce homelessness, the authors report methods used to calculate standard deviations from other statistics reported:**<br><br>“In cases where the means, number of participants and test statistics for t-test were reported, but not the standard deviations, and there was the opportunity to include results in a meta-analysis, we calculated standard deviations, assuming the same standard deviation for each of the two groups (intervention and control)” (38).<br><br>**Example 2: In a review examining the effects of interventions to reduce homelessness, the authors report methods used to combine intervention groups of multi-arm studies:**<br><br>“Where we were interested in an intervention and it was compared to two or more comparison interventions that were both considered to be within the realm of “usual services”, we combined the two comparison arms into one comparison group and compared the means of the combined control groups to the intervention for a given outcome (for Morse 1992). In one study we have combined two intervention arms that both employed slightly differing versions of an intervention (assertive community treatment) into one intervention group and compared that to the usual services comparison condition (for Morse 1997)” (38).<br><br>**Example 3: In a review examining the effects of food fortification with multiple micronutrients on health outcomes in the general population, the authors report estimating and imputing intra-cluster correlation coefficients for cluster-randomised trials:**<br><br>“We used cluster-adjusted estimates from cluster randomised controlled trials (c-RCTs) where available. If the studies had not adjusted for clustering, we attempted to adjust their standard errors using the methods described in the Cochrane Handbook for Systematic Reviews of Interventions (Higgins 2019), using an estimate of the intra-cluster correlation coefficient (ICC) derived from the trial. If the trial did not report the cluster-adjusted estimated or the ICC, we imputed an ICC from a similar study included in the review, adjusting if the nature or size of the clusters was different (e.g. households compared to classrooms). We assessed any imputed ICCs using sensitivity analysis.” (39) |

| Checklist item | Examples |
|---|---|
| | **Example 4: In a review examining the effects of manually-generated reminders delivered on paper on professional practice and patient outcomes, the authors report standardising the direction of effects across studies:**<br><br>"Some studies targeted quality problems that involve 'underuse', so that improvements in quality correspond to increases in the percentage of patients who receive a target process of care (for example, increasing the percentage of patients who receive the influenza vaccine). However, other studies targeted 'overuse', so that improvements correspond to reductions in the percentage of patients receiving inappropriate or unnecessary processes of care (for example, reducing the percentage of patients who receive antibiotics for viral upper respiratory tract infections). In order to standardise the direction of effects, we defined all process adherence outcomes so that higher values represented an improvement. For example, data from a study aimed at reducing the percentage of patients receiving inappropriate medications would be captured as the complementary percentage of patients who did not receive inappropriate medications. Increasing this percentage of patients for whom providers did not prescribe the medications would thus represent an improvement. Each outcome can then be interpreted as compliance with desired practice." (40) |
| Item 13c. SYNTHESIS METHODS: Describe any methods used to tabulate or visually display results of individual studies and syntheses. | **Example 1: In a review examining the effects of interventions to reduce ambient particulate matter air pollution on health, the authors report their chosen plot, along with a rationale:**<br><br>"… in line with the review protocol we synthesized evidence narratively as well as graphically using harvest plots. Harvest plots have been shown to be an effective, clear and transparent way to summarize evidence of effectiveness for complex interventions (Ogilvie 2008; Turley 2013). We created eight separate harvest plots, one for health outcomes and one for air quality outcomes for each intervention category." (41)<br><br>**Example 2: In a review examining the effects of transfers and vouchers on the use and quality of maternity care services, the authors report using albatross plots to present results of individual studies, along with a rationale:**<br><br>"Meta-analyses could not be undertaken due to the heterogeneity of interventions, settings, study designs and outcome measures. Albatross plots were created to provide a graphical overview of the data for interventions with more than five data points for an outcome. Albatross plots are a scatter plot of p-values against the total number of individuals in each study. Small p-values from negative associations appear at the left of the plot, small p-values from positive associations at the right, and studies with null results towards the middle. The plot allows p-values to be interpreted in the context of the study sample size; effect contours show a standardised effect size (expressed as relative risk—RR) for a given p-value and study size, providing an indication of the overall magnitude of any association. We estimated an overall magnitude of association from these contours, but this should be interpreted cautiously." (42) |

| Checklist item | Examples |
|---|---|
| | **Example 3: In a review examining the effects of altering the availability or proximity of food, alcohol, and tobacco products to change their selection and consumption, the authors describe using 'Summary of findings' tables to present the synthesis results:**<br><br>"We developed 'Summary of findings' tables using GRADEpro GDT. These tables comprise summaries of the estimated intervention effect and the number of participants and studies for each primary outcome, and include justifications underpinning GRADE assessments. We planned to present separate summary effect sizes and certainty of evidence ratings for food, alcohol, and tobacco products, and for availability and proximity interventions within each of these product types, but in practice no eligible alcohol or tobacco studies were identified. Results of random-effects meta-analyses are presented as SMDs with 95% CIs. To facilitate interpretation of these estimated effect sizes, we re-expressed them employing selected familiar metrics of selection or consumption using observational data from a population-representative sample." (20) |
| Item 13d. SYNTHESIS METHODS: Describe any methods used to synthesize results and provide a rationale for the choice(s). If meta-analysis was performed, describe the model(s), method(s) to identify the presence and extent of statistical heterogeneity, and software package(s) used. | **Example 1: In a review examining the effects of functional appliance treatment on the temporomandibular joint, the authors report their chosen meta-analysis model, along with a rationale, the between-study variance estimator used, methods used to quantify statistical heterogeneity, and the software packages used:**<br><br>"As the effects of functional appliance treatment were deemed to be highly variable according to patient age, sex, individual maturation of the maxillofacial structures, and appliance characteristics, a random-effects model was chosen to calculate the average distribution of treatment effects that can be expected. A restricted maximum likelihood random-effects variance estimator was used instead of the older DerSimonian-Laird one, following recent guidance. Random effects 95% predictions were to be calculated for meta-analyses with at least three studies to aid in their interpretation by quantifying expected treatment effects in a future clinical setting. The extent and impact of between-study heterogeneity were assessed by inspecting the forest plots and by calculating the tau-squared and the I-squared statistics, respectively. The 95% CIs (uncertainty intervals) around tau-squared and the I-squared were calculated to judge our confidence about these metrics. We arbitrarily adopted the I-squared thresholds of > 75% to be considered as signs of considerable heterogeneity, but we also judged the evidence for this heterogeneity (through the uncertainty intervals) and the localization on the forest plot…All analyses were run in Stata SE 14.0 (StataCorp, College Station, TX) by one author." (43)<br><br>**Example 2: In a review examining the effects of individual-level behavioural smoking cessation interventions tailored for disadvantaged socioeconomic position, the authors report their chosen meta-analysis model, along with a rationale, the between-study variance estimator used, methods used to quantify statistical heterogeneity, and the software packages used:**<br><br>"Diverse interventions, settings, and participants characterise the field of smoking cessation. We judged it likely that the included studies would show heterogeneity in treatment effect (the observed intervention effects being more different from each other |

| Checklist item | Examples |
| --- | --- |
| | than one would expect because of random error alone). As such, the assumptions of a fixed-effect meta-analysis (that all studies in the meta-analysis share a common overall effect size and that all factors that could influence the effect size are the same across studies), were unlikely to hold…In random-effects meta-analysis models (restricted maximum-likelihood method), we calculated pooled risk ratios (RRs) with 95% confidence intervals (CIs) for both socioeconomic-position-tailored and non-socioeconomic-position-tailored interventions as the weighted average of each individual study's estimated intervention effect. All computations were done on a log scale with the log RR, its variance, and standard error (SE), before exponentiating the summary effect for interpretation. We explored heterogeneity by observation of forest plots and use of the $\chi^2$ test to show whether observed differences in results were compatible with chance alone. We calculated $I^2$ statistics to examine the level of inconsistency across study findings…Analyses were done in the RStudio development environment version 1.1.463 using R version 3.5.2 and the metafor package." (44) |
| | **Example 3: In a review examining the effects of manually-generated reminders delivered on paper on professional practice and patient outcomes, the authors report calculating the median effect estimate and interquartile range across all included studies:** |
| | "We based our primary analyses upon consideration of dichotomous process adherence measures (for example, the proportion of patients managed according to evidence-based recommendations). In order to provide a quantitative assessment of the effects associated with reminders without resorting to numerous assumptions or conveying a misleading degree of confidence in the results, we used the median improvement in dichotomous process adherence measures across studies…With each study represented by a single median outcome, we calculated the median effect size and interquartile range across all included studies for that comparison." (40) |
| | **Example 4: In a review examining the effects of homeopathy, the authors report combining P values:** |
| | "The statistical approach used, therefore, was the combination of the significance levels (P values). The rationale for this choice is that all the trials explored the same broad question, i.e. "is homeopathic treatment efficacious?", even if, for individual trials, the question asked expressed more specific terms and focused on a given treatment of a particular disease. Thus, unlike in the conventional meta-analytical methods, the method used does not involve pooling the numerical estimates of treatment effect sizes obtained, in our case, in very different situations. Using this approach, the null hypothesis tested is that the effect of interest (in this case, the efficacy of homeopathic treatment) is not present in any of the trials considered. If the null hypothesis is rejected, we can conclude that in at least one trial there is a non-null effect…Thus, we used seven methods: the sum of logs, the sum of Z, the weighted sum of Z, the sum of t, the mean Z, the mean P, the count test and the logit procedure. We present the results obtained with the method that gave the most conservative (least optimistic) results. A two-sided approach was adopted because of the format of the tested hypothesis (i.e. the effect could be either "negative" or "positive")." (45) |

| Checklist item | Examples |
|---|---|
| Item 13e. SYNTHESIS METHODS: Describe any methods used to explore possible causes of heterogeneity among study results (e.g. subgroup analysis, meta-regression). | **Example 1: In a review examining the effects of individual-level behavioural smoking cessation interventions tailored for disadvantaged socioeconomic position, the authors report conducting meta-regression to explore possible causes of heterogeneity among study results, indicating the potential effect modifiers considered and how they were defined, and noted that these were pre-specified:**<br>"Given a sufficient number of trials, we used unadjusted and adjusted mixed-effects meta-regression analyses to assess whether variation among studies in smoking cessation effect size was moderated by tailoring of the intervention for disadvantaged groups. The resulting regression coefficient indicates how the outcome variable (log risk ratio (RR) for smoking cessation) changes when interventions take a socioeconomic-position-tailored versus non-socioeconomic-tailored approach. A statistically significant (p<0·05) coefficient indicates that there is a linear association between the effect estimate for smoking cessation and the explanatory variable. More moderators (study-level variables) can be included in the model, which might account for part of the heterogeneity in the true effects. We pre-planned an adjusted model to include important study covariates related to the intensity and delivery of the intervention (number of sessions delivered (above median vs below median), whether interventions involved a trained smoking cessation specialist (yes vs no), and use of pharmacotherapy in the intervention group (yes vs no). These covariates were included *a priori* as potential confounders given that programmes tailored to socioeconomic position might include more intervention sessions or components or be delivered by different professionals with varying experience. The regression coefficient estimates how the intervention effect in the socioeconomic-position-tailored subgroup differs from the reference group of non-socioeconomic-position-tailored interventions." (44)<br>**Example 2: In a review examining the effects of intensive LDL cholesterol-lowering treatment for the prevention of major vascular events, the authors report conducting subgroup analyses and meta-regression to explore possible causes of heterogeneity among study results, indicating the potential effect modifiers considered and how they were defined:**<br>"First, we assessed the association between absolute LDL cholesterol reduction (calculated as a difference in baseline minus last-measured achieved LDL cholesterol between the treatment groups) and the relative risk (RR) of major vascular events for statins, ezetimibe, and PCSK9 inhibitors. Second, we did analyses to establish the effect of a reduction of 1 mmol/L in LDL cholesterol on the RR of major vascular events, stratified into four groups with mean baseline LDL cholesterol concentrations of 2.60 mmol/L or less, 2.61–3.40 mmol/L, 3.41–4.10 mmol/L, and more than 4.10 mmol/L (the recommended LDL cholesterol thresholds for treatment initiation). Subgroups of trials that reported outcomes of patients with baseline LDL cholesterol less than 2.07 mmol/L (80 mg/dL) were also analysed to most closely approximate a mean baseline LDL cholesterol of 1.80 mmol/L (70 mg/dL; the LDL cholesterol threshold for treatment in high-risk patients in the ACC/AHA and CCS guidelines). Subgroups of trials that reported outcomes of patients by sex, presence or absence of diabetes, presence or absence of chronic kidney disease (defined as estimate glomerular filtration rate <60 mL/min per 1.73 m), and presence or absence of heart failure were also meta-analysed. Meta- |

| Checklist item | Examples |
|---|---|
| | regression analyses were done with the following covariates: baseline LDL cholesterol, extent of LDL cholesterol reduction, mean age, 10-year risk of atherosclerotic cardiovascular disease, and median duration of follow-up. Non-standardised and standardised analyses were done for each 1 mmol/L reduction in LDL cholesterol. We used a multivariable model including the same covariates and drug class... Heterogeneity of RRs were assessed using $I^2$ and Cochran's Q statistic was used to test for differences between subgroups." (46)<br><br>**Example 3: In a review examining the effects of psychological interventions for common mental disorders in women experiencing intimate partner violence in low-income and middle-income countries, the authors report conducting post-hoc subgroup analyses to explore possible causes of heterogeneity among study results, indicating the potential effect modifiers considered and how they were defined:**<br><br>"We anticipated that, in settings where intimate partner violence was sufficiently prevalent to be measured, female therapists might have been considered more culturally acceptable to female participants. We did post-hoc subgroup analyses to compare differences in standardised mean differences (dSMDs) of trauma-focused interventions versus generic psychological interventions, female-delivered interventions versus mixed gender-delivered interventions, novel treatments for low and middle income countries (LMICs) versus those with an established evidence base in high-income countries, and those asking only about recent (within the past 12 months) intimate partner violence versus lifetime intimate partner violence." (5) |
| Item 13f. SYNTHESIS METHODS: Describe any sensitivity analyses conducted to assess robustness of the synthesized results. | **Example 1: In a review examining the effects of vitamin D supplementation during pregnancy, the authors report conducting several sensitivity analyses:**<br><br>"We conducted sensitivity meta-analyses restricted to trials with recent publication (2000 or later); overall low risk of bias (low risk of bias in all seven criteria); and enrolment of generally healthy women (rather than those with a specific clinical diagnosis). To incorporate trials with zero events in both intervention and control arms (which are automatically dropped from analyses of pooled relative risks), we also did sensitivity analyses for dichotomous outcomes in which we added a continuity correction of 0.5 to zero cells." (47)<br><br>**Example 2: In a review examining the effects of omega-3, omega-6, and total dietary polyunsaturated fat for prevention and treatment of type 2 diabetes mellitus, the authors report conducting two sensitivity analysis and indicate that these were requested by the funders of the review:**<br><br>"At the request of the funders, we did an additional sensitivity analysis with respect to compliance. Our protocol stated an intention to subgroup by "recent publications"; we changed this to run a sensitivity analysis including publications before 2010 combined with all publications from 2010 onwards with a trials registry entry (even if published retrospectively). As our funders |

| Checklist item | Examples |
|---|---|
| | were particularly interested in effects within trials of at least 12 months, we also ran an analysis limiting to trials of at least 52 weeks' duration." (48) |
| Item 14. REPORTING BIAS ASSESSMENT: Describe any methods used to assess risk of bias due to missing results in a synthesis (arising from reporting biases). | **Example 1: In a review examining the effects of surgery for rotator cuff tears, the authors report using funnel plots to assess small-study effects, noting that publication bias is one of several reasons for any asymmetry detected. They also report comparing outcomes specified within and across reports of studies to assess outcome reporting bias:**<br>"To assess small-study effects, we planned to generate funnel plots for meta-analyses including at least 10 trials of varying size. If asymmetry in the funnel plot was detected, we planned to review the characteristics of the trials to assess whether the asymmetry was likely due to publication bias or other factors such as methodological or clinical heterogeneity of the trials. To assess outcome reporting bias, we compared the outcomes specified in trial protocols with the outcomes reported in the corresponding trial publications; if trial protocols were unavailable, we compared the outcomes reported in the methods and results sections of the trial publications." (49)<br>**Example 2: In a review examining the effects of interventions for seizures in catamenial (menstrual-related) epilepsy, the authors report assessing selective reporting bias in the included studies by comparing pre-specified with reported outcomes:**<br>"To assess selective reporting bias, we compared the measurements and outcomes planned by the original investigators during the trial with those reported within the published paper by checking the trial protocols (when available) against the information in the final publication. Where published protocols were not available and the trial authors did not provide an unpublished protocol upon request, we compared the methods and the results sections of the published papers. We also used our knowledge of the clinical area to identify where trial investigators had not reported commonly used outcome measures." (50)<br>**Example 3: In a review examining the association between quality of dietary fat and genetic risk of type 2 diabetes, the authors report using contour-enhanced funnel plots and a statistical test for funnel plot asymmetry to assess small-study effects, noting that publication bias is one of several reasons for any asymmetry detected:**<br>"Small study effects owing to potential publication bias, poor methodological quality in smaller studies, artefactual associations, true heterogeneity, or chance were evaluated by using contour-enhanced funnel plots alongside visual examination and statistical tests for asymmetry (Debray's test)." (51)<br>**Example 4: In a review examining the effects of testosterone treatment on depressive symptoms in men, the authors report using contour-enhanced funnel plots and adjusting the meta-analytic result to assess small-study effects, noting that publication bias is one of several reasons for any asymmetry detected:** |

| Checklist item | Examples |
|---|---|
| | "Small-study effects (e.g. publication bias) was checked by contour-enhanced funnel plots and adjusted for by obtaining a precision-effect estimate with standard error. Although precision-effect estimate with standard error tends to slightly underestimate the true association if the observed effects were generated by questionable research practices, simulations suggest that it provides the most precise estimates in the presence of residual effect heterogeneity and small-study effects." (52) |
| Item 15. CERTAINTY ASSESSMENT: Describe any methods used to assess certainty (or confidence) in the body of evidence for an outcome. | **Example 1: In a review examining the effects of Tai Chi for rheumatoid arthritis, the authors report using the GRADE approach for assessing certainty in the body of evidence, stating how many reviewers performed assessments, the domains considered, and software used to perform assessments:**<br>"Two people (AM, JS) independently assessed the certainty of the evidence. We used the five GRADE considerations (study limitations, consistency of effect, imprecision, indirectness, and publication bias) to assess the certainty of the body of evidence as it related to the studies that contributed data to the meta-analyses for the prespecified outcomes. We assessed the certainty of evidence as high, moderate, low, or very low. We considered the following criteria for upgrading the certainty of evidence, if appropriate: large effect, dose-response gradient, and plausible confounding effect. We used the methods and recommendations described in sections 8.5 and 8.7, and chapters 11 and 12, of the Cochrane Handbook for Systematic Reviews of Interventions. We used GRADEpro GDT software to prepare the 'Summary of findings' tables (GRADEpro GDT 2015). We justified all decisions to down- or up-grade the certainty of studies using footnotes, and we provided comments to aid the reader's understanding of the results where necessary." (53)<br>**Example 2: In a review examining the effects of implantable cardiac defibrillators for people with non-ischaemic cardiomyopathy, the authors report using standardised language to convey their certainty in the body of evidence for an outcome:**<br>"We reported our findings using the language suggested by Glenton and colleagues, focusing on the size of the effect and its clinical significance in relation to the certainty of the evidence on which the result is based (including the precision of the effect) (see Appendix 3)." (54)<br>**Example 3: In a review examining the effects of pharmacotherapy for the treatment of cannabis use disorder, the authors report assessing certainty in the body of evidence using the system developed by the Evidence-based Practice Center (EPC) program (established by the US Agency for Healthcare Research and Quality):**<br>"We classified the overall strength of evidence (SOE) for each outcome as high, moderate, low, or insufficient by using an established method that considers study quality, consistency of findings, directness of the comparisons, precision, and |

| Checklist item | Examples |
| --- | --- |
| | applicability (Berkman et al.). For findings with SOE greater than insufficient, we classified the direction of effect as "evidence of benefit," "no benefit" (that is, no difference from placebo or mixed findings), or "favors placebo."" (55) |
| Item 16a. STUDY SELECTION: Describe the results of the search and selection process, from the number of records identified in the search to the number of studies included in the review, ideally using a flow diagram. | **Example 1: In a review examining the effects of text message reminders for improving sun protection habits, the authors report results of the search and selection process in text and in a flow diagram:**<br><br>"We found 1,333 records in databases searching. After duplicates removal, we screened 1,092 records, from which we reviewed 34 full-text documents, and finally included six papers [cited]. Later, we searched documents that cited any of the initially included studies as well as the references of the initially included studies. However, no extra articles that fulfilled inclusion criteria were found in these searches (Fig 1)." (56)<br><br>**Example 2: In a review examining the effects of pharmacological interventions for heart failure in people with chronic kidney disease, the authors report results of the search and selection process in text and in a flow diagram; the diagram includes an additional box specifying the number of studies and participants contributing to each synthesis:**<br><br>"Our search of the Cochrane Kidney and Transplant specialised register identified 869 records. We identified an additional 78 records using other sources (reference lists of review articles, relevant studies, and clinical practice guidelines) – therefore a total of 947 records (n=176 studies) were identified. We excluded 61 studies (n=252 records), either due to a population other than heart failure (n=38 studies), a non-pharmacological intervention (n=5), follow-up shorter than three months (n=16), or a study design other than a RCT (n=2) (see Characteristics of excluded studies). Overall, 115 studies were eligible. Of these, three are ongoing and awaiting publication of primary data (PARAGON-HF 2018; RELAXAHF-2 2017; TMAC 2007) and will be included in a future update of this review. As a result, 112 studies were included in this review (Figure 1)." (57)<br><br>**Example 3: In an updated review examining the effects of non-menthol flavours in e-cigarettes on perceptions and use, the authors report results of the search and selection process in text and in a flow diagram; the diagram shows the flow of records in the original and updated searches:**<br><br>"A total of 3191 articles resulted from searching the four databases during the initial search (21 March 2018). After authors removed duplicates, 2822 articles remained for title and abstract review, including 14 articles identified through manual search of references. Two authors (CM and HMB) reviewed the titles and abstracts of all 2822 articles. A third author (SK) resolved any discrepancies. Following this step, two authors (CM and HMB) reviewed the full text of all 114 articles eligible for full-text screening. A third author (SK) resolved any discrepancies. Eighty articles were excluded for the following reasons: they did not have data on the specified outcomes (n=27), used qualitative methodologies (n=27), focused on a tobacco product other than e-cigarettes (n=12), were only focused on menthol flavour (n=2), was a duplicate (n=1) or were not peer-reviewed, did not include original data, did not include full-text or included only a conference abstract (n=11). Articles that addressed e-cigarettes from the |

| Checklist item | Examples |
|---|---|
| | original systematic review (n=17) were then added to the 34 articles identified from this current review, combining for a total of 51 articles included in the final analysis. The study selection processes, which approximate but do not exactly follow the Preferred Reporting Items for Systematic Reviews and Meta-Analyses (PRISMA) methodology, are illustrated in figure 1." (58)<br><br>**Example 4: In a review examining the effects of gabapentin for neuropathic pain, the authors present a flow diagram delineating the number records according to the information source from which they originated (i.e. databases, trials registers, or other "hidden" sources):**<br><br>See page 52 of Supplement B of the systematic review by Mayo-Wilson et al. (59), available at https://ars.els-cdn.com/content/image/1-s2.0-S0895435617307217-mmc1.pdf |
| Item 16b. STUDY SELECTION: Cite studies that might appear to meet the inclusion criteria, but which were excluded, and explain why they were excluded. | **Example 1: In a review examining the effects of drug-eluting balloon angioplasty versus uncoated balloon angioplasty for the treatment of in-stent restenosis of the femoropopliteal arteries, the authors cite excluded studies and provide reasons for their exclusion in a table.**<br><br>"We excluded seven studies from our review (Bosiers 2015; ConSeQuent; DEBATE-ISR; EXCITE ISR; NCT00481780; NCT02832024; RELINE), and we listed reasons for exclusion in the Characteristics of excluded studies tables. We excluded studies because they compared stenting in Bosiers 2015 and RELINE, laser atherectomy in EXCITE ISR, or cutting balloon angioplasty in NCT00481780 versus uncoated balloon angioplasty for in-stent restenosis. The ConSeQuent trial compared DEB versus uncoated balloon angioplasty for native vessel restenosis rather than in-stent restenosis. The DEBATE-ISR study compared a prospective cohort of patients receiving DEB therapy for in-stent restenosis against a historical cohort of diabetic patients. Finally, the NCT02832024 study compared stent deployment versus atherectomy versus uncoated balloon angioplasty alone for in-stent restenosis." (60)<br><br>**Example 2: In a review examining the effects of organised cervical cancer screening on cervical cancer mortality in Europe, the authors present citations of excluded studies and a table providing reasons for their exclusion, in the appendix:**<br><br>"Of the remaining 64 articles, 54 were excluded for a variety of other reasons (Fig. 1). Ultimately, this review included a total of ten studies. All included studies were present in the initial database search. Excluded articles are listed in appendix Table B including reasons for exclusion." Citations for the excluded articles are listed in Appendix E. (61) |
| Item 17. STUDY CHARACTERISTICS: Cite each included | **Example 1: In a review examining the association between aspirin use and fracture risk, the authors include a table presenting for each included study the citation, study design, country, sample size, setting, mean age, percentage of females, number of years follow-up, exposure details and outcomes assessed:** |

| Checklist item | Examples |
|---|---|
| study and present its characteristics. | "Of the 12 unique studies, three were prospective cohort studies,[15 18 22] three were case-control studies,[20 25 26] and six were cross-sectional studies[14 16 17 23 24 39] (table 1)." (62)<br><br>**Example 2: In a review examining the effects of antenatal corticosteroids for maturity of term or near term foetuses, the authors include a table presenting for each included study the citation, study location, study design, number of centres, duration of the study, percentage of participants lost to follow-up, number of participants, inclusion criteria, specific drug delivered and its dosage, the control, gestational age of participants at randomization, and how outcomes were defined:**<br><br>"Table 1⇓ shows the characteristics of the included clinical trials." (63)<br><br>**Example 3: In a review examining the effects of interventions to facilitate shared decision making to address antibiotic use for acute respiratory infections in primary care, the authors include a table presenting the components of interventions delivered in the included studies (e.g. materials delivered, who provided, how it was delivered):**<br><br>"A summary of the main intervention components is described using the items from the Template for Intervention Description and Replication (TIDieR) checklist (see Table 1)." (64) |
| Item 18. RISK OF BIAS IN STUDIES: Present assessments of risk of bias for each included study. | **Example 1: In a review examining the effects of altering the availability or proximity of food, alcohol, and tobacco products to change their selection and consumption, the authors present a table indicating the domain-specific and overall risk of bias judgement for each study result, and include justification for assessments in a file uploaded to a public repository:**<br><br>"We used the RoB 2.0 tool to assess risk of bias for each of the included studies. A summary of these assessments is provided in Table 1. In terms of overall risk of bias, there were concerns about risk of bias for the majority of studies (20/24), with two of these assessed as at high risk of bias (Musher-Eizenman 2010; Wansink 2013a). A text summary is provided below for each of the six individual components of the 'Risk of bias' assessment. Justifications for assessments are available at the following (http://dx.doi.org/10.6084/m9.figshare.9159824)" (20)<br><br>**Example 2: In a review examining the effects of intensity-modulated radiation therapy in curative-intent management of head and neck squamous cell carcinoma, the authors present a forest plot displaying risk of bias judgements for each study (represented as traffic lights):**<br><br>"Fig 2. Forest plot (including the risk of bias assessment) demonstrating significant reduction in the risk of acute grade 2 or worse xerostomia with intensity modulated radiation therapy (IMRT) compared to conventional techniques. Note comparable benefit of IMRT over two-dimensional radiotherapy (2D-RT) and three-dimensional radiotherapy (3D-RT) on subgroup analyses. https://doi.org/10.1371/journal.pone.0200137.g002" (65) |

| Checklist item | Examples |
|---|---|
| Item 19. RESULTS OF INDIVIDUAL STUDIES: For all outcomes, present, for each study: (a) summary statistics for each group (where appropriate) and (b) an effect estimate and its precision (e.g. confidence/credible interval), ideally using structured tables or plots. | **Example 1: In a review examining the effects of quadruple versus triple combination antiretroviral therapies for treatment naive people with HIV, the authors present forest plots showing summary statistics for each group and effect estimates with confidence intervals for each study:**<br><br>For an example of individual study results presented for a dichotomous outcome, see "Fig 2. Meta-analysis of comparative effects between quadruple and triple combination antiretroviral therapies (cART) as first line treatment for people with HIV, on undetectable HIV-1 RNA" (66)<br><br>For an example of individual study results presented for a continuous outcome, see "Fig 3. Meta-analysis of comparative effects between quadruple and triple combination antiretroviral therapies (cART) as first line treatment for people with HIV, on increase in CD4 T cell count (cells/μL)" (66)<br><br>**Example 2: In a review examining the blood pressure lowering efficacy of renin inhibitors for primary hypertension, the authors present forest plots showing summary statistics for each group and effect estimates with confidence intervals for each study, and indicate in a footnote the origin of particular data:**<br><br>See meta-analysis in Figure 3. The footnote to this forest plot states: "CSPP100A2308 study: the SBP reduction in the treatment and placebo group are reported from the CSR page 61. CSPP100A2405 study: the SD for all treatment groups are calculated from SEM reported on page 7 in the CSR". (67) |
| Item 20a. RESULTS OF SYNTHESES: For each synthesis, briefly summarise the characteristics and risk of bias among contributing studies. | **Example 1: In a review examining the effects of breastfeeding programs and policies on maternal health outcomes in developed countries, the authors summarise various characteristics of the studies contributing to a synthesis of the effects of Baby-Friendly Hospital Initiative interventions:**<br><br>"Twelve included studies (described in 13 publications) assessed the effectiveness of Baby-Friendly Hospital Initiative interventions. All focused on postpartum women enrolled from hospital wards or birth facilities soon after delivery. Studies were conducted in diverse country settings including the United States (two studies); Taiwan (two studies); and one each in the Republic of Belarus, Hong Kong, Czech Republic, Russia, Croatia, Brazil, United Kingdom (multiple regions), and Scotland. All studies focused on multiple hospitals (>4) or clusters of hospitals. The majority of studies focused on women giving birth between 2000 and 2009; two enrolled women in the late 1990s…One included study was an RCT, 10 were prospective cohort studies, and 1 was a single-group pre-post study…In terms of population characteristics, seven studies reported on maternal age and generally enrolled women in their 20s and 30s. Three studies (set in the United States and United Kingdom) reported on race; the percentage of nonwhite participants enrolled ranged from 3 to 47 percent. In the six studies reporting on the percentage of enrolled women who were primiparous, the range was 38 to 67 percent" (68) |

| Checklist item | Examples |
|---|---|
| | **Example 2: In a review on stroke prevention in patients with atrial fibrillation, the authors summarise various characteristics and the risk of bias in studies investigating tools for determining stroke risk:**<br><br>"Overall, 61 studies described in 83 publications investigated our included tools for determining stroke risk in patients with nonvalvular AF and met the other inclusion criteria for Key Question 1. The included studies explored tools in studies of diverse quality, design, funding, and geographical location. Forty-three included studies were of good quality or rated as low risk of bias, 11 of fair quality or rated as medium risk of bias, and 7 were of poor quality or rated as high risk of bias. Studies with increased risk of bias had potential limitations related to handling of missing data, length of follow up between groups, blinding of outcomes assessors, whether confounders were assessed with reliable measures, and whether potential outcomes were prespecified. The studies covered broad geographical locations with 32 studies conducted in UK or continental Europe, 18 exclusively in the United States, 3 studies exclusively conducted in Canada, and 7 multinational trials. There was one study that did not report geographic location of enrolment. Ten studies were supported solely by industry, 8 studies received solely government support, 6 studies were supported by non-government, non-industry organizations, 15 studies received funding from multiple sources including government, industry, non-government and non-industry, and 22 studies did not report funding or it was unclear. We identified 52 studies using observational study design (prospective and retrospective cohorts) while 9 studies were identified as randomized controlled trials (RCTs)." (69)<br><br>**Example 3: In a review examining the effects of antipsychotics for the prevention and treatment of delirium, the authors summarise various characteristics and the risk of bias in studies comparing delirium incidence between haloperidol and placebo groups:**<br><br>"Nine randomized controlled trials (RCTs) directly compared delirium incidence between haloperidol and placebo groups. These RCTs enrolled 3,408 patients in both surgical and medical intensive care and non-intensive care unit settings and used a variety of validated delirium detection instruments. Five of the trials were low risk of bias, three had unclear risk of bias, and one had high risk of bias owing to lack of blinding and allocation concealment. Intravenous haloperidol was administered in all except two trials; in those two exceptions, oral doses were given. These nine trials were pooled, as they each identified new onset of delirium (incidence) within the week after exposure to prophylactic haloperidol or placebo." (70)<br><br>**Example 4: In a review examining the effects of quadruple versus triple combination antiretroviral therapies for treatment naive people with HIV, the authors present a figure displaying the characteristics and risk of bias of studies alongside their results for several meta-analyses:**<br><br>See Graphical Overview for Evidence Reviews visual summary (66) |

| Checklist item | Examples |
|---|---|
| Item 20b. RESULTS OF SYNTHESES: Present results of all statistical syntheses conducted. If meta-analysis was done, present for each the summary estimate and its precision (e.g. confidence/credible interval) and measures of statistical heterogeneity. If comparing groups, describe the direction of the effect. | **Example 1: In a review examining the effects of aspirin for primary prevention of cardiovascular disease, the authors report for a meta-analysis of risk ratios the number of included studies and participants, summary estimate and its 95% confidence interval, the $I^2$ measure of inconsistency, and they translate the relative effect into absolute terms:**<br>"Twelve studies [*each study cited*], including a total of 159,086 patients, reported on the rate of major bleeding complications. Aspirin use was associated with a 46% relative risk increase of major bleeding complications (risk ratio 1.46; 95% CI, 1.30-1.64; p < 0.00001; $I^2$ = 31%; absolute risk increase 0.077%; number needed to treat to harm 1295; Fig 1)" (71)<br>**Example 2: In a review examining the effect of exercise programmes for ankylosing spondylitis, the authors report for a meta-analysis of mean differences the number of included studies and participants, summary estimate and its 95% confidence interval, the $I^2$ measure of inconsistency, and they translate the absolute effect into relative terms and describe the clinical importance of the result:**<br>"Physical function (BASFI, 0 to 10 scale; lower score indicates higher function): Seven studies (312 participants) found a reduction in physical function score with exercise versus no intervention at the end of the intervention (mean difference (MD) -1.3, 95% confidence interval (CI) -1.7 to -0.9); absolute risk difference 13% (95% CI 9% to 17%); relative change 32% (95% CI 23% to 42%); Analysis 1.1). The statistical heterogeneity was not important ($I^2$= 23%). There was no important clinical meaningful benefit." (72)<br>**Example 3: In a review examining the effects of strategies to improve the implementation of healthy eating, physical activity and obesity prevention policies, practices or programmes within childcare services, the authors report for a meta-analysis of standardised mean differences the number of included studies and participants, summary estimate and its 95% confidence interval, the $I^2$ measure of inconsistency, and they translate the result into units of a particular measurement scale:**<br>"Score-based measures of implementation were the most common continuous outcomes in studies comparing an implementation strategy with usual practice or minimal support control and were reported in 11 studies including nine randomised trials. Pooled analysis providing moderate-certainty evidence including all nine randomised trials with score-based measures of implementation [*each study cited*] reported an improvement (standardised mean difference 0.49; 95% confidence interval 0.19 to 0.79; $I^2$ = 54%; P < 0.001; participants = 495 services; equivalent to a mean difference of 0.88 on the Environment and Policy Assessment and Observation (EPAO) scale) favouring groups receiving implementation support strategies (Analysis 1.1)." (30)<br>**Example 4: In a review examining the effects of workplace interventions for reducing sitting at work, the authors report for a meta-analysis of mean differences the number of included studies, summary estimate and its 95% confidence interval, the $I^2$ measure of inconsistency and a prediction interval:** |

| Checklist item | Examples |
|---|---|
| | "Ten studies compared the effects of using a sit-stand desk with or without information and counselling to the effects of using a sit-desk [*each study cited*]. The pooled analysis showed that the sit-stand desk with or without information and counselling intervention reduced sitting time at work by on average 100 minutes per eight-hour workday (95% confidence interval -116 to -84, $I^2$ = 37%; Analysis 1.1)… Data presented by one study, Sandy 2016, did not allow for calculation of time spent in sitting time at work and therefore we did not include the study in the quantitative synthesis. The prediction interval for sitting time ranged from -146 to -54 minutes a day." (73)<br><br>**Example 5: In a review examining the effects of transfers and vouchers on the use and quality of maternity care services, the authors present an albatross plot which was used to synthesise results and describe how readers should interpret the findings:**<br><br>"The albatross plot for uptake of antenatal care is shown in Fig 2. Seven of the nine data points showed a positive association between conditional cash transfers and antenatal care uptake. Most of the studies were around the relative risk contour of 1.05, corresponding to about a 2-3 percentage point increase in uptake of antenatal care. However, the studies on Mi Familia Progresa, Programa de Asignación Familia and Program Keluarga Harapan all fall around the 1.05 relative risk contour, and have percentage point increases of 7.2-18.7, therefore a 10-percentage point increase might be a more reasonable estimate of the effect of conditional cash transfers on uptake of antenatal care; we considered this to be a large effect." (42) |
| Item 20c. RESULTS OF SYNTHESES: Present results of all investigations of possible causes of heterogeneity among study results. | **Example 1: In a review examining the effects of systemic corticosteroids for critically ill patients with COVID-19, the authors present results of several subgroup analyses, indicating for each the summary estimate and its precision for each subgroup, the estimate for the difference between subgroups and its precision, and the P value for interaction:**<br><br>"Among the 4 trials that recruited critically ill patients who were and were not receiving invasive mechanical ventilation at randomization, the association between corticosteroids and lower mortality was less marked in patients receiving invasive mechanical ventilation (ratio of ORs, 4.34 [95% CI, 1.46-12.91]; P = .008 based on within-trial estimates combined across trials); however, only 401 patients (120 deaths) contributed to this comparison…All trials contributed data according to age group and sex. For the association between corticosteroids and mortality, the OR was 0.69 (95% CI, 0.51-0.93) among 880 patients older than 60 years, the OR was 0.67 (95% CI, 0.48-0.94) among 821 patients aged 60 years or younger (ratio of ORs, 1.02 [95% CI, 0.63-1.65], P = .94), the OR was 0.66 (95% CI, 0.51-0.84) among 1215 men, and the OR was 0.66 (95% CI, 0.43-0.99) among 488 women (ratio of ORs, 1.07 [95% CI, 0.58-1.98], P = .84)." (74)<br><br>**Example 2: In a review examining the effects of community-based coordinating interventions in dementia care, the authors present results of several subgroup analyses, indicating for each the summary estimate and its precision for each subgroup and the P value for a test for subgroup differences:** |

| Checklist item | Examples |
|---|---|
| | "Interventions using a case manager with a nursing background showed a greater positive effect on caregiver quality of life compared to those that used other professional backgrounds (standardised mean difference = 0.94 versus 0.03, respectively; p < 0.001). Interventions that did not provide case managers with supervision showed greater effectiveness for reducing the percentage of patients that are institutionalised compared to those that provided supervision (odds ratio = 0.27 versus 0.96 respectively; p = 0.02). There was weak evidence that interventions using a lower caseload for case managers had greater effectiveness for reducing the number of patients institutionalised compared to interventions using a higher caseload for case managers (odds ratio = 0.23 versus 1.20 respectively; p = 0.08). There was little evidence that the other intervention components modify treatment effects (see Table 3)." (75)<br><br>**Example 3: In a review examining the effects of motor control stabilisation exercises in chronic nonspecific low back pain patients, the authors present results of several meta-regression analyses, indicating for each the regression coefficient and its confidence interval:**<br><br>"The results of the five meta-regressions…are highlighted in Table 5. The training duration, frequency, total trainings dose and training-to-sustainability ratio showed no impact on the effect size of the primary outcome pain. The PEDro sum score was negatively associated with the effect size; a study with a score-decrease of 1 point shows an increase in the effect size of .24. Fig 9 illustrates this association." (76)<br><br>**Example 4: In a review examining the effects of cannabinoid administration for pain, the authors present results of several meta-regression analyses, indicating for each the regression coefficient and its confidence interval, and using plots to visualise the relationships:**<br><br>"Meta-regression results revealed that, when controlling for other explanatory variables, drug administration conditions were linked with pain reduction among included studies, such that cannabinoids (whole-plant cannabis and whole-cannabis extracts) β = −0.43, 95% confidence interval (CI) (−0.62, −0.24), p < 0.05 (Figure 4), and synthetic cannabinoids (Dronabinol, Nabilone, and CT3) β = −0.39, 95% CI (−0.65, −0.14), p < 0.05 (Figure 4), performed better than placebo. Furthermore, meta-regression results showed that, when controlling for other explanatory variables, sample size was linked with pain reduction, β = 0.01, 95% CI (0.00, 0.01), p < 0.05, such that studies involving smaller samples tended to report greater pain reduction effects (Figure 4). There were no observed interactions between drug administration conditions and sample size. Finally, meta-regression results showed that, when controlling for other explanatory variables, sample sex composition was linked with a modest, however non-significant, effect, β = −0.64, 95% CI (−1.37, 0.09), p = 0.09, such that studies including more female participants tended to report greater pain reductions (Figure 5)." (77) |

| Checklist item | Examples |
|---|---|
| Item 20d. RESULTS OF SYNTHESES: Present results of all sensitivity analyses conducted to assess the robustness of the synthesized results. | **Example 1: In a review examining the effects of vitamin D supplementation during pregnancy, the authors present in an appendix tables showing the results of primary and sensitivity analyses for several meta-analyses:**<br><br>"The magnitude of the pooled effect remained relatively stable in sensitivity analyses (table S13 in appendix 10)" (47)<br><br>**Example 2: In a review examining the effects of quadruple versus triple combination antiretroviral therapies for treatment naive people with HIV, the authors report that results of several sensitivity analyses were consistent with results of primary meta-analyses:**<br><br>"Sensitivity analyses that removed studies with potential bias showed consistent results with the primary meta-analyses (risk ratio 1.00 for undetectable HIV-1 RNA, 1.00 for virological failure, 0.98 for severe adverse effects, and 1.02 for AIDS defining events; supplement 3E, 3F, 3H, and 3I, respectively). Such sensitivity analyses were not performed for other outcomes because none of the studies reporting them was at a high risk of bias. Sensitivity analysis that pooled the outcome data reported at 48 weeks, which also showed consistent results, was performed for undetectable HIV-1 RNA and increase in CD4 T cell count only (supplement 3J and 3K) and not for other outcomes owing to lack of relevant data. When the standard deviations for increase in CD4 T cell count were replaced by those estimated by different methods, the results of figure 3 either remained similar (that is, quadruple and triple arms not statistically different) or favoured triple therapies (supplement 2)." (66)<br><br>**Example 3: In a review examining the effects of operative treatment versus nonoperative treatment of Achilles tendon ruptures, the authors show in a table the results of primary and sensitivity analyses for two meta-analyses and present forest plots for sensitivity analyses in an appendix:**<br><br>"Table 3 shows the results of the secondary sensitivity analyses. Re-rupture rate was reported in 17 (59%) high quality studies – 10 randomized controlled trials and seven observational studies. The overall pooled effect showed that operative treatment was associated with a significant reduction in re-rupture rate compared with nonoperative treatment (risk difference 5.1%; risk ratio 0.44, 0.30 to 0.64; P<0.001; $I^2$=0%) (supplementary figure F). Re-rupture rate was reported in 14 studies (48%) with a study period after the year 2000 – six randomized controlled trials and eight observational studies. The overall pooled effect showed a significant reduction in re-rupture rate after operative treatment compared with nonoperative treatment (risk difference 0.9%; risk ratio 0.59, 0.42 to 0.83; P=0.002; $I^2$=10%) (supplementary figure G)." (78) |
| Item 21. REPORTING BIASES: Present assessments of risk of bias due to missing results (arising from | **Example 1: In a review examining the effects of latrepirdine for Alzheimer's disease, the authors report identifying four studies where investigators measured a particular outcome, yet results for the outcome were unavailable for three of the studies:**<br><br>"Clinical global impression of change was assessed in Doody 2008, NCT00912288, CONCERT and CONNECTION using the CIBIC-Plus. However, we were only able to extract results from Doody 2008 [because no results for CIBIC-Plus were reported in the |

| Checklist item | Examples |
| --- | --- |
| reporting biases) for each synthesis assessed. | other three studies]…The authors reported small but significant improvements on the CIBIC-Plus for 183 patients (89 on latrepirdine and 94 on placebo) favouring latrepirdine following the 26-week primary endpoint (MD -0.60, 95% CI -0.89 to -0.31, P < 0.001). Similar results were found at the additional 52-week follow-up (MD -0.70, 95% CI -1.01 to -0.39, P < 0.001). However, we considered this to be low quality evidence due to imprecision and reporting bias. Thus, we could not draw conclusions about the efficacy of latrepirdine in terms of changes in clinical impression.” (79)<br><br>**Example 2: In a review examining the effects of pharmacotherapy for social anxiety disorder, the authors used visual inspection of a contour-enhanced funnel plot to conclude that the asymmetry observed was likely due to publication bias:**<br><br>“There is evidence of possible funnel plot asymmetry providing data on response to short-term medication treatment, both for the SSRIs and all medications combined. Inspection of the contour enhanced funnel plots for the SSRIs (Figure 4) and all of the trials (Figure 5) suggests that this asymmetry is due to publication bias, as trials with less precise treatment response outcomes are more likely than their higher precision counterparts to be missing from regions of the plot representing statistically non-significant treatment effects. Egger regression tests quantitatively confirmed this visual impression, providing evidence of possible publication bias for all of the medication trials (t = 2.8226, df = 49, P = 0.0069) and for the SSRIs (t = 2.6426, df = 22, P = 0.015).” (80)<br><br>**Example 3: In a review examining the effects of bystander programs on the prevention of sexual assault among adolescents and college students, the authors used visual inspection of a contour-enhanced funnel plot to conclude that the asymmetry observed was unlikely to be due to publication bias:**<br><br>“To examine small study and publication bias we created a contour-enhanced funnel plot of the 11 effect sizes plotted against their standard errors (see Figure 32). Visual inspection of the funnel plot reveals an absence of adverse intervention effects. Given the absence of negative effects in the regions of statistical significance and non-significance, the results from this contour-enhanced funnel plot indicate a potential risk of publication bias. To further investigate the possibility of bias, we conducted an Egger test for funnel plot asymmetry. The results provided no significant evidence of small study effects (bias coefficient: 0.36; t: −0.60, p = .56)...With these collective findings, we therefore conclude that the meta-analysis results shown in Figure 31 are likely robust to any small study/publication bias.” (81) |
| Item 22. CERTAINTY OF EVIDENCE: Present assessments of certainty (or confidence) in the | **Example 1: In a review examining the effects of surgery for rotator cuff tears, the authors report their certainty in text, along with rationale for their judgement, and present a Summary of Findings table including certainty judgements for several outcomes:**<br><br>“Compared with non-operative treatment, low-certainty evidence indicates surgery (repair with subacromial decompression) may have little or no effect on function at 12 months. The evidence was downgraded two steps, once for bias and once for imprecision |

| Checklist item | Examples |
|---|---|
| body of evidence for each outcome assessed. | – the 95% CIs overlap minimal important difference in favour of surgery at this time point.” (49). The summary of findings table presents the same information as the text above, with footnotes explaining judgements.<br><br>**Example 2: In a review examining the effects of polyunsaturated fatty acids on patient-important outcomes in children and adolescents with autism spectrum disorder, the authors report their certainty in the abstract text and present Summary of Findings tables including certainty judgements for several outcomes:**<br><br>“Polyunsaturated fatty acids (PUFAs) were superior compared to placebo in reducing anxiety in individuals with autism spectrum disorder (standardised mean difference -1.01, 95% CI -1.86 to -0.17; very low certainty of evidence)…Summary of findings for the comparisons PUFAs versus placebo and PUFAs versus healthy diet are presented in Table 2 and Table 3.” (82) |
| Item 23a. DISCUSSION: Provide a general interpretation of the results in the context of other evidence. | **Example 1: In a review examining the effects of interventions to improve early grade literacy in Latin America and the Caribbean, the authors compare their findings with those observed in other relevant reviews:**<br><br>“Although we need to exercise caution in interpreting these findings because of the small number of studies, these findings nonetheless appear to be largely in line with the recent systematic review on what works to improve education outcomes in low- and middle-income countries of Snilstveit et al. (2012). They found that structured pedagogical interventions may be among the effective approaches to improve learning outcomes in low- and middle-income countries. This is consistent with our findings that teacher training is only effective in improving early grade literacy outcomes when it is combined with teacher coaching. The finding is also consistent with our result that technology in education programs may have at best no effects unless they are combined with a focus on pedagogical practices. In line with our study, Snilstveit et al. (2012) also do not find evidence for statistically significant effects of the one-laptop-per-child program. These results are consistent with the results of a meta-analysis showing that technology in education programs are not effective when not accompanied by parent or student training (McEwan, 2015). However, neither Snilstveit et al. (2012) nor McEwan (2015) find evidence for negative effects of the one-laptop-per-child program on early grade literacy outcomes.” (83)<br><br>**Example 2: In a review examining the effects of individualized funding interventions to improve health and social care outcomes for people with a disability, the authors describe how their review differs to the methods used in two previous reviews:**<br><br>“As outlined in the protocol, the authors were aware of only two previous systematic reviews prior to commencing this study (Carter Anand et al., 2012; Webber et al., 2014). In one sense, the eligibility criteria within the current study were broader and more inclusive; for example, Webber et al. limited their review to mental health users only. The need for a results refinement process further highlights the broad scope of the current review. In another sense, however, this review was more restrictive in terms of the quality of evidence. To this end, quantitative studies were excluded if they were not designed to robustly evaluate |

| Checklist item | Examples |
|---|---|
| | effectiveness or did not have a control group, while previous reviews included studies without control groups (for example). Therefore, the studies included in this review are very different, in some respects from those captured in the above reviews. At the same time, however, the findings from this review were consistent in many respects with the two reviews previously identified." (84)<br><br>**Example 3: In a review examining the effects of Alcoholics Anonymous and other 12-step programs for alcohol use disorder, the authors compare the current review with the previous of the review and with other reviews and studies:**<br><br>"The evidence contained in this review is similar to, and extends that of the prior Cochrane Review (Ferri 2006b), which this review updates and replaces, as well as of other narrative reviews which found overall positive effects for AA/TSF interventions (e.g. Kaskutas 2009a; Kelly 2003b). The results presented in this review are also supported by other published analyses. One study from Project MATCH (Longabaugh 1998), found that regardless of whether outpatients' pre-treatment network was supportive or unsupportive of alcohol use at treatment intake, AA/TSF participants were more likely to be involved with AA, which in turn, subsequently explained the observed lower drinks per drinking day (DDD) and greater PDA advantages for TSF-treated participants observed at the 36-month follow-up. The prior Cochrane Review contained eight studies with 3417 participants (Ferri 2006b), and found that on the whole, AA/TSF interventions were as effective, but not more effective, than the interventions to which they were compared. This new review is based on 27 studies reported in 36 articles and has a total of 10,565 participants. It is considerably larger, comprises more rigorous studies, and found that, compared to other active psychosocial interventions for AUD, AA/TSF interventions often produce greater abstinence – notably continuous abstinence – as well as some reductions in drinking intensity, fewer alcohol-related consequences, and lower alcohol addiction severity. This review also included economic analyses, which augments prior reviews and adds important information regarding the cost-benefits of providing AA/TSF in clinical settings." (85) |
| Item 23b. DISCUSSION: Discuss any limitations of the evidence included in the review. | **Example 1: In a review examining the association between marijuana use and risk of cancer, the authors describe various limitations of the included studies:**<br><br>"Study populations were young, and few studies measured longitudinal exposure. The included studies were often limited by selection bias, recall bias, small sample of marijuana-only smokers, reporting of outcomes on marijuana users and tobacco users combined, and inadequate follow-up for the development of cancer… Most studies poorly assessed exposure, and some studies did not report details on exposure, preventing meta-analysis for several outcomes." (86)<br><br>**Example 2: In a review examining indicators associated with job morale among physicians and dentists in low-income and middle-income countries, the authors describe the limited applicability of the conclusions to people in low and middle income countries:** |

| Checklist item | Examples |
| --- | --- |
| | "…despite the use of a comprehensive search strategy, almost all included studies were from middle-income countries, possibly reflecting the shortage of resources for such studies in low-income countries. This means that our findings cannot be generalized to low-income countries. Also, relatively fewer findings were available from Africa, Southern Europe, and Central, Southern, and Southeastern Asia, which made it challenging to generalize conclusions about low and middle income countries." (87) |
| Item 23c. DISCUSSION: Discuss any limitations of the review processes used. | **Example 1: In a review examining the effect of quarantine alone or in combination with other public health measures to control COVID-19, the authors report several limitations of the review processes used:**<br>"Because of time constraints…we dually screened only 30% of the titles and abstracts; for the rest, we used single screening. A recent study showed that single abstract screening misses up to 13% of relevant studies (Gartlehner 2020). In addition, single review authors rated risk of bias, conducted data extraction and rated certainty of evidence. A second review author checked the plausibility of decisions and the correctness of data. Because these steps were not conducted dually and independently, we introduced some risk of error…Nevertheless, we are confident that none of these methodological limitations would change the overall conclusions of this review. Furthermore, we limited publications to English and Chinese languages. Because COVID-19 has become a rapidly evolving pandemic, we might have missed recent publications in languages of countries that have become heavily affected in the meantime (e.g. Italian or Spanish)." (88)<br>**Example 2: In a review examining the effects of regular inhaled therapies for patients with stable chronic obstructive pulmonary disease, the authors report several limitations of the review processes used:**<br>"We acknowledge several limitations…Although our network meta-analysis included all available randomized controlled trials, we could not conduct a subgroup analysis to identify a specific group of patients who could benefit from triple therapy more prominently…Because studies reporting information – such as eosinophil counts and chronic bronchitis – were fewer than expected, we could not generate a sufficient network for the sensitivity and meta-regression analyses. In addition, we did not evaluate the symptoms, use of rescue medication, quality of life, and lung function, which are other important outcomes." (89)<br>**Example 3: In a review examining the effects of red and processed meat consumption on risk of cardiometabolic and cancer outcomes, the authors report several limitations of the review processes used:**<br>"One of the primary limitations of our work is the heterogeneity of dietary patterns across studies. Although all patterns discriminated between participants with low and high intake of red and processed meat, other food and nutrient characteristics of dietary patterns and the quantity of red and processed meat consumed varied widely across studies. Moreover, the quantity of red and processed meat consumed differed across dietary patterns and studies. For example, one study compared 1.4 versus 3.5 servings of processed meat per week, whereas another compared 0.7 versus 4.9 servings per week. Such inconsistencies may have increased heterogeneity of meta-analyses and potentially reduced the magnitude of observed associations. Also, analyses of |

| Checklist item | Examples |
|---|---|
| | extreme categories of adherence may artificially inflate effect estimates and may not be indicative of effects observed at typical levels of adherence. Second, we were unable to analyze the data separately for red and processed meat because authors typically combined them or did not distinguish between them in primary studies." (90) |
| Item 23d. DISCUSSION: Discuss implications of the results for practice, policy, and future research. | **Example 1: In a review examining the effects of bystander programs on the prevention of sexual assault among adolescents and college students, the authors discuss the implications for practice given the evidence of benefit observed:**<br><br>"Implications for practice and policy: Findings from this review indicate that bystander programs have significant beneficial effects on bystander intervention behaviour. This provides important evidence of the effectiveness of mandated programs on college campuses. Additionally, the fact that our (preliminary) moderator analyses found program effects on bystander intervention to be similar for adolescents and college students suggests early implementation of bystander programs (i.e., in secondary schools with adolescents) may be warranted. Importantly, although we found that bystander programs had a significant beneficial effect on bystander intervention behaviour, we found no evidence that these programs had an effect on participants' sexual assault perpetration. Bystander programs may therefore be appropriate for targeting bystander behaviour, but may not be appropriate for targeting the behaviour of potential perpetrators. Additionally, effects of bystander programs on bystander intervention behaviour diminished by 6-month post-intervention. Thus, programs effects may be prolonged by the implementation of booster sessions conducted prior to 6 months post-intervention.<br><br>Implications for research: Findings from this review suggest there is a fairly strong body of research assessing the effects of bystander programs on attitudes and behaviors. However, there are a couple of important questions worth further exploration. First, according to one prominent logical model, bystander programs promote bystander intervention by fostering prerequisite knowledge and attitudes (Burn, 2009). Our meta-analysis provides inconsistent evidence of the effects of bystander programs on knowledge and attitudes, but promising evidence of short-term effects on bystander intervention. This casts uncertainty around the proposed relationship between knowledge/attitudes and bystander behavior. Although we were unable to assess these issues in the current review, this will be an important direction for future research. Our understanding of the causal mechanisms of program effects on bystander behavior would benefit from further analysis (e.g., path analysis mapping relationships between specific knowledge/attitude effects and bystander intervention). Second, bystander programs exhibit a great deal of content variability, most notably in framing sexual assault as a gendered or gender-neutral problem. That is, bystander programs tend to adopt one of two main approaches to addressing sexual assault: (a) presenting sexual assault as a gendered problem (overwhelmingly affecting women) or (b) presenting sexual assault as a gender-neutral problem (affecting women and men alike). Differential effects of these two types of programs remain largely unexamined. Our analysis indicated that (a) the sex of victims/perpetrators (i.e., portrayed in programs as gender neutral or male perpetrator and female victim) and (b) whether programs were implemented in mixed- or single-sex settings were not significant moderators of program effects on bystander |

| Checklist item | Examples |
|---|---|
| | intervention. However, these findings are limited to a single outcome and they should be considered preliminary, as they are based on a small sample (n = 11). Our understanding of the differential effects of gendered versus gender neutral programs would benefit from the design and implementation of high-quality primary studies that make direct comparisons between these two types of programs (e.g., RCTs comparing the effects of two active treatment arms that differ in their gendered approach). Finally, our systematic review and meta-analysis demonstrate the lack of global evidence concerning bystander program effectiveness. Our understanding of bystander programs' generalizability to non-US contexts would be greatly enhanced by high quality research conducted across the world." (81)<br><br>**Example 2: In a review examining the effects of trauma-informed approaches in schools, the authors discuss the implications for practice given the lack of evidence of benefit:**<br><br>"From this review, it seems like the most prudent thing for school leaders, policymakers, and school mental health professionals to do would be proceed with caution in their embrace of a trauma-informed approach as an overarching framework and conduct rigorous evaluation of this approach. We simply do not have the evidence (yet) to know if this works, and indeed, we do not know if using a trauma-informed approach could actually have unintended negative consequences for traumatized youth and school communities. We also do not have evidence of other potential costs in implementing this approach in schools, whether they be financial, academic, or other opportunity costs, and whether benefits outweigh the costs of implementing and maintaining this approach in schools. That said, calling for caution in adopting trauma-informed care in schools does not preclude schools from continuing to implement evidence-informed programs that target trauma symptoms in youth, or that they should simply wait for the research to provide unequivocal answers. The benefit of the trauma-informed approach being made freely available by SAMHSA and other policymakers is that these components can form the basis for a school (or school district) to begin to adapt and apply this approach in schools." (91)<br><br>**Example 3: In a review examining the effects of lenvatinib and sorafenib for differentiated thyroid cancer, the authors list implications for future research in order of priority:**<br><br>"In order of priority, the assessment group suggests the following further research priorities:<br><br>1. Clinical advice to the assessment group is that only radioactive iodine-refractory differentiated thyroid cancer patients experiencing symptoms, or those who have clinically significant progressive disease, are likely to be treated in routine clinical practice. Subgroup analyses suggest that the effects on progression-free survival are similar for patients treated with sorafenib regardless of whether they are symptomatic or asymptomatic. However, these findings are post hoc and include only a minority of symptomatic patients. It is unclear if other outcomes, such as overall survival, objective tumour response rate, adverse events and health-related quality of life, differ by symptomatic or asymptomatic disease. Future |

| Checklist item | Examples |
|---|---|
| | studies of patients should aim to include a greater proportion of patients with symptomatic disease and investigate possible differences. Consideration should be given to using the classification of patients as symptomatic or asymptomatic as a randomisation stratification factor.<br>2. It would be useful to record, and report, health-related quality of life outcomes from any future clinical study of lenvatinib and sorafenib. In particular, data should be collected, using the EQ-5D questionnaire, throughout the whole trial period, not only from patients whose disease has not progressed. Further research on health-related quality of life from treating patients who have symptomatic disease compared with those who do not is also required.<br>3. Currently, evidence does not allow a comparison of the effectiveness of treatment with lenvatinib with the effectiveness of treatment with sorafenib. A head-to-head trial considering these treatments and placebo would generate results that would be valuable to decision-makers.<br>4. It would be useful to explore how lenvatinib, sorafenib and best supportive care should be positioned in the treatment pathway." (92) |
| Item 24a. REGISTRATION AND PROTOCOL: Provide registration information for the review, including register name and registration number, or state that the review was not registered. | **Example 1: In a review examining the applicability of augmented reality in orthopaedic surgery, the authors report that the review was registered, specifying the register name (PROSPERO) and registration number:**<br>"…this systematic review has been registered in the international prospective register of systematic reviews (PROSPERO) under the registration number: CRD42019128569" (93)<br>**Example 2: In a review examining the dose-response relationship between exercise and cognitive function in older adults with and without cognitive impairment, the authors report that the review was registered, specifying the register name (Open Science Framework) and a URL to the register entry:**<br>"The current protocol is registered with the Open Science Framework (url: https://osf.io/qe43p/)" (94)<br>**Example 3: In a review examining the effects of routine anti-osteoporosis medication in men, the authors report that the review was not registered:**<br>"A protocol for this systematic review was developed before the research began; however, this review was not registered" (4)<br>**Example 4: In a review examining the effect of feedback on satisfaction with the outcome of task performance, the authors report that the review was not registered and indicate the potential limitations of not doing so:**<br>"The present meta-analysis was not registered online while it was in the planning stage. This of course increases the probability of an unplanned duplication, and does not allow a verification that review methods were carried out as planned." (95) |

| Checklist item | Examples |
|---|---|
| Item 24b. REGISTRATION AND PROTOCOL: Indicate where the review protocol can be accessed, or state that a protocol was not prepared. | **Example 1: In a review examining the effects of psychological interventions for fatigue in cancer survivors, the authors report that the protocol for the review is published and provide a citation for it:**<br>"The review protocol was registered with the International Prospective Register of Systematic Reviews (PROSPERO) database (registration number: CRD42014015219) and the protocol has been published [citation for protocol provided]." (96)<br>**Example 2: In a review examining psychotropic medication non-adherence and its associated factors among patients with major psychiatric disorders, the authors report that the protocol for the review is published and provide a citation for it:**<br>"…this systematic review and meta-analysis protocol has been published elsewhere [citation for the protocol provided]." (97) |
| Item 24c. REGISTRATION AND PROTOCOL: Describe and explain any amendments to information provided at registration or in the protocol. | **Example 1: In a review examining the effects of pharmacological interventions for the treatment of delirium in critically ill adults, the authors describe and explain several amendments to information provided in the protocol:**<br>"Differences between protocol and review: In our protocol (Burry 2015), we planned the primary outcome to be duration of delirium, defined as the time from which it was first identified to when it was first resolved (i.e. screened negative as defined by study authors (e.g. first negative screen, two consecutive screenings)), measured in days, and our secondary outcome to be the total duration of delirium, measured in days. There was far more variability in the definition of the outcome used than we had anticipated. Only two trials reported on the duration of delirium's first episode, and the remaining trials reported days with delirium, time in delirium, or total duration of delirium; most did not report when delirium was identified or how trial authors defined resolution of delirium. We therefore chose to report the total duration of delirium as our primary outcome and to pool the variable definitions. We added the outcome number of days in coma, as this outcome was reported in four trials, and we believed it important to include it in this review, as it is a newer outcome that is likely to be included in subsequent studies." (98)<br>**Example 2: In a review examining the effects of pharmacologic therapies on patients with idiopathic sudden sensorineural hearing loss, the authors describe and explain several amendments to information provided in the protocol:**<br>"We incurred no deviations from the *a priori* review protocol, with the exception of a minor modification of our modelling approach for: 1) continuous endpoints at baseline and at final follow-up with corresponding standard deviations (but without average changes and corresponding standard deviations per group) in certain studies; and 2) follow-up time due to variations in endpoints assessment time across studies." (99)<br>**Example 3: In a review examining the effects of deworming in non-pregnant adolescent girls and adult women, the authors describe and explain several amendments to information provided in the protocol:** |

| Checklist item | Examples |
| --- | --- |
| | "Differences from protocol: We modified the lower limit for age in our eligibility criteria from 12 years of age to 10 years of age because the age of adolescence was reduced. We used the WHO measures for severe anaemia, defined by haemoglobin levels < 80 g/L instead of < 70 g/L as stated in the protocol. We decided to add adverse events to our list of primary outcomes (instead of secondary) and we changed reinfection rate to a secondary outcome." (100)<br><br>**Example 4: In a review examining the effects of interventions for chronic pruritus of unknown origin, the authors describe and explain several amendments to information provided in the protocol:**<br><br>"Differences between protocol and review:<br><br>• Title: The original title of the protocol was "Interventions for pruritus of unknown cause"; it was changed to "Interventions for chronic pruritus of unknown origin" as this is currently the most familiar and widely used term among clinicians. We also changed the name of the condition throughout the review.<br>• Types of participants: Because the only included study had a (minority) subset of patients with a different diagnosis, we added to our inclusion criteria the following: "When we found studies with a subset of patients with a diagnosis of CPUO, we included them if data are presented separately for these patients, or if the majority (> 50%) of the included participants met the inclusion criteria. If data were not available for this subset of participants, we tried to retrieve this information from the investigators before excluding the study."<br>• Types of interventions: In the published version of the protocol, we defined "aprepitant" as a systemic intervention in representation of the pharmacological group "substance P and neurokinin 1 receptor (NK1R) antagonist"; therefore for the report of the review, we changed this in the inclusion criteria. We found no studies evaluating the prioritised comparisons: emollient creams, cooling lotions, topical corticosteroids, topical antidepressants, systemic antihistamines, systemic antidepressants, systemic anticonvulsants, and phototherapy. Therefore, instead we created three SoF tables for comparisons of the only study we included.<br>• Search methods: Due to the large number of excluded studies (n=67), we did not screen the bibliographies of excluded studies for further references to relevant reviews.<br>• Risk of bias assessment: We have updated the 'risk of bias' methods with the new tool ROB 2.0 in line with guidance from the new version of the Cochrane Handbook for Systematic Reviews of Interventions, and based on the protocol "Therapeutic interventions for alcohol dependence in non-inpatient settings: a systematic review and network meta-analysis" (Cheng 2017).<br>• Methods not implemented: Because this review included only one study, we could not perform any meta-analyses, and hence could not assess publication bias nor perform sensitivity analysis or subgroup analyses. We did not impute missing data because we considered missing data to be minimal (see Appendix 5, item 3.1)." (101) |

| Checklist item | Examples |
|---|---|
| Item 25. SUPPORT: Describe sources of financial or non-financial support for the review, and the role of the funders or sponsors in the review. | **Example 1: In a review examining the effects of screening for hepatitis C virus infection in adolescents and adults, the authors report receiving government agency funding to conduct the review, and describe the role of the funder and sponsor in the review:**<br>"Funding/Support: This research was funded under contract HHSA290201500009i, Task Order 7, from the Agency for Healthcare Research and Quality (AHRQ), US Department of Health and Human Services, under a contract to support the US Preventive Services Task Force (USPSTF). Role of the Funder/Sponsor: Investigators worked with USPSTF members and AHRQ staff to develop the scope, analytic framework, and key questions for this review. AHRQ had no role in study selection, quality assessment, or synthesis. AHRQ staff provided project oversight, reviewed the report to ensure that the analysis met methodological standards, and distributed the draft for peer review. Otherwise, AHRQ had no role in the conduct of the study; collection, management, analysis, and interpretation of the data; and preparation, review, or approval of the manuscript findings. The opinions expressed in this document are those of the authors and do not reflect the official position of AHRQ or the US Department of Health and Human Services." (102)<br>**Example 2: In a review examining the effects of switching from a gonadotropin-releasing hormone (GnRH) agonist to a GnRH antagonist in prostate cancer patients, the authors report receiving pharmaceutical funding to conduct one component of the review:**<br>"Funding for statistical analyses was provided by Ferring Pharmaceuticals. Dr. Toren is supported by a clinician-scientist award from Fonds de Recherche du Québec – Santé (#32774)." (103)<br>**Example 3: In a review examining the effects of mobile health applications for improving the sexual health outcomes among adults with chronic diseases, the authors report receiving no financial support for the review:**<br>"The authors received no financial support for the research, authorship and/or publication of this article." (104) |
| Item 26. COMPETING INTERESTS: Declare any competing interests of review authors. | **Example 1: In a review examining the effects of percutaneous vertebroplasty for osteoporotic vertebral compression fracture, the authors declare several competing interests, one of which was that they authored studies included in the review, and specify how they managed this competing interest in the review:**<br>"Declarations of interest: R Buchbinder was a principal investigator of Buchbinder 2009. D Kallmes was a principal investigator of Kallmes 2009 and Evans 2015. D Kallmes participated in IDE trial for Benvenue Medical spinal augmentation device. He is a stockholder, Marblehead Medical, LLC, Development of spine augmentation devices. He holds a spinal fusion patent license, unrelated to spinal augmentation/vertebroplasty. R Buchbinder and D Kallmes did not perform risk of bias assessments for their own or any other placebo-controlled trials included in the review." (105) |

| Checklist item | Examples |
|---|---|
| | **Example 2: In a review examining psychiatric morbidity and suicidal behaviour in low- and middle-income countries, the authors declare several competing interests:**<br><br>"Competing interests: I have read the journal's policy and the authors of this manuscript have the following competing interests: NK chairs and contributes to a number of guidelines for self-harm and suicidal behaviour and sits on the main government advisory group for suicide prevention in England. NK and DK also advised the Sri Lankan Ministry of Health on their suicide prevention strategy. NK receives research funding from government and charity sources. NK does not receive industry funding or personal remuneration." (106)<br><br>**Example 3: In a review examining the prevalence of traumatic brain injury in homeless and marginally housed individuals, the authors declare several competing interests:**<br><br>"Declaration of interests: NDS has sat on the paid advisory board of Highmark Interactive, received consulting or speaking fees from WorkSafeBC and Yukon WCB, the National Hockey League, and Major League Soccer, and has received fees for expert testimony in neuropsychology. WGH has received consulting fees or sat on paid advisory boards for the Canadian Agency for Drugs and Technology in Health, AlphaSights, Guidepoint, In Silico, Translational Life Sciences, Otsuka, Lundbeck, and Newron. WJP is the founder and chief executive officer of Translational Life Sciences, an early stage biotechnology company. He is also on the scientific advisory board of Medipure Pharmaceuticals and Vitality Biopharma, and in the past has been on the board of directors for Abattis Bioceuticals and on the advisory board for Vinergy Resources; these companies are early stage biotechnology enterprises with no relation to brain injury. All other authors declare no competing interests." (107)<br><br>**Example 4: In a review examining the effects of alcohol consumption on the incidence of cardiovascular diseases, the authors declare having no competing interests:**<br><br>"Competing interest: The authors declare that they have no competing interests". (108) |
| Item 27. AVAILABILITY OF DATA, CODE AND OTHER MATERIALS: Report which of the following are publicly available and where they can be found: template data | **Example 1: In a review examining the effects of education on intelligence, the authors report that the origin of each data point is described in a spreadsheet made publicly available in the Open Science Framework repository, and provide a URL for readers to access the file (the repository also included datasets and analytic code for the review):**<br><br>"All meta-analytic data and all codebooks and analysis scripts (for Mplus and R) are publicly available at the study's associated page on the Open Science Framework (https://osf.io/r8a24/)...The precise sources (table, section, or paragraph) for each estimate are described in notes in the master data spreadsheet, available on the Open Science Framework page for this study (https://osf.io/r8a24/)" (109) |

| Checklist item | Examples |
|---|---|
| collection forms; data extracted from included studies; data used for all analyses; analytic code; any other materials used in the review. | **Example 2: In a review examining the effects of self-management smartphone-based apps for post-traumatic stress disorder symptoms, the authors report that the data and analytic code are publicly available in the Open Science Framework repository and provide a DOI for readers to access the files:**<br>“All data and code are stored on a repository of the Open Science Framework (doi: 10.17605/OSF.IO/DZJT7)” (110)<br>**Example 3: In a review examining the effects of specialised treatments for anorexia nervosa, the authors report that the data and analytic code are publicly available in the Open Science Framework repository and provide a URL for readers to access the files:**<br>“The dataset and script to perform the analyses are available at https://osf.io/q7v2d/?view_only=c3cdaf346298411eab9ed15e863c9f21.” (111) |

**References**

1. Khodashahi M, Rezaieyazdi Z, Sahebari M. Comparison of the Therapeutic Effects of Rivaroxaban Versus Warfarin in Antiphospholipid Syndrome: A Systematic Review. Archives of rheumatology. 2020;35(1):107-16.
2. Ghayour-Najafabadi M, Memari AH, Hosseini L, Shariat A, Cleland JA. Repetitive Transcranial Magnetic Stimulation for the Treatment of Lower Limb Dysfunction in Patients Poststroke: A Systematic Review with Meta-Analysis. Journal of stroke and cerebrovascular diseases : the official journal of National Stroke Association. 2019;28(12):104412.
3. Hauser W, Welsch P, Klose P, Radbruch L, Fitzcharles MA. Efficacy, tolerability and safety of cannabis-based medicines for cancer pain : A systematic review with meta-analysis of randomised controlled trials. Schmerz (Berlin, Germany). 2019;33(5):424-36.
4. Zeng LF, Pan BQ, Liang GH, Luo MH, Cao Y, Guo D, et al. Does Routine Anti-Osteoporosis Medication Lower the Risk of Fractures in Male Subjects? An Updated Systematic Review With Meta-Analysis of Clinical Trials. Frontiers in pharmacology. 2019;10:882.
5. Keynejad RC, Hanlon C, Howard LM. Psychological interventions for common mental disorders in women experiencing intimate partner violence in low-income and middle-income countries: a systematic review and meta-analysis. The Lancet Psychiatry. 2020;7(2):173-90.
6. Fodor LA, Georgescu R, Cuijpers P, Szamoskozi Ş, David D, Furukawa TA, et al. Efficacy of cognitive bias modification interventions in anxiety and depressive disorders: a systematic review and network meta-analysis. The lancet Psychiatry. 2020;7(6):506-14.
7. Raban MZ, Gasparini C, Li L, Baysari MT, Westbrook JI. Effectiveness of interventions targeting antibiotic use in long-term aged care facilities: a systematic review and meta-analysis. BMJ Open. 2020;10(1).
8. Huang L, Trieu K, Yoshimura S, Neal B, Woodward M, Campbell NRC, et al. Effect of dose and duration of reduction in dietary sodium on blood pressure levels: systematic review and meta-analysis of randomised trials. BMJ. 2020;368:m315.
9. Chu DK, Akl EA, Duda S, Solo K, Yaacoub S, Schünemann HJ. Physical distancing, face masks, and eye protection to prevent person-to-person transmission of SARS-CoV-2 and COVID-19: a systematic review and meta-analysis. Lancet. 2020;395(10242):1973-87.
10. Welch VA, Ghogomu E, Hossain A, Riddle A, Gaffey M, Arora P, et al. Mass deworming for improving health and cognition of children in endemic helminth areas: A systematic review and individual participant data network meta-analysis. Campbell Systematic Reviews. 2019;15(4):e1058.

11. Brazzelli M, Javanbakht M, Imamura M, Hudson J, Moloney E, Becker F, et al. Surgical treatments for women with stress urinary incontinence: the ESTER systematic review and economic evaluation. Health Technol Assess. 2019;23(14):1-306.
12. Remington J, Winters K. Effectiveness of dietary inorganic nitrate for lowering blood pressure in hypertensive adults: a systematic review. JBI database of systematic reviews and implementation reports. 2019;17(3):365-89.
13. Verhoef LM, van den Bemt BJF, van der Maas A, Vriezekolk JE, Hulscher ME, van den Hoogen FHJ, et al. Down-titration and discontinuation strategies of tumour necrosis factor–blocking agents for rheumatoid arthritis in patients with low disease activity. Cochrane Database of Systematic Reviews. 2019(5):CD010455.
14. Chou R, Evans C, Hoverman A, Sun C, Dana T, Bougatsos C, et al. U.S. Preventive Services Task Force Evidence Syntheses, formerly Systematic Evidence Reviews. Pre-Exposure Prophylaxis for the Prevention of HIV Infection: A Systematic Review for the US Preventive Services Task Force. Rockville (MD): Agency for Healthcare Research and Quality (US); 2019.
15. Dol J, Richardson B, Tomblin Murphy G, Aston M, McMillan D, Campbell-Yeo M. Impact of mobile health (mHealth) interventions during the perinatal period for mothers in low- and middle-income countries: a systematic review. JBI database of systematic reviews and implementation reports. 2019;17(8):1634-67.
16. Hamel C, Ahmadzai N, Beck A, Thuku M, Skidmore B, Pussegoda K, et al. Screening for esophageal adenocarcinoma and precancerous conditions (dysplasia and Barrett's esophagus) in patients with chronic gastroesophageal reflux disease with or without other risk factors: two systematic reviews and one overview of reviews to inform a guideline of the Canadian Task Force on Preventive Health Care (CTFPHC). Systematic reviews. 2020;9(1):20.
17. Fisher CA, Skocic S, Rutherford KA, Hetrick SE. Family therapy approaches for anorexia nervosa. Cochrane Database of Systematic Reviews. 2019(5).
18. Odor PM, Bampoe S, Gilhooly D, Creagh-Brown B, Moonesinghe SR. Perioperative interventions for prevention of postoperative pulmonary complications: systematic review and meta-analysis. BMJ. 2020;368:m540.
19. Dobler CC, Morrow AS, Farah MH, Beuschel B, Majzoub AM, Wilson ME, et al. AHRQ Comparative Effectiveness Reviews. Pharmacologic and Nonpharmacologic Therapies in Adult Patients With Exacerbation of COPD: A Systematic Review. Rockville (MD): Agency for Healthcare Research and Quality (US); 2019.

20. Hollands GJ, Carter P, Anwer S, King SE, Jebb SA, Ogilvie D, et al. Altering the availability or proximity of food, alcohol, and tobacco products to change their selection and consumption. Cochrane Database of Systematic Reviews. 2019(9):CD012573.
21. Jay MA, Mc Grath-Lone L. Educational outcomes of children in contact with social care in England: a systematic review. Systematic reviews. 2019;8(1):155.
22. von Philipsborn P, Stratil JM, Burns J, Busert LK, Pfadenhauer LM, Polus S, et al. Environmental interventions to reduce the consumption of sugar-sweetened beverages and their effects on health. Cochrane Database of Systematic Reviews. 2019(6):CD012292.
23. Gopalani SV, Sedani AE, Janitz AE, Clifton SC, Stoner J, Peck J, et al. HPV vaccination and Native Americans: protocol for a systematic review of factors associated with HPV vaccine uptake among American Indians and Alaska Natives in the USA. BMJ Open. 2020;10(9):e035658.
24. Freemantle N, Ginsberg DA, McCool R, Fleetwood K, Arber M, Khalaf K, et al. Comparative assessment of onabotulinumtoxinA and mirabegron for overactive bladder: an indirect treatment comparison. BMJ Open. 2016;6(2):e009122.
25. Bomhof-Roordink H, Gartner FR, Stiggelbout AM, Pieterse AH. Key components of shared decision making models: a systematic review. BMJ Open. 2019;9(12):e031763.
26. Brennan SE, McDonald S, Page MJ, Reid J, Ward S, Forbes AB, et al. Long-term effects of alcohol consumption on cognitive function: a systematic review and dose-response analysis of evidence published between 2007 and 2018. Systematic reviews. 2020;9(1):33.
27. Claire R, Chamberlain C, Davey MA, Cooper SE, Berlin I, Leonardi-Bee J, et al. Pharmacological interventions for promoting smoking cessation during pregnancy. Cochrane Database of Systematic Reviews. 2020(3):CD010078.
28. Troelstra SA, Coenen P, Boot CR, Harting J, Kunst AE, van der Beek AJ. Smoking and sickness absence: a systematic review and meta-analysis. Scand J Work Environ Health. 2020;46(1):5-18.
29. Surace SJ, Deitch J, Johnston RV, Buchbinder R. Shock wave therapy for rotator cuff disease with or without calcification. Cochrane Database of Systematic Reviews. 2020(3):CD008962.
30. Wolfenden L, Barnes C, Jones J, Finch M, Wyse RJ, Kingsland M, et al. Strategies to improve the implementation of healthy eating, physical activity and obesity prevention policies, practices or programmes within childcare services. Cochrane Database of Systematic Reviews. 2020(2):CD011779.

31. Watt JA, Goodarzi Z, Veroniki AA, Nincic V, Khan PA, Ghassemi M, et al. Comparative Efficacy of Interventions for Aggressive and Agitated Behaviors in Dementia: A Systematic Review and Network Meta-analysis. Ann Intern Med. 2019.
32. Allida S, Cox KL, Hsieh CF, Lang H, House A, Hackett ML. Pharmacological, psychological, and non-invasive brain stimulation interventions for treating depression after stroke. Cochrane Database of Systematic Reviews. 2020(1):CD003437.
33. Morgan EH, Schoonees A, Sriram U, Faure M, Seguin-Fowler RA. Caregiver involvement in interventions for improving children's dietary intake and physical activity behaviors. Cochrane Database of Systematic Reviews. 2020(1):CD012547.
34. Cohn EG, Kakar S, Perkins C, Steinbach R, Edwards P. Red light camera interventions for reducing traffic violations and traffic crashes: A systematic review. Campbell Systematic Reviews. 2020;16(2):e1091.
35. Kunzler AM, Helmreich I, König J, Chmitorz A, Wessa M, Binder H, et al. Psychological interventions to foster resilience in healthcare students. Cochrane Database of Systematic Reviews. 2020(7):CD013684.
36. Gutierrez M, Rodriguez JL, Zamora-de La Cruz D, Flores Pimentel MA, Jimenez-Corona A, Novak LC, et al. Pars plana vitrectomy combined with scleral buckle versus pars plana vitrectomy for giant retinal tear. Cochrane Database of Systematic Reviews. 2019(12):CD012646.
37. Clement NS, Oliver TRW, Shiwani H, Sanner JRF, Mulvaney CA, Atiomo W. Metformin for endometrial hyperplasia. Cochrane Database of Systematic Reviews. 2017(10):CD012214.
38. Munthe-Kaas HM, Berg RC, Blaasvær N. Effectiveness of interventions to reduce homelessness: a systematic review and meta-analysis. Campbell Systematic Reviews. 2018;14(1):1-281.
39. Das JK, Salam RA, Mahmood SB, Moin A, Kumar R, Mukhtar K, et al. Food fortification with multiple micronutrients: impact on health outcomes in general population. Cochrane Database of Systematic Reviews. 2019(12):CD011400.
40. Pantoja T, Grimshaw JM, Colomer N, Castañon C, Leniz Martelli J. Manually-generated reminders delivered on paper: effects on professional practice and patient outcomes. Cochrane Database of Systematic Reviews. 2019(12):CD001174.
41. Burns J, Boogaard H, Polus S, Pfadenhauer LM, Rohwer AC, van Erp AM, et al. Interventions to reduce ambient particulate matter air pollution and their effect on health. Cochrane Database of Systematic Reviews. 2019(5):CD010919.

42. Hunter BM, Harrison S, Portela A, Bick D. The effects of cash transfers and vouchers on the use and quality of maternity care services: A systematic review. PloS one. 2017;12(3):e0173068-e.
43. Kyburz KS, Eliades T, Papageorgiou SN. What effect does functional appliance treatment have on the temporomandibular joint? A systematic review with meta-analysis. Progress in Orthodontics. 2019;20(1):32.
44. Kock L, Brown J, Hiscock R, Tattan-Birch H, Smith C, Shahab L. Individual-level behavioural smoking cessation interventions tailored for disadvantaged socioeconomic position: a systematic review and meta-regression. The Lancet Public Health. 2019;4(12):e628-e44.
45. Cucherat M, Haugh MC, Gooch M, Boissel JP. Evidence of clinical efficacy of homeopathy. A meta-analysis of clinical trials. HMRAG. Homeopathic Medicines Research Advisory Group. Eur J Clin Pharmacol. 2000;56(1):27-33.
46. Wang N, Fulcher J, Abeysuriya N, Park L, Kumar S, Di Tanna GL, et al. Intensive LDL cholesterol-lowering treatment beyond current recommendations for the prevention of major vascular events: a systematic review and meta-analysis of randomised trials including 327 037 participants. The Lancet Diabetes & Endocrinology. 2020;8(1):36-49.
47. Roth DE, Leung M, Mesfin E, Qamar H, Watterworth J, Papp E. Vitamin D supplementation during pregnancy: state of the evidence from a systematic review of randomised trials. BMJ. 2017;359:j5237.
48. Brown TJ, Brainard J, Song F, Wang X, Abdelhamid A, Hooper L. Omega-3, omega-6, and total dietary polyunsaturated fat for prevention and treatment of type 2 diabetes mellitus: systematic review and meta-analysis of randomised controlled trials. BMJ. 2019;366:l4697.
49. Karjalainen TV, Jain NB, Heikkinen J, Johnston RV, Page CM, Buchbinder R. Surgery for rotator cuff tears. Cochrane Database of Systematic Reviews. 2019(12):CD013502.
50. Maguire MJ, Nevitt SJ. Treatments for seizures in catamenial (menstrual-related) epilepsy. Cochrane Database of Systematic Reviews. 2019(10).
51. Merino J, Guasch-Ferré M, Ellervik C, Dashti HS, Sharp SJ, Wu P, et al. Quality of dietary fat and genetic risk of type 2 diabetes: individual participant data meta-analysis. BMJ. 2019;366:l4292.
52. Walther A, Breidenstein J, Miller R. Association of Testosterone Treatment With Alleviation of Depressive Symptoms in Men: A Systematic Review and Meta-analysis. JAMA Psychiatry. 2019;76(1):31-40.
53. Mudano AS, Tugwell P, Wells GA, Singh JA. Tai Chi for rheumatoid arthritis. Cochrane Database of Systematic Reviews. 2019(9):CD004849.

54. El Moheb M, Nicolas J, Khamis AM, Iskandarani G, Akl EA, Refaat M. Implantable cardiac defibrillators for people with non-ischaemic cardiomyopathy. Cochrane Database of Systematic Reviews. 2018(12).
55. Kondo KK, Morasco BJ, Nugent SM, Ayers CK, O'Neil ME, Freeman M, et al. Pharmacotherapy for the Treatment of Cannabis Use Disorder: A Systematic Review. Ann Intern Med. 2020.
56. Chambergo-Michilot D, Tellez WA, Becerra-Chauca N, Zafra-Tanaka JH, Taype-Rondan A. Text message reminders for improving sun protection habits: A systematic review. PloS one. 2020;15(5):e0233220.
57. Lunney M, Ruospo M, Natale P, Quinn RR, Ronksley PE, Konstantinidis I, et al. Pharmacological interventions for heart failure in people with chronic kidney disease. Cochrane Database of Systematic Reviews. 2020(2):CD012466.
58. Meernik C, Baker HM, Kowitt SD, Ranney LM, Goldstein AO. Impact of non-menthol flavours in e-cigarettes on perceptions and use: an updated systematic review. BMJ open. 2019;9(10):e031598.
59. Mayo-Wilson E, Li T, Fusco N, Bertizzolo L, Canner JK, Cowley T, et al. Cherry-picking by trialists and meta-analysts can drive conclusions about intervention efficacy. J Clin Epidemiol. 2017;91:95-110.
60. Kayssi A, Al-Jundi W, Papia G, Kucey DS, Forbes T, Rajan DK, et al. Drug-eluting balloon angioplasty versus uncoated balloon angioplasty for the treatment of in-stent restenosis of the femoropopliteal arteries. Cochrane Database of Systematic Reviews. 2019(1):CD012510.
61. Jansen EEL, Zielonke N, Gini A, Anttila A, Segnan N, Voko Z, et al. Effect of organised cervical cancer screening on cervical cancer mortality in Europe: a systematic review. Eur J Cancer. 2020;127:207-23.
62. Barker AL, Soh SE, Sanders KM, Pasco J, Khosla S, Ebeling PR, et al. Aspirin and fracture risk: a systematic review and exploratory meta-analysis of observational studies. BMJ Open. 2020;10(2):e026876.
63. Saccone G, Berghella V. Antenatal corticosteroids for maturity of term or near term fetuses: systematic review and meta-analysis of randomized controlled trials. BMJ. 2016;355:i5044.
64. Coxeter P, Del Mar CB, McGregor L, Beller EM, Hoffmann TC. Interventions to facilitate shared decision making to address antibiotic use for acute respiratory infections in primary care. Cochrane Database of Systematic Reviews. 2015(11).
65. Gupta T, Kannan S, Ghosh-Laskar S, Agarwal JP. Systematic review and meta-analyses of intensity-modulated radiation therapy versus conventional two-dimensional and/or or

three-dimensional radiotherapy in curative-intent management of head and neck squamous cell carcinoma. PloS one. 2018;13(7):e0200137.

66. Feng Q, Zhou A, Zou H, Ingle S, May MT, Cai W, et al. Quadruple versus triple combination antiretroviral therapies for treatment naive people with HIV: systematic review and meta-analysis of randomised controlled trials. BMJ. 2019;366:l4179.

67. Musini VM, Lawrence KAK, Fortin PM, Bassett K, Wright JM. Blood pressure lowering efficacy of renin inhibitors for primary hypertension. Cochrane Database of Systematic Reviews. 2017(4):CD007066.

68. Feltner C, Weber RP, Stuebe A, Grodensky CA, Orr C, Viswanathan M. AHRQ Comparative Effectiveness Reviews. Breastfeeding Programs and Policies, Breastfeeding Uptake, and Maternal Health Outcomes in Developed Countries. Rockville (MD): Agency for Healthcare Research and Quality (US); 2018.

69. Sanders GD, Lowenstern A, Borre E, Chatterjee R, Goode A, Sharan L, et al. AHRQ Comparative Effectiveness Reviews. Stroke Prevention in Patients With Atrial Fibrillation: A Systematic Review Update. Rockville (MD): Agency for Healthcare Research and Quality (US); 2018.

70. Neufeld KJ, Needham DM, Oh ES, Wilson LM, Nikooie R, Zhang A, et al. AHRQ Comparative Effectiveness Reviews. Antipsychotics for the Prevention and Treatment of Delirium. Rockville (MD): Agency for Healthcare Research and Quality (US); 2019.

71. Gelbenegger G, Postula M, Pecen L, Halvorsen S, Lesiak M, Schoergenhofer C, et al. Aspirin for primary prevention of cardiovascular disease: a meta-analysis with a particular focus on subgroups. BMC medicine. 2019;17(1):198.

72. Regnaux JP, Davergne T, Palazzo C, Roren A, Rannou F, Boutron I, et al. Exercise programmes for ankylosing spondylitis. Cochrane Database of Systematic Reviews. 2019(10):CD011321.

73. Shrestha N, Kukkonen-Harjula KT, Verbeek JH, Ijaz S, Hermans V, Pedisic Z. Workplace interventions for reducing sitting at work. Cochrane Database of Systematic Reviews. 2018(12):CD010912.

74. Sterne JAC, Murthy S, Diaz JV, Slutsky AS, Villar J, Angus DC, et al. Association Between Administration of Systemic Corticosteroids and Mortality Among Critically Ill Patients With COVID-19: A Meta-analysis. JAMA. 2020.

75. Backhouse A, Ukoumunne OC, Richards DA, McCabe R, Watkins R, Dickens C. The effectiveness of community-based coordinating interventions in dementia care: a meta-analysis and subgroup analysis of intervention components. BMC Health Serv Res. 2017;17(1):717-.

76. Niederer D, Mueller J. Sustainability effects of motor control stabilisation exercises on pain and function in chronic nonspecific low back pain patients: A systematic review with meta-analysis and meta-regression. PloS one. 2020;15(1):e0227423.
77. Yanes JA, McKinnell ZE, Reid MA, Busler JN, Michel JS, Pangelinan MM, et al. Effects of cannabinoid administration for pain: A meta-analysis and meta-regression. Exp Clin Psychopharmacol. 2019;27(4):370-82.
78. Ochen Y, Beks RB, van Heijl M, Hietbrink F, Leenen LPH, van der Velde D, et al. Operative treatment versus nonoperative treatment of Achilles tendon ruptures: systematic review and meta-analysis. BMJ. 2019;364:k5120.
79. Chau S, Herrmann N, Ruthirakuhan MT, Chen JJ, Lanctot KL. Latrepirdine for Alzheimer's disease. The Cochrane database of systematic reviews. 2015(4):CD009524.
80. Williams T, Hattingh CJ, Kariuki CM, Tromp SA, van Balkom AJ, Ipser JC, et al. Pharmacotherapy for social anxiety disorder (SAnD). Cochrane Database of Systematic Reviews. 2017(10):CD001206.
81. Kettrey HH, Marx RA, Tanner-Smith EE. Effects of bystander programs on the prevention of sexual assault among adolescents and college students: A systematic review. Campbell Systematic Reviews. 2019;15(1-2):e1013.
82. De Crescenzo F, D'Alò GL, Morgano GP, Minozzi S, Mitrova Z, Saulle R, et al. Impact of polyunsaturated fatty acids on patient-important outcomes in children and adolescents with autism spectrum disorder: a systematic review. Health Qual Life Outcomes. 2020;18(1):28.
83. Stone R, de Hoop T, Coombes A, Nakamura P. What works to improve early grade literacy in Latin America and the Caribbean? A systematic review and meta-analysis. Campbell Systematic Reviews. 2020;16(1):e1067.
84. Fleming P, McGilloway S, Hernon M, Furlong M, O'Doherty S, Keogh F, et al. Individualized funding interventions to improve health and social care outcomes for people with a disability: A mixed-methods systematic review. Campbell Systematic Reviews. 2019;15(1-2):e1008.
85. Kelly JF, Humphreys K, Ferri M. Alcoholics Anonymous and other 12-step programs for alcohol use disorder. Cochrane Database of Systematic Reviews. 2020(3):CD012880.
86. Ghasemiesfe M, Barrow B, Leonard S, Keyhani S, Korenstein D. Association Between Marijuana Use and Risk of Cancer: A Systematic Review and Meta-analysis. JAMA network open. 2019;2(11):e1916318.
87. Sabitova A, McGranahan R, Altamore F, Jovanovic N, Windle E, Priebe S. Indicators Associated With Job Morale Among Physicians and Dentists in Low-Income and Middle-

Income Countries: A Systematic Review and Meta-analysis. JAMA network open. 2020;3(1):e1913202.

88. Nussbaumer-Streit B, Mayr V, Dobrescu AI, Chapman A, Persad E, Klerings I, et al. Quarantine alone or in combination with other public health measures to control COVID-19: a rapid review. The Cochrane database of systematic reviews. 2020;4(4):CD013574.

89. Lee HW, Park J, Jo J, Jang EJ, Lee CH. Comparisons of exacerbations and mortality among regular inhaled therapies for patients with stable chronic obstructive pulmonary disease: Systematic review and Bayesian network meta-analysis. PLoS medicine. 2019;16(11):e1002958.

90. Vernooij RWM, Zeraatkar D, Han MA, El Dib R, Zworth M, Milio K, et al. Patterns of Red and Processed Meat Consumption and Risk for Cardiometabolic and Cancer Outcomes: A Systematic Review and Meta-analysis of Cohort Studies. Ann Intern Med. 2019.

91. Maynard BR, Farina A, Dell NA, Kelly MS. Effects of trauma-informed approaches in schools: A systematic review. Campbell Systematic Reviews. 2019;15(1-2):e1018.

92. Fleeman N, Houten R, Bagust A, Richardson M, Beale S, Boland A, et al. Lenvatinib and sorafenib for differentiated thyroid cancer after radioactive iodine: a systematic review and economic evaluation. Health Technol Assess. 2020;24(2):1-180.

93. Jud L, Fotouhi J, Andronic O, Aichmair A, Osgood G, Navab N, et al. Applicability of augmented reality in orthopedic surgery - A systematic review. BMC Musculoskelet Disord. 2020;21(1):103.

94. Sanders LMJ, Hortobágyi T, la Bastide-van Gemert S, van der Zee EA, van Heuvelen MJG. Dose-response relationship between exercise and cognitive function in older adults with and without cognitive impairment: A systematic review and meta-analysis. PloS one. 2019;14(1):e0210036.

95. Furenes MI, Røislien J, Gjerald O, Furunes T, Øgaard T. A systematic review and meta-analysis: the effect of feedback on satisfaction with the outcome of task performance. Heliyon. 2019;5(11):e02847-e.

96. Corbett TK, Groarke A, Devane D, Carr E, Walsh JC, McGuire BE. The effectiveness of psychological interventions for fatigue in cancer survivors: systematic review of randomised controlled trials. Systematic reviews. 2019;8(1):324.

97. Semahegn A, Torpey K, Manu A, Assefa N, Tesfaye G, Ankomah A. Psychotropic medication non-adherence and its associated factors among patients with major psychiatric disorders: a systematic review and meta-analysis. Systematic reviews. 2020;9(1):17.

98. Burry L, Hutton B, Williamson DR, Mehta S, Adhikari NKJ, Cheng W, et al. Pharmacological interventions for the treatment of delirium in critically ill adults. Cochrane Database of Systematic Reviews. 2019(9):CD011749.

99. Ahmadzai N, Kilty S, Cheng W, Esmaeilisaraji L, Wolfe D, Bonaparte JP, et al. A systematic review and network meta-analysis of existing pharmacologic therapies in patients with idiopathic sudden sensorineural hearing loss. PloS one. 2019;14(9):e0221713.

100. Tanjong Ghogomu E, Suresh S, Rayco-Solon P, Hossain A, McGowan J, Peña-Rosas JP, et al. Deworming in non-pregnant adolescent girls and adult women: a systematic review and meta-analysis. Systematic reviews. 2018;7(1):239.

101. Andrade A, Kuah CY, Martin-Lopez JE, Chua S, Shpadaruk V, Sanclemente G, et al. Interventions for chronic pruritus of unknown origin. Cochrane Database of Systematic Reviews. 2020(1):CD013128.

102. Chou R, Dana T, Fu R, Zakher B, Wagner J, Ramirez S, et al. Screening for Hepatitis C Virus Infection in Adolescents and Adults: Updated Evidence Report and Systematic Review for the US Preventive Services Task Force. JAMA. 2020.

103. Atchia KS, Wallis CJD, Fleshner N, Toren P. Switching from a gonadotropin-releasing hormone (GnRH) agonist to a GnRH antagonist in prostate cancer patients: A systematic review and meta-analysis. Can Urol Assoc J. 2020;14(2):36-41.

104. Karim H, Choobineh H, Kheradbin N, Ravandi MH, Naserpor A, Safdari R. Mobile health applications for improving the sexual health outcomes among adults with chronic diseases: A systematic review. Digit Health. 2020;6:2055207620906956.

105. Buchbinder R, Johnston RV, Rischin KJ, Homik J, Jones CA, Golmohammadi K, et al. Percutaneous vertebroplasty for osteoporotic vertebral compression fracture. Cochrane Database of Systematic Reviews. 2018(11):CD006349.

106. Knipe D, Williams AJ, Hannam-Swain S, Upton S, Brown K, Bandara P, et al. Psychiatric morbidity and suicidal behaviour in low- and middle-income countries: A systematic review and meta-analysis. PLoS medicine. 2019;16(10):e1002905.

107. Stubbs JL, Thornton AE, Sevick JM, Silverberg ND, Barr AM, Honer WG, et al. Traumatic brain injury in homeless and marginally housed individuals: a systematic review and meta-analysis. The Lancet Public Health. 2020;5(1):e19-e32.

108. Yoon S-J, Jung J-G, Lee S, Kim J-S, Ahn S-K, Shin E-S, et al. The protective effect of alcohol consumption on the incidence of cardiovascular diseases: is it real? A systematic review and meta-analysis of studies conducted in community settings. BMC Public Health. 2020;20(1):90.

109. Ritchie SJ, Tucker-Drob EM. How Much Does Education Improve Intelligence? A Meta-Analysis. Psychol Sci. 2018;29(8):1358-69.

110. Goreis A, Felnhofer A, Kafka JX, Probst T, Kothgassner OD. Efficacy of Self-Management Smartphone-Based Apps for Post-traumatic Stress Disorder Symptoms: A Systematic Review and Meta-Analysis. Front Neurosci. 2020;14:3-.

111. Murray SB, Quintana DS, Loeb KL, Griffiths S, Le Grange D. Treatment outcomes for anorexia nervosa: a systematic review and meta-analysis of randomized controlled trials. Psychol Med. 2019;49(4):535-44.
